# Synthesis, Molecular Structure, Metabolic Stability and QSAR Studies of a Novel Series of Anticancer *N*-Acylbenzenesulfonamides

**DOI:** 10.3390/molecules201019101

**Published:** 2015-10-21

**Authors:** Beata Żołnowska, Jarosław Sławiński, Mariusz Belka, Tomasz Bączek, Anna Kawiak, Jarosław Chojnacki, Aneta Pogorzelska, Krzysztof Szafrański

**Affiliations:** 1 Department of Organic Chemistry, Medical University of Gdańsk, Al. Gen. J. Hallera 107, 80-416 Gdańsk, Poland; E-Mails: zolnowska@gumed.edu.pl (B.Ż.); anetapogorzelska@gumed.edu.pl (A.P.); k.szafranski@gumed.edu.pl (K.S.); 2 Department of Pharmaceutical Chemistry, Medical University of Gdańsk, Al. Gen. J. Hallera 107, 80-416 Gdańsk, Poland; E-Mails: mariusz.belka@gumed.edu.pl (M.B.); tbaczek@gumed.edu.pl (T.B.); 3 Department of Biotechnology, Intercollegiate Faculty of Biotechnology, University of Gdańsk and Medical University of Gdańsk, ul. Kładki 24, 80-822 Gdańsk, Poland; E-Mail: kawiak@biotech.ug.pl; 4 Department of Human Physiology, Medical University of Gdańsk, ul. Tuwima 15, 80-210 Gdańsk, Poland; 5 Department of Inorganic Chemistry, Gdańsk University of Technology, Narutowicza 11/12, 80-233 Gdańsk, Poland; E-Mail: jarekch@pg.gda.pl

**Keywords:** sulfonamide, synthesis, anticancer, QSAR, metabolic stability

## Abstract

A series of novel *N*-acyl-4-chloro-5-methyl-2-(R^1^-methylthio)benzenesulfonamides **18**–**47** have been synthesized by the reaction of *N*-[4-chloro-5-methyl-2-(R1-methylthio)benzenesulfonyl]cyanamide potassium salts with appropriate carboxylic acids. Some of them showed anticancer activity toward the human cancer cell lines MCF-7, HCT-116 and HeLa, with the growth percentages (GPs) in the range from 7% to 46%. Quantitative structure-activity relationship (QSAR) studies on the cytotoxic activity of *N*-acylsulfonamides toward MCF-7, HCT-116 and HeLa were performed by using topological, ring and charge descriptors based on the stepwise multiple linear regression technique (MLR). The QSAR studies revealed three predictive and statistically significant models for the investigated compounds. The results obtained with these models indicated that the anticancer activity of *N*-acylsulfonamides depends on topological distances, number of ring system, maximum positive charge and number of atom-centered fragments. The metabolic stability of the selected compounds had been evaluated on pooled human liver microsomes and NADPH, both R^1^ and R^2^ substituents of the *N*-acylsulfonamides simultaneously affected them.

## 1. Introduction

Chemotherapeutics are the most important tool in the fight against cancer. For decades scientists have discovered many anticancer drugs by designing low-molecular weight compounds with the ability to bind to a preselected protein target or by screening small molecules for their ability to modulate a biological pathway in cells or organisms, or without regard for any particular protein target [1,2]. However, the question remains whether these drugs will offer lasting survival advantages to the patients in the light of the problem with of tumor unpredictability and drug resistance. A promising solution that is a focus of contemporary medicinal chemistry is the development of potent and selective anticancer drugs. Many of these new agents are being tested in combination with therapies currently used to treat specific cancers and usually include cytotoxic drugs. On the other hand, these newer agents are affording novel ways to mechanistically attack cancer, even if one cannot realize efficacy without toxicity [2].

*N*-Acylsulfonamides constitute a class of organic compounds with various types of pharmacological activities such as antibacterial inhibitors for tRNA synthetase [3], antagonists for angiotensin II [4], agents for the treatment of Alzheimer’s desease [5], and osteoporosis [6]. In the last decade, particular attention has been focused on *N*-acylsulfonamides as cyclooxygenase [7], tubulin [8] and cyclin-dependent kinases [9] inhibitors with antiproliferative activity.

**Figure 1:**
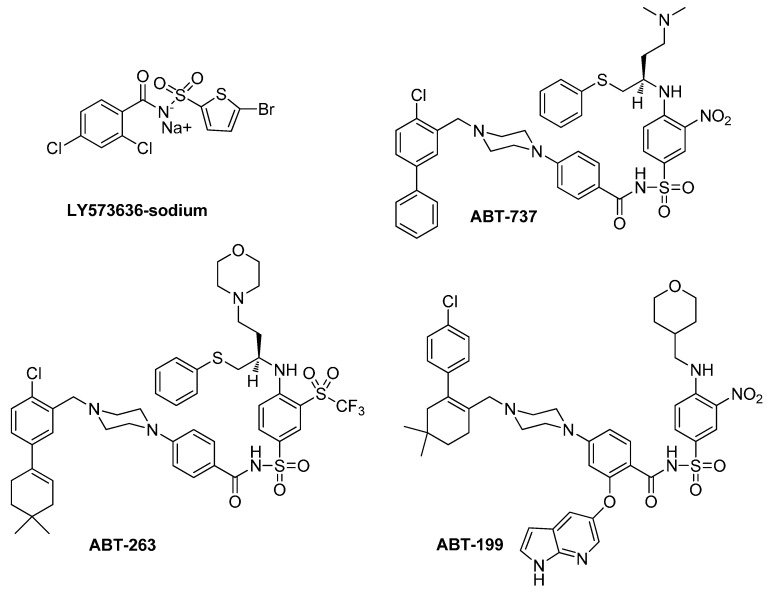
Clinically evaluated antitumor *N*-acylsulfonamides: LY573636-sodium (Tasisulam sodium) and ABT-737, ABT-263, ABT-199 (inhibitors targeting Bcl-2 family members).

Among the *N*-acylsulfonamides described in the literature so far, at least four have been clinically investigated as drug candidates with cytostatic activity against tumors: LY573636-sodium (tasisulam sodium) [10,11], ABT-737 [12], ABT-263 (navitoclax) [13,14] and ABT-199 [15] (Figure 1). LY573636-sodium acts an apoptosis inducer via the intrinsic pathway and exhibits antiangiogenesis activity, whereas ABT-737/263/199 are inhibitors of antiapoptotic Bcl-2 family members [10,11,12,13,14,15]. It should be noted that other acylsulfonamide analogues identified during the development of LY573636 have been reported to exhibit considerable antitumor activity, particularly against human colon, lung, prostate, ovarian, and breast xenografts growing in immunodeficient mice [16,17]. The current state of clinical trials concerning only LY573636-sodium or ABT-737/263/199, without combination with other drugs, is presented in Table 1 [18].

**Figure 2:**
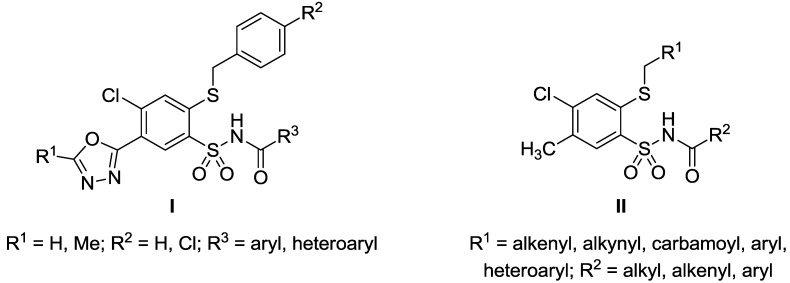
Structures of *N*-acylsulfonamides **I** and **II**.

Our systematic studies on aryl- and heteroarylsulfonamides have resulted in promising anticancer agents [19,20,21,22,23,24], among which we have reported some *N*-acylsulfonamides (**I**, Figure 2) with antiproliferative activity against a broad spectrum of cancer cell lines [23]. Searching for innovative low-molecular chemotherapeutics we designed a series of new *N*-acylsulfonamides **II** (Figure 2) with potential anticancer activity.

**Table 1:** Clinical Trial Information about LY573636-sodium, ABT-737, ABT-263 and ABT-199 [18].

Drug	Conditions	Study Start Date–Study Completion Date	Phase
LY573636-sodium	Brest cancer	2009‒2011	II
	Sarcoma, Soft Tissue	2007‒2010	II
	Ovarian Cancer	2007‒2012	II
	Non-Small-Cell Lung Cancer	2006‒2008	II
	Metastatic Melanoma	2006‒ongoing	II
ABT-737	Breast cancer	2013‒withdrawn	I/II
	Ovarian cancer	2010‒2013	II
ABT-263	Chronic Lymphoid Leukemia, Lymphoid Malignancies, NoN–Hodgkin’s Lymphoma, Follicular Lymphoma, Mantle Cell, Lymphoma, Peripheral T-cell Lymphoma	2006‒ongoing	II
	Chronic Lymphocytic, Leukemia, Lymphomas	2008‒2010	I
	Lymphoid Malignancies, Solid Tumors	2009‒2010	I
	Chronic Lymphocytic Leukemia	2007‒ongoing	II
	Chronic Lymphocytic Leukemia	2012‒2013	II
	Small Cell Lung Cancer	2007‒2010	I
	Small Cell Lung Carcinoma	2007‒2010	II
ABT-199	NoN–Hodgkin’s Lymphoma	2013‒ongoing	I
	Chronic Lymphocytic Leukemia	2013‒ongoing	I
	Relapsed/Refractory Multiple Myeloma	2012‒ongoing	I
	Acute Myelogenous/Myeloid Leukemia	2013‒2014	II
	Chronic Lymphocytic Leukemia	2014‒ongoing	II
	Chronic Lymphocytic Leukemia harboring the 17p Deletion, Cancer of the Blood and Bone Marrow	2013‒ongoing	II

To explain how structural features influence the biological activities the quantitative structure-activity relationship (QSAR) method was applied [25]. Metabolic stability studies, one of the parameters characterizing the pharmacokinetic properties of a molecule, was performed using pooled human liver microsomes and NADPH.

## 2. Results and Discussion

### 2.1. Chemistry

**Scheme 1:**
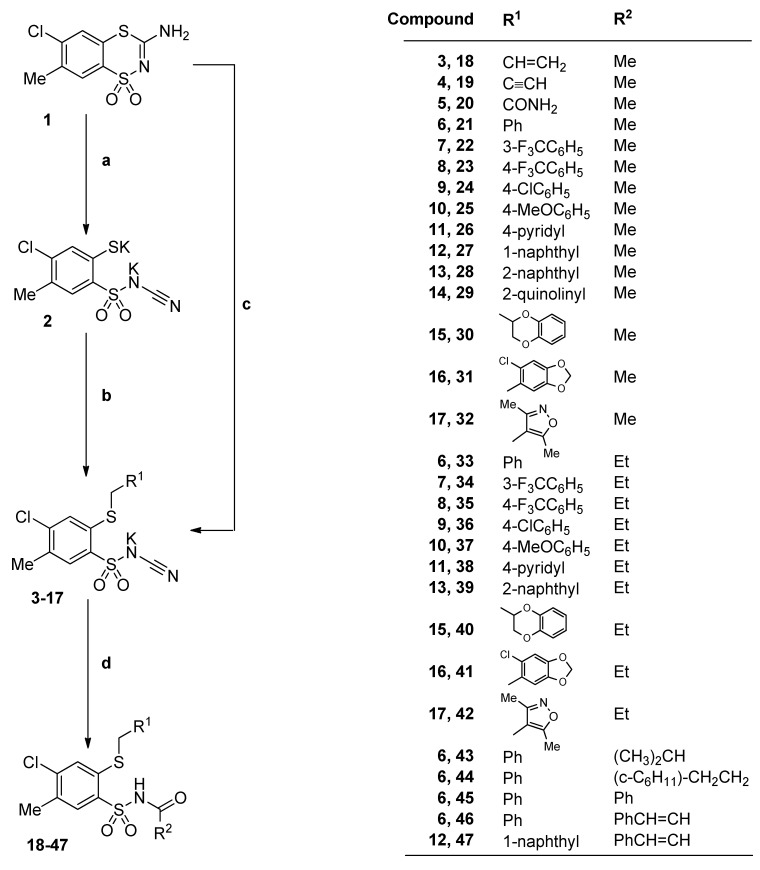
Synthesis of *N*-acyl-2-methylthio-4-chloro-5-methylbenzenesulfonamides **18**–**47**. *Reagents and conditions*: (**a**) K_2_CO_3_ excess, THF, reflux, 24 h; (**b**) R^1^CH_2_Cl/Br, water or methanol, r.t.; (**c**) PhCH_2_Cl, K_2_CO_3_ excess, THF, reflux, 24 h; (**d**) R^2^COOH, reflux (for **45**–**47** water or toluene).

The starting substrates, 3-aminobenzodithiazine **1**, dipotassium salt **2**, as well as potassium salts **3**‒**9**, were obtained according to the reported procedures for preparation of *N*-(benzenesulfonyl)cyanamide potassium salts [22,26,27]. Novel substrates **10**‒**17** were synthesized analogously by nucleophilic substitution of arylmethyl chloride with dipotassium salt **2** as was shown in Scheme 1. The final *N*-acylbenzenesulfonamides **18**–**47** were achieved in the one-step reaction of *N*-[4-chloro-5-methyl-2-(R¹-methylthio)benzenesulfonyl]cyanamide potassium salts with appropriate carboxylic acids under reflux. For solid acid reagents (*i.e.*, *trans*-cinnamic and benzoic acids) reactions were ran in water or toluene, whereas for liquid reactants (acetic, propionic, isobutyric, cyclohexylpropionic acids) syntheses were carried out without solvent.

**Scheme 2:**
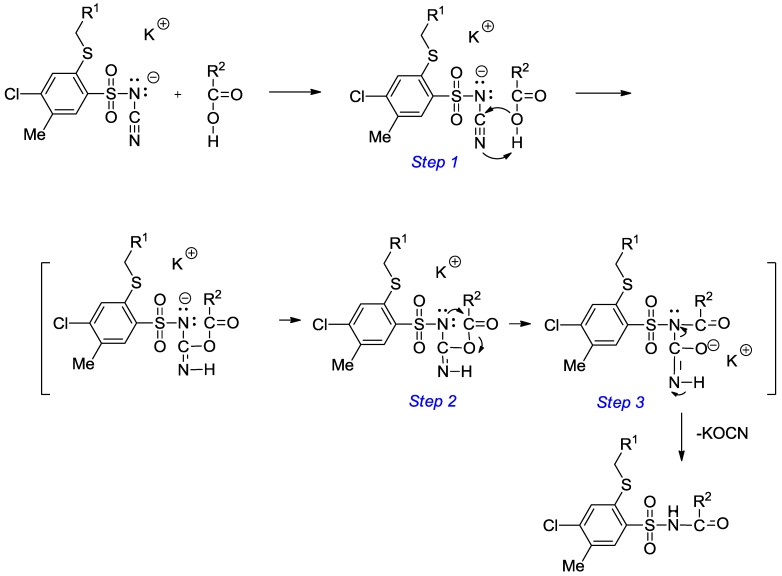
Proposed mechanism for the formation of *N*-acylbenzenesulfonamide derivatives **18**–**47**.

The proposed mechanism for the formation of final *N*-acylsulfonamides **18–47** is outlined in Scheme 2. The initial step we believe is the protonation of the nitrile nitrogen atom by the carboxylic acid with simultaneous acceptance of the lone pair of electrons from the carboxylate anion (*Step 1*). Then, the nitrogen atom attached to the sulfonyl group attacks the carbonyl carbon atom causing C–O bond cleavage of the carboxylate group (*Step 2*). In the final step the proton from the =N–H group is transferred to the deprotonated sulfonamide nitrogen atom with simultaneous potassium cyanate elimination (*Step 3*).

Many methods are known for the acylation of sulfonamides. *N*-Acylbeznenesulfonamides can be prepared, for instance, by the reaction of a sulfonamide with a carboxylic acid in the presence of coupling reagents such as carbodiimides (DCC, EDCI), in which the by-product must be removed from the reaction mixture [8,28]. The *N*-acylation of sulfonamides has also based on the reaction with mixed anhydrides [9], acyl chlorides [29] or *N*-acylbenzotriazoles [30] in basic reaction media as well as under acidic medium with carboxylic acids or anhydrides in the presence of Lewis acid as a catalyst [31].

In the present study we utilized a new and facile method for the synthesis of *N*-acylated sulfonamides using *N*-(benzenesulfonyl)cyanamide potassium salts which can be applied as a convenient alternative to the known methods. Basic advantages of described approach include low cost, absence of coupling reagents and harmful substances, and easy isolation of the products.

The structures of the final compounds **18**‒**47** were confirmed by IR, ^1^H-NMR and ^13^C-NMR spectroscopy. IR spectra showed the absence of the nitrile (C≡N) group and the presence of bands in the 1683‒1734 cm^−1^ range corresponding to the stretching vibration of the carbonyl group (C=O). This functional group was also identified in the ^13^C-NMR spectra where the *N*-acylbeznenesulfonamide moiety displays one carbon signal at 163.43‒180.90 ppm coming from the carbonyl carbon atom. The appearance of NH signals at 12.20‒12.82 ppm in the ^1^H-NMR spectra proved the presence of the amide group and also conformed the proposed structure of the final compounds. Moreover, X-ray analysis was done to confirm proposed structures on the representative compound **22**.

**Figure 3:**
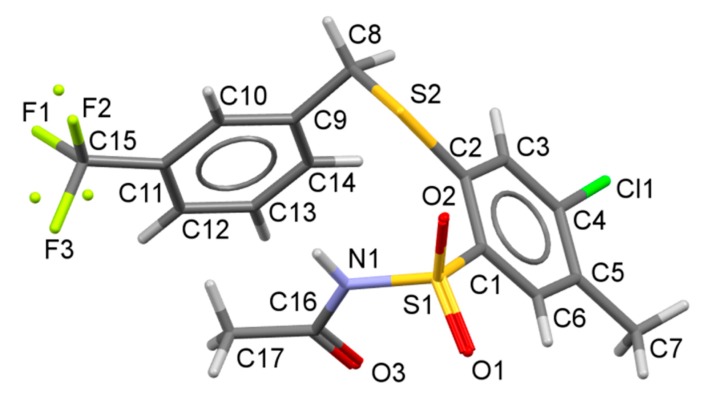
Atom numbering scheme for **22**, hydrogen atoms not labelled for clarity. Less populated fluorine atoms in disordered part of –CF_3_ shown as small, disconnected balls. Selected bond lengths [Å] and angles [°]: S1-O1 1.438(2), S1-O2 1.430(2), S1-N1 1.649(3), S1 C1 1.775(2), N1-C16 1.400(4), C16-O3 1.211(4), S2-C2 1.787(3), S2-C8 1.836(3); O1-S1-O2 119.3(1), O1-S1-N1 109.0(1), O2-S1-N1 105.0(1), C2-S2-C8 103.4(1), S1-N1-C16 124.6(2), N1-C16-C17 114.4(3), S2-C8-C9 114.1(2).

Details on data collection, structure solution and refinement are given in Table 2. Compound **22** crystallized in the monoclinic system, the space group *P*2_1_/*c*, with one molecule in the asymmetric unit. Atom numbering scheme was presented in Figure 3. The trifluoromethyl group was refined as disordered over two positions with probabilities 0.746(16)/0.254(16). The molecule is bent at the –CH_2_–S– bridge in such a way that the acyl group is placed over the Ph–CF_3_ ring.

**Table 2:** Crystal data and structure refinement details for **22**.

Empirical Formula	C_17_H_15_ClF_3_NO_3_S_2_
Formula weight	437.87
Temperature/K	293 (2)
Wavelength/Å	0.71073
Crystal system	Monoclinic
Space group	*P*2_1_/*c*
Unit cell dimensions	
*a*/Å	13.0071 (9)
*b*/Å	7.7047 (5)
*c*/Å	23.1637 (19)
α/°	90
β/°	120.288 (6)
γ/°	90
Volume/Å^3^	2004.5 (3)
*Z*	4
Density (calculated)/Mgm^−3^	1.451
Absorption coefficient/mm^−1^	0.44
*F*(000)	896
Crystal size/mm^3^	0.41 × 0.18 × 0.05
θ range for data collection/°	2.83–25.2
Index ranges	−15 ≤ h ≤ 15, −9 ≤ k ≤ 9, −26 ≤ l ≤ 27
Reflections collected	12,224
Independent reflections	3605 (*R*_int_ = 0.031)
Completeness to θ = 50°	99.9%
Absorption correction	Multi-scan
Refinement method	Full-matrix least-squares on *F*^2^
Data/restraints/parameters	3605/16/278
Goodness of fit on *F*^2^	1.024
Final *R* indices [I > 2σ(I)]	*R*_1_ = 0.0499, *wR*_2_ = 0.1222
*R* indices (all data)	*R*_1_ = 0.0635, *wR*_2_ = 0.1364
Largest diff. peak and hole/eÅ^−3^	0.32, −0.30

**Figure 4:**
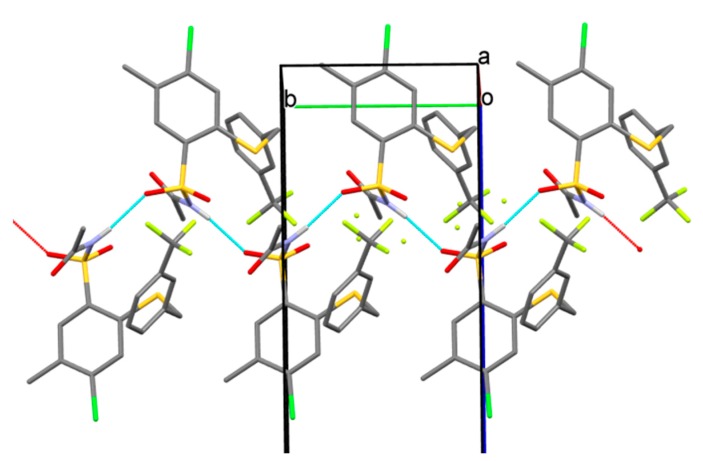
Chain of molecules linked by hydrogen bonding and spreading along the screw 2_1_ axis, parallel to the **b** axis (½ y ¼).

The valence angle at S2, *ca*. 103° is typical for a thioether group. Notably, the S–C bond length to aliphatic carbon is longer than the bond to aromatic ring, which may indicate to some mesomeric effect. Since the sulfonamide group is acylated only one N–H hydrogen-bond donor is available. The intermolecular hydrogen bonding, of the N–H…O type, forms infinite chains (graph set symbol C(4), [32]) spreading along the **b** axis (Figure 4, Table 3). The chain propagates exactly along the crystal screw axis 2_1_ so it is described by the 𝓅112_1_ (R9) rod group symmetry [33].

**Table 3:** Hydrogen bond geometry (Å, °).

*D*–H···*A*	*D*–H	H···*A*	*D*···*A*	*D*–H···*A*
N1–H1N···O1 ^i^	0.865 (18)	2.028 (19)	2.887 (3)	172 (3)

Symmetry code: (i) −x + 1, y + 1/2, −z + 3/2.

**Figure 5:**
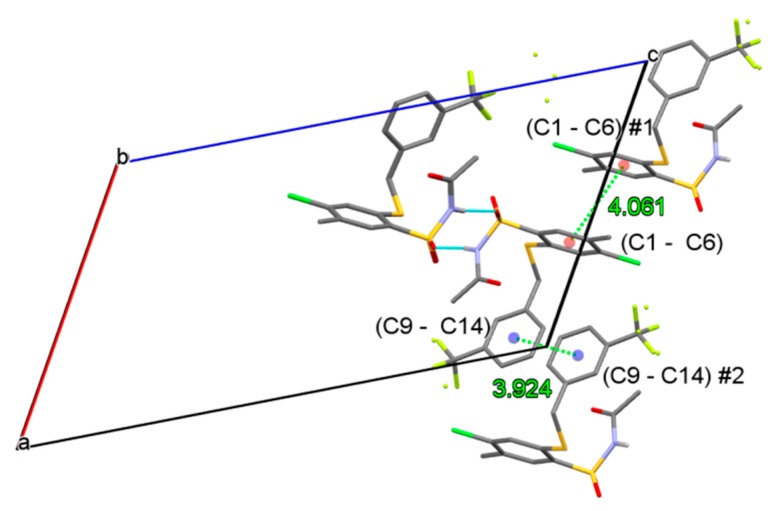
Ring stacking interactions, operating among the hydrogen-bonded chains. Symmetry codes #1: (1 − x, 1 − y, 2 − z); #2: (2 − x, 2 − y, 2 − z). Only few, selected molecules are shown.

Oxygen atom O(1), involved in the interaction, is a little more distant from S(1) than the other oxygen atom O3 (1.438(2) *vs*. 1.430(2) Å), which is expected and confirms the hydrogen bonding. The chains are packed in the crystal in such a way that allows ring stacking interactions between ring C1-C6 and its symmetry equivalent related by inversion centre at (½ ½ 1) and ring C9-C14 and its equivalent related by inversion centre at (1 1 1) (see Figure 5).

### 2.2. Cytotoxic Activity

Compounds **19**‒**47** were evaluated *in vitro* for their effects on the viability of three human cancer cell lines: MCF-7 (breast cancer), HCT-116 (colon cancer) and HeLa (cervical cancer). Cytotoxic evaluations were performed in a 5-dose assay and data is reported as the percent growth of the treated cells. Results in Table 4 show the growth percent (GP) at two representative doses, *i.e.*, 10 and 100 µM. The most active compound **27** belonged to the 1-naphthyl series (R^1^ = 1-naphthyl) and showed outstanding anticancer activity at low and high concentrations against all tested cell lines, especially towards to the HeLa cell line (GP = 7% at 100 µM, GP = 22% at 10 µM).

**Table 4:** Cytotoxicity of compounds **19**‒**47** toward human cancer cell lines.

Compd.	GP (%) ^a^					
	MCF-7		HeLa		HCT-116	
	10 µM	100 µM	10 µM	100 µM	10 µM	100 µM
**19**	88 ± 2	87 ± 2	100 ± 2	76 ± 2	100 ± 2	92 ± 3
**20**	88 ± 3	82 ± 2	90 ± 4	71 ± 2	101 ± 2	93 ± 2
**21**	94 ± 3	84 ± 3	97 ± 2	92 ± 1	93 ± 6	84 ± 6
**22**	91 ± 4	77 ± 7	91 ± 6	65 ± 7	86 ± 7	68 ± 5
**23**	93 ± 2	77 ± 2	87 ± 4	89 ± 6	86 ± 6	75 ± 3
**24**	93 ± 5	91 ± 5	97 ± 1	91 ± 1	91 ± 6	76 ± 4
**25**	97 ± 5	87 ± 5	94 ± 3	79 ± 6	84 ± 5	81 ± 6
**26**	97 ± 2	93 ± 6	98 ± 2	91 ± 1	85 ± 6	92 ± 5
**27**	82 ± 5	44 ± 5	22 ± 2	7 ± 0.6	63 ± 4	15 ± 1
**28**	96 ± 4	82 ± 4	100 ± 2	92 ± 3	86 ± 8	71 ± 2
**29**	95 ± 4	81 ± 4	87 ± 2	87 ± 3	74 ± 0.4	79 ± 1
**30**	93 ± 7	81 ± 1	95 ± 5	91 ± 1	85 ± 4	82 ± 2
**31**	93 ± 6	85 ± 6	95 ± 2	95 ± 4	86 ± 7	70 ± 4
**32**	96 ± 2	89 ± 3	100 ± 2	92 ± 4	89 ± 7	86 ± 5
**33**	93 ± 8	71 ± 3	100 ± 2	93 ± 3	86 ± 5	83 ± 5
**34**	86 ± 7	62 ± 0.4	96 ± 4	95 ± 5	92 ± 6	69 ± 6
**35**	89 ± 7	82 ± 4	98 ± 5	92 ± 4	84 ± 7	76 ± 2
**36**	93 ± 4	73 ± 0.1	101 ± 0.1	95 ± 1	93 ± 7	72 ± 7
**37**	95 ± 3	72 ± 0.5	99 ± 1	91 ± 4	91 ± 5	85 ± 5
**38**	95 ± 3	81 ± 0.8	98 ± 4	95 ± 2	99 ± 7	83 ± 4
**39**	94 ± 9	65 ± 5	99 ± 1	72 ± 5	94 ± 2	65 ± 2
**40**	93 ± 4	86 ± 8	99 ± 0.5	98 ± 0.5	96 ± 4	89 ± 2
**41**	100 ± 1	61 ± 0.1	100 ± 1	86 ± 3	92 ± 3	69 ± 2
**42**	89 ± 3	79 ± 2	98 ± 2	99 ± 0.2	92 ± 1	90 ± 4
**43**	95 ±5	81 ± 6	101 ± 3	95 ± 5	89 ± 5	83 ± 5
**44**	92 ± 6	66 ± 4	98 ± 1	43 ± 3	93 ± 4	40 ± 4
**45**	100 ± 3	76 ± 1	97 ± 3	18 ± 1	92 ± 7	61 ± 2
**46**	96 ± 5	79 ± 4	100 ± 2	80 ± 3	89 ± 6	84 ± 2
**47**	92 ± 4	46 ± 5	100 ± 2	9 ± 0.8	91 ± 4	18 ± 2

^a^ Growth percent (GP) was measured at concentrations 1, 10, 25, 50 and 100 µM. Two representative doses were shown, *i.e.*, 10 µM and 100 µM. Values are expressed as the mean ± SD of at least three independent experiments.

It was noted that changing R^1^ = 1-naphthyl in active compound **27** to the corresponding 2-naphthyl isomer (compound **28**) markedly decreased this activity. Another 1-naphthyl derivative **47** also stood out from the other compounds and demonstrated dilution-dependent good cytotoxic activity toward the three cell lines (9% ≤ GP ≤ 46% at 100 µM). The significance of the 1-naphthyl substituent for the anticancer activity emphasize the fact that, replacement of this group (R^1^ = 1-naphthyl, **47**) by phenyl reduced activity of compound (R^1^ = phenyl, **46**, 79% ≤ GP ≤ 84% at 100 µM).

We found that among *N*-acylsulfonamides with R^1^ = phenyl the presence of R^2^ = phenyl group provided good cytotoxicity (compound **45**) toward the HeLa cells (GP = 18%) and replacement of phenyl substituent in this compound by an aliphatic group, *i.e.*, **21** (R^2^ = Me), **33** (R^2^ = Et), **43** (R^2^ = *i*Pr) or **44** (R^2^ = CH_2_CH_2_(*c–C*_6_H_11_)) caused decreases in the activity of **44** (GP = 43%) or the loss of activity (**21**, **33**, **43**) at a concentration of 100 µM.

For the most active compounds **27**, **44**, **45** and **47**, the concentration required for 50% inhibition of cell viability IC_50_ was calculated and compared with the reference drug cisplatin, and the results are given in Table 5.

**Table 5:** IC_50_ values for compounds **27**, **44**, **45** and **47**.

Compd.	IC_50_ (µM)		
	MCF-7	HeLa	HCT-116
**27**	30 ± 1.2	6 ± 0.2	14 ± 0.5
**44**	170 ± 12	95 ± 3	91 ± 3
**45**	200 ± 16	75 ± 4	135 ± 7
**47**	96 ± 5	43 ± 2	77 ± 3
Cisplatin	3.0 ± 0.1	3.8 ± 0.2	2.2 ± 0.1

Despite our observations concerning mentioned above correlations between structure and activity, we decided to apply more objective methods of establishing the structure-activity relationships, such as QSAR methodology.

### 2.3. Quantitative Structure-Activity Relationships (QSARs) of Cytotoxic Activity

The exploration of the relationship between structure and cytotoxic activity can be carried out by QSAR study. Recently, pharmacophore-based approaches are considered as the most advanced and successful, however if the molecular target of a group of compounds is not known, traditional regression-based techniques can be applied.

**Figure 6:**
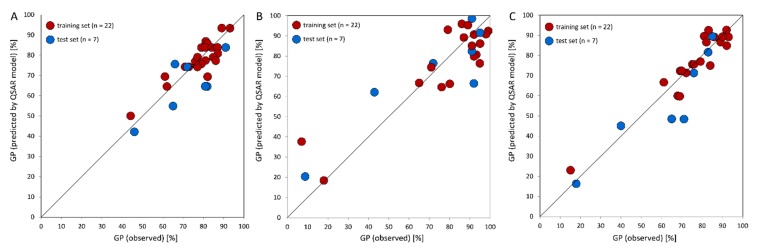
The comparison between growth percent (GP) values observed experimentally and predicted by QSAR models for: MCF-7 (**A**); HeLa (**B**) and HCT-116 (**C**) cell lines. Training and test compounds are shown by red and blue circles, respectively.

Multiple linear regression technique (MLR) along with stepwise algorithm usually leads to a statistically significant equation, that can be interpret based on a values of selected descriptors. Many pitfalls and mistakes can be done during development of a QSAR model. Among them, overfitting is one of the most common, and may results in false interpretation of a QSAR model. The second crucial issue is to develop a model in a way that enables to interpret quantitative relationship in the view of chemical structure. In order to achieve informative and statistically significant relationship we carefully designed several QSAR models using three-dimensional structures developed in Gaussian software and activity data taken from three distinct cell lines. Only selected descriptors available in DRAGON package were used (Table 6). MLR gave three statistically significant equations, one for each cell line (Table 7). The comparison between experimentally observed and predicted GP values is visualized in Figure 6.

For the MCF-7 cell line, three descriptors were pointed out as significant for compounds’ activity. First of all the number of ring systems (NRS), with its positive coefficient shows that additional rings generally decrease the biological activity. CATS2D_06_DL shows a preferable topological distance (6 bonds) between lipophilic and H-bond donor centers and F07[C–C] supplements its performance indicating a preferred distance between C atoms (Figure S1, see Supplementary Data). QSAR model for HeLa cell line contains four molecular descriptors. It shows two beneficial features: frequency of C–S atom pair at topological distance 8 and positive charge at the S atom of the sulfonamide moiety. Also two features are shown to decrease activity: topological distance between H-bond donor and acceptor (seven bonds) and the longest geometric distance from the atom to any other atom in the molecule (molecule width) (Figure S1, see Supplementary Data).

**Table 6:** Molecular descriptors (ring, topological, charge descriptors and atom-centred fragments) calculated by Dragon (Talete, Milano, Italy, version 6.0).

Compd.	Ring	Topological								Atom-Centred	Charge
	NRS	CATS2D_06_DL	CATS2D_07_DA	CATS2D_03_LL	F07 [C–C]	F08 [C–S]	F05 [C–S]	B10 [C–S]	ECC	C-024	qpmax
**19**	1	2	0	6	6	0	3	0	135	2	2.849
**20**	1	2	3	5	5	0	3	0	144	2	2.861
**21**	2	2	0	10	13	1	4	0	201	7	2.843
**22**	2	2	0	10	15	2	5	0	263	6	2.844
**23**	2	2	0	10	14	1	4	0	283	6	2.844
**24**	2	2	0	11	13	1	4	0	228	6	2.844
**25**	2	2	0	9	13	1	4	1	257	6	2.843
**26**	2	2	0	7	11	0	3	0	201	4	2.844
**27**	2	2	0	19	20	3	6	0	260	9	2.845
**28**	2	2	0	19	17	2	5	1	280	9	2.845
**29**	2	1	1	11	16	2	5	1	280	8	2.844
**30**	2	1	1	6	14	2	5	1	280	6	2.845
**31**	2	2	0	8	14	1	4	0	261	4	2.845
**32**	2	2	0	5	11	0	3	0	195	2	2.846
**33**	2	2	0	10	15	1	4	0	221	7	2.844
**34**	2	2	0	10	17	2	5	0	288	6	2.843
**35**	2	2	0	10	16	1	4	0	308	6	2.844
**36**	2	2	0	11	15	1	4	0	250	6	2.843
**37**	2	2	0	9	15	1	4	1	280	6	2.843
**38**	2	2	0	7	13	0	3	0	221	4	2.843
**39**	2	2	0	19	19	2	5	1	305	9	2.844
**40**	2	1	1	6	16	2	5	1	305	6	2.845
**41**	2	2	0	8	16	1	4	0	286	4	2.844
**42**	2	2	0	5	13	0	3	0	219	2	2.847
**43**	2	2	0	10	17	1	4	0	233	7	2.842
**44**	3	4	0	17	19	3	5	1	364	7	2.845
**45**	3	2	0	13	22	3	6	0	297	12	2.852
**46**	3	4	0	17	19	3	5	1	364	12	2.844
**47**	3	4	0	26	26	5	7	1	442	14	2.848

NRS—number of ring systems; CATS2D_06_DL—CATS2D Donor-Lipophilic at lag 06; F07[C–C]—frequency of C–C at topological distance 7; F08[C–S]—frequency of C–S at topological distance 8; qpmax—maximum positive charge (at sulfonamide S atom); CATS2D_07_DA—CATS2D Donor-Acceptor at lag 07; ECC—eccentricity (the longest geometric distance from the atom to any other atom in the molecule); CATS2D_03_LL—CATS2D Lipophilic-Lipophilic at lag 03; F05[C–S]—frequency of C–S at topological distance 5; C-024—number of fragments C_(Ar)_–CH–C_(Ar)_; B10[C–S]—presence/absence of C–S at topological distance 10.

**Table 7:** Summary of the QSAR equations developed for compounds under the study.

**Cell Line: MCF-7**
GP = 89.07 ± 6.24 + 36.51 ± 6.05**NRS** − 7.88 ± 2.51 **CATS2D_06_DL** − 4.81 ± 0.63 **F07[C–C]**
R^2^ = 0.77; Q^2^ = 0.59; F = 19.77; *p* = 6.3 × 10^−6^
*p*_NRS_ = 0.000011; *p*_CATS22_06_DL_ = 0.005632; *p*_F07[C–C]_ = 0.000001
**Cell Line: HeLa**
GP = 11409.30 ± 3252.32 − 24.10 ± 4.46 **F08[C–S]** − 3993.18 ± 1140.25 **qpmax** + 18.60 ± 5.92 **CATS2D_07_DA** + 0.24 ± 0.09 **ECC**
R^2^ = 0.76; Q^2^ = 0.81; F = 13,77; *p* = 3.5 × 10^−5^
*p*_F08[C–S]_ = 0.000048; *p*_qpmax_ = 0.002732; *p*_CATS2D_07_DA_ = 0.005922; *p*_ECC_ = 0.020743
**Cell Line: HCT-116**
GP = 146.53 ± 7.22 − 4.32 ± 0.76 **CATS2D_03_LL** − 15.90 ± 2.51 **F05[C–S]** + 5.98 ± 1.20 **C-024** + 9.59 ± 3.72 **B10[C–S]**
R^2^ = 0.89; Q^2^ = 0.83; F = 36.89; *p* = 6.08 × 10^−8^
p_CATS2D_03_LL_ = 0.000026; *p*_F05[C–S]_ = 0.000007; *p*_C-024_ = 0.000114; *p*_B10[C–S]_ = 0.019460

R^2^—squared correlation coefficient for training set; Q^2^—squared correlation coefficient for test set; F—Fisher’s test; *p*—*p*-value for Fisher’s test for the whole equation and particular descriptors.

The HCT-116 QSAR model showed the best performance (R^2^ = 0.89; Q^2^ = 0.83) for both the training and test sets of compounds (Figure 6C). This points out the increase of biological activity with the increased number of lipophilic centers at topological distance 3 and the frequency of C–S atom pair at distance 5. On the other hand, C_(Ar)_–CH–C_(Ar)_ fragment as well as presence of C–S at topological distance 10 were showed to be unfavorable (Figure S1, see Supplementary Data).

### 2.4. Metabolic Stability

Most small-molecule anticancer drugs are cytotoxic agents with a narrow therapeutic window. Moreover their activity can be influenced by significant inter- and intra-subject variability, which can lead to life-threatening consequences. The probability to develop a successful drug can be increased by assessments of drugs’ pharmacokinetic characteristics, *i.e.*, absorption, distribution, metabolism and excretion properties, as early as possible during the preclinical stage. Among them metabolism is very important because it involves conversion of a drug candidate into a set of metabolites that differ in both activity and toxicity, leading to complex interactions within the human body. Metabolic stability can be assessed by incubation of a potent drug in a presence of liver microsomes and NADPH to give an insight to metabolic properties [34,35,36].

Selected compounds were submitted to *in vitro* metabolic stability study in the presence of human liver microsomes and NADPH (Table 8). Determination of metabolic half-time (t_1/2_) was performed by LC-MS analysis. Metabolic stability in this set of compounds, expressed as t_1/2_, depends on a type of substituent in R^1^ and R^2^ positions. The most liable derivatives were **42** and **44** with t_1/2_ below 5 min. Among the most potent compounds **27** and **47**, the first one was more stable with t_1/2_ 24.23 min., which is a moderate value in a studied set of compounds. Two compounds substituted with ethynyl (compound **19**) and amide (compound **20**) moieties were characterized by a high metabolic stability, exceeding 40 min.

**Table 8:** Results of metabolic stability study.

Compd.	*In Vitro* Metabolic Half-Life t_1/2_ (min)
**19**	41.50
**20**	>>60
**21**	14.29
**27**	24.23
**28**	68.61
**30**	17.67
**31**	6.00
**32**	12.30
**42**	<5
**44**	<5
**45**	21.06
**46**	13.20
**47**	20.20

**Figure 7:**
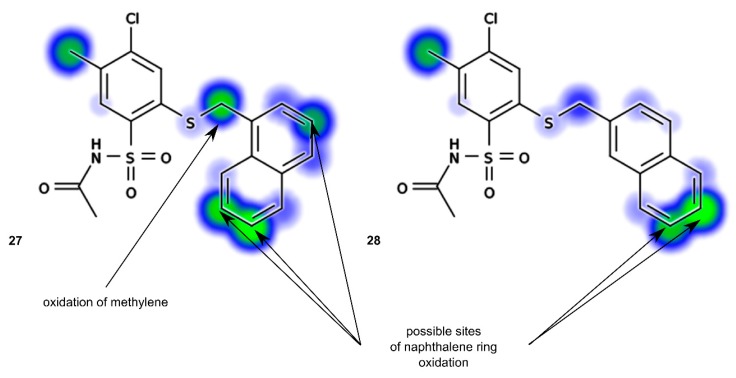
Sites of metabolism predicted for **27** and **28** by XenoSite software [37]. Green color indicates more vulnerability to biotransformation than blue. Some significant differences are additionally pointed out.

Interestingly, big difference in t_1/2_ value was observed between **28** and **27**. The increased metabolic stability results from the shift of naphthyl substituent from 1- to 2-naphthyl position. In order to describe this phenomenon in more detailed way, we decided to use *in silico* approach. Recently, Zaretzki and co-workers developed on-line accessible tool for accurate prediction of xenobiotic sites of metabolism, called XenoSite [37]. Among available models, one is able to predict stability measured in a presence of human liver microsomes. Taking into account *in silico* results illustrated in Figure 7, the relation with experimental determination of metabolic stability is clear. Compound **27** has two more sites vulnerable for metabolic biotransformation than **28**, one in naphthalene ring and in methylene linker.

Among the most potent compounds, **44** bears an ethylcyclohexyl moiety, which is probably the reason for its low stability. This indicates that this substituent should be excluded from further synthesis in this series of derivatives. On the other hand, although 2-naphtyl substitution is preferable for metabolic stability in comparison with a 1-naphtyl moiety, the high biological response observed for the latter one predisposes it to be a lead compound, as it shows a good balance between stability and cytotoxic properties.

## 3. Experimental Section

### 3.1. General Information

Melting points were uncorrected and measured using a Boetius PHMK apparatus (Veb Analytic, Dresden, Germany). IR spectra were measured on a Satellite FTIR spectrometer (Thermo Mattson, Madison, WI, USA) using KBr pellets; the absorption range was 400–4000 cm^−1^. ^1^H-NMR and ^13^C-NMR spectra were recorded on a Gemini 200 apparatus or a Unity Plus 500 apparatus (Varian, Palo Alto, CA, USA). Chemical shifts are expressed at δ values relative to Me_4_Si (TMS) as an internal standard. Elemental analyses were determined on a 2400 Series II CHN Elemental Analyzer (Perkin Elmer, Shelton, CT, USA) and were in agreement with the theoretical values within a ±0.4% range. Thin-layer chromatography (TLC) was performed on Kieselgel 60 F254 plates (Merck, Darmstadt, Germany) and visualized by UV. Commercially unavailable substrates were obtained according to the following methods described previously: 3-amino-6-chloro-7-methyl-1,1-dioxo-1,4,2-benzodithiazine (**1**) [26] *N*-(4-chloro-2-mercapto-5-methylbenzenesulfonyl)cyanamide dipotassium salt (**2**) [26], *N*-(2-allylthio-4-chloro-5-methylbenzenesulfonyl)cyanamide potassium salt (**3**) [27], *N*-[4-chloro-5-methyl-2-(prop-2-ynylthio)benzenesulfonyl]cyanamide potassium salt (**4**) [27], *N*-(2-carbamoylthio-4-chloro-5-methylbenzenesulfonyl)cyanamide potassium salt (**5**) [27], *N*-(2-benzylthio-4-chloro-5-methyl-benzenesulfonyl)cyanamide potassium salt (**6**) [26], *N*-[4-chloro-5-methyl-2-(3-trifluoromethyl-benzylthio)benzenesulfonyl]cyanamide potassium salt (**7**) [22], *N*-[4-chloro-5-methyl-2-(4-trifluoro-methylbenzylthio)benzenesulfonyl]cyanamide potassium salt (**8**) [22], *N*-[4-chloro-2-(4-chloro-benzylthio)-5-methylbenzenesulfonyl]cyanamide potassium salt (**9**) [38], *N*-[4-chloro-5-methyl-2-(naphthalen-1-ylmethylthio)benzenesulfonyl]cyanamide potassium salt (**12**) [22], *N*-[4-chloro-2-(2,3-dihydrobenzo[*b*][1,4]dioxin-2-ylmethylthio)-5-methylbenzenesulfonyl]cyanamide potassium salt (**15**) [22], and *N*-[4-chloro-2-(6-chlorobenzo[*d*][1,3]dioxol-5-ylmethylthio)-5-methylbenzenesulfonyl] cyanamide potassium salt (**16**) [39].

### 3.2. Chemistry

#### 3.2.1. Procedures for Preparation of Cyanamide Potassium Salts **10**–**11**, **13**, **14**, **17**

*N-[4-Chloro-2-(4-methoxybenzylthio)-5-methylbenzenesulfonyl]cyanamide Potassium Salt* (**10**). To a solution of **2** (2.032 g, 6 mmol) in water (20 mL) 4-methoxybenzyl chloride (0.916 mL, 6.6 mmol) was added and the resulting mixture was stirred at 0 °C for 4 h. A white solid was filtered off and crystallized from ethanol, giving the title compound **10** (2.400 g, 95%): mp 127‒130 °C; IR (KBr) 2953, 2833 (CH_3_, CH_2_), 2176 (C≡N), 1343, 1176 (SO_2_) cm^−1^; ^1^H-NMR (500 MHz, DMSO-*d*_6_) δ 2.29 (s, 3H, CH_3_), 3.72 (s, 3H, OCH_3_), 4.19 (s, 2H, SCH_2_), 6.88 (d, *J* = 8.3 Hz, 2H, arom.), 7.34 (d, *J* = 8.3 Hz, 2H, arom.), 7.37 (s, 1H, H-3), 7.72 (s, 1H, H-6) ppm; ^13^C-NMR (125 MHz, DMSO-*d*_6_) δ 19.66, 36.20, 55.76, 114.54, 117.94, 127.35, 128.58, 131.00, 131.19, 131.61, 136.37, 136.56, 140.86, 159.11 ppm; anal. C 45.26, H 3.20, N 6.25% calcd for C_16_H_14_ClKN_2_O_3_S_2_, C 45.65, H 3.35, N 6.65%.

*N-[4-Chloro-5-methyl-2-(pyridin-4-ylmethylthio)benzenesulfonyl]cyanamide Potassium Salt* (**11**). The mixture of 4-(chloromethyl)pyridine (1.548 g, 9.45 mmol) and K_2_CO_3_ (1.242 g, 9.00 mmol) in methanol (45 mL) was stirred at room temperature for 10 min, followed by addition of dipotassium salt **2** (3.050 g, 9.00 mmol). The resulting slurry was stirred at 60 °C for 1 h. After completion of reaction ethyl ether (45 mL) was added and a precipitated solid (4.410 g) was filtered off. The pure product was extracted with boiling ethanol (180 mL) giving the title compound **11** (2.982 g, 76%): mp 162‒164 °C; IR (KBr) 2924, 2853 (CH_3_, CH_2_), 2175 (C≡N), 1342, 1140 (SO_2_) cm^−1^; ^1^H-NMR (500 MHz, DMSO-*d*_6_) δ 2.27 (s, 3H, CH_3_), 4.32 (s, 2H, SCH_2_), 7.35 (s, 1H, H-3), 7.44 (d, *J* = 5.9 Hz, 2H, pyridine), 7.73 (s, 1H, H-6), 8.48 (d, *J* = 5.9 Hz, 2H, pyridine); ^13^C-NMR (125 MHz, DMSO-*d*_6_) δ 19.66, 35.35, 117.88, 124.79, 127.89, 131.29, 132.30, 135.04, 136.40, 141.47, 146.71, 150.32 ppm; anal. C 42.70, H 2.75, N 10.60% calcd for C_14_H_11_ClKN_3_O_2_S_2_, C 42.90, H 2.83, N 10.72%.

*N-[4-Chloro-5-methyl-2-(naphthalen-2-ylmethylthio)benzenesulfonyl]cyanamide Potassium Salt* (**13**). To a solution of **2** (0.700 g, 2.06 mmol) in water (6 mL) 2-(chloromethyl)naphthalene (0.457 g, 2.06 mmol) was added and the resulting mixture was stirred at room temperature for 12 h. The solid was filtered off and crystallized from ethanol to afford **13** (0.753 g, 83%): mp 207‒209 °C; IR (KBr) 2921 (CH_3_, CH_2_), 2175 (C≡N), 1342, 1140 ( SO_2_) cm^−1^; ^1^H-NMR (500 MHz, DMSO-*d*_6_) δ 2.27 (s, 3H, CH_3_), 4.44 (s, 2H, SCH_2_), 7.45 (s, 1H, arom.), 7.47‒7.50 (m, 2H, arom.), 7.59 (d, 1H, arom.), 7.73 (s, 1H, H-3), 7.84‒7.89 (m, 3H, arom.), 7.95 (s, 1H, H-6); ^13^C-NMR (125 MHz, DMSO-*d*_6_) δ 19.66, 36.94, 117.96, 126.61, 126.97, 127.64,128.07, 128.23, 128.28, 128.68, 131.23, 131.86, 132.82, 133.53, 134.69, 136.10, 136.38, 141.13 ppm; anal. C 51.45, H 3.11, N 6.12% calcd for C_19_H_14_ClKN_2_O_2_S_2_, C 51.75, H 3.20, N 6.35%.

*N-[4-Chloro-5-methyl-2-(quinolin-2-ylmethylthio)benzenesulfonyl]cyanamide Potassium Salt* (**14**). The mixture of **2** (0.505 g, 1.50 mmol), 2-chloromethylquinoline (0.320 g, 1.50 mmol), K_2_CO_3_ (0.104 g, 0.75 mmol) in methanol (10 mL) was stirred at 50 °C for 2 h. The reaction mixture was evaporated under reduced pressure to dryness, then treated with water (10 mL) and stirred using an ultrasonic bath for 3 min. The obtained solid was filtered off and crystallized from ethanol to afford **14** (0.359 g, 54%): mp 207‒210 °C; IR (KBr) 2924 (CH_3_CH_2_), 2176, (C≡N), 1344, 1143 (SO_2_) cm^−1^; ^1^H-NMR (200 MHz, DMSO-*d*_6_) δ 2.26 (s, 3H, CH_3_), 4.54 (s, 2H, SCH_2_), 7.50‒7.82 (m, 5H, H-6, H-3 and quinol.), 7.85‒8.20 (m, 2H, quinol.), 8.35 (d, 1H, quinol.); ^13^C-NMR (125 MHz, DMSO-*d*_6_) δ 19.56, 39.27, 117.88, 122.24, 127.15, 127.39, 127.55, 128.52, 129.03, 130.47, 131.17, 131.77, 135.89, 136.39, 137.63, 140.89, 147.38, 158.54 ppm; anal. C 47.00, H 2.80, N 9.00% calcd for C_18_H_13_ClKN_3_O_2_S_2_, C 47.20, H 2.86, N 9.17%.

*N-[4-Chloro-2-(3,5-dimethylisoxazol-4-ylmethylthio)-5-methylbenzenesulfonyl]cyanamide Potassium Salt* (**17**). To the mixture of **2** (0.500 g, 1.48 mmol) in water (4 mL) chloromethyl-3,5-dimethylisoxazol (0.183 mL, 1.48 mmol) was added and stirred at room temperature for 1.5 h, then at 100 °C for 6 h. After standing overnight at room temperature a precipitated solid was filtered off and crystallized from ethanol to afford the title compound **17** (0.700 g, 58%): mp 205‒206 °C; IR (KBr) 2923, 2853 (CH₃, CH_2_), 2181 (C≡N), 1274, 1141 (SO_2_) cm^−1^; ^1^H-NMR (200 MHz, DMSO-*d*_6_) δ 2.25 (s, 3H, CH_3_), 2.33 (s, 3H, CH_3_), 2.34 (s, 3H, CH_3_), 4.09 (s, 2H, SCH_2_), 7.44 (s, 1H, H-3), 7.75 (s, 1H, H-6) ppm; ^13^C-NMR (50 MHz, DMSO-*d*_6_) δ 9.99, 10.85, 19.29, 24.76, 109.41, 117.46, 129.13, 130.77, 132.30, 134.51, 135.79, 141.94, 159.86, 166.79 ppm; anal. C 40.72, H 3.15, N 10.05% calcd for C_14_H_13_ClKN_3_O_3_S_2_, C 41.02, H 3.20, N 10.25%.

#### 3.2.2. Procedure for the Preparation of *N*-Acylbenzenesulfonamides **18**‒**44**

A solution of monopotassium salts **3**‒**17** (1 mmol) in an appropriate carboxylic acid (40 mmol) was refluxed for 1‒17 h and left overnight at room temperature. The precipitate was filtered off, dried and crystallized from ethanol.

*N-(2-Allylthio-4-chloro-5-methylphenylsulfonyl)acetamide* (**18**). Starting from **3** (0.341 g) and acetic acid (2.5 mL) the title compound **18** (0.230 g, 72%) was obtained after 15 h at reflux: mp 138‒140 °C; IR (KBr) 3334, 3241 (NH), 2923 (CH_3_, CH_2_), 1686 (C=O), 1352, 1160 (SO_2_) cm^−1^; ^1^H-NMR (500 MHz, DMSO-*d*_6_) δ 1.95 (s, 3H, CH_3_CO), 2.40 (s, 3H, CH_3_), 3.86 (d, 2H, SCH_2_), 4.94 (dd, 2H, =CH_2_), 5.77–5.84 (m, 1H, HC=), 7.62 (s, 1H, H-3), 7.95 (s, 1H, H-6), 12.34 (s, 1H, NH) ppm; anal. C 44.75, H 4.31, N 4.61% calcd for C_12_H_14_ClNO_3_S_2_, C 45.06, H 4.41, N 4.38%.

*N-[4-Chloro-5-methyl-2-(prop-2-ynylthio)phenylsulfonyl]acetamide* (**19**). Starting from **4** (0.339 g) and acetic acid (2.5 mL) the title compound **19** was obtained (0.113 g, 35%) after for 17 h at reflux: mp 148‒150 °C; IR (KBr) 3448, 3298 (NH), 3086 (C≡CH), 2981, 2876 (CH_3_, CH_2_), 1692 (C=O), 1472 (N–H), 1353, 1165 (SO_2_) cm^−1^; ^1^H-NMR (500 MHz, DMSO-*d*_6_) δ 1.91 (s, 3H, CH_3_CO), 2.36 (s, 3H, CH_3_), 3.20 (s, 1H, C≡CH), 4.06 (s, 2H, SCH_2_), 7.68 (s, 1H, H-3), 7.92 (s, 1H, H-6), 12.34 (s, 1H, NH); ^13^C-NMR (125 MHz, DMSO-*d*_6_) δ 19.69, 21.00, 23.90, 74.95, 80.03, 129.01, 133.66, 134.19, 135.11, 135.82, 139.56, 169.41 ppm; anal. C 44.96, H 3.75, N 4.08% calcd for C_12_H_12_ClNO_3_S_2_, C 45.35, H 3.81, N 4.41%.

*N-(4-Chloro-2-carbamoylmethylthio-5-methylphenylsuflonyl)acetamide* (**20**). Starting from **5** (0.358 g) and acetic acid (2.5 mL) the title compound **20** was obtained (0.101 g, 60%) after 4.5 h at reflux, followed by additional cooling in an ice-water bath for 3 h: mp 220‒225 °C; IR (KBr) 3466, 3355 (NH), 2860, 2701 (CH_3_, CH_2_), 1693 (C=O), 1476, 1457 (N–H), 1160, 1119 (SO_2_) cm^−1^; ^1^H-NMR (500 MHz, DMSO-*d*_6_) δ 1.93 (s, 3H, CH_3_CO), 2.34 (s, 3H, CH_3_), 3.81 (s, 2H, SCH_2_), 7.27 (s, 1H, CONH_2_), 7.60 (s, 1H, CONH_2_), 7.62 (s, 1H, H-3), 7.89 (s, 1H, H-6), 12.30 (s, 1H, NH); ^13^C-NMR (125 MHz, DMSO-*d*_6_) δ 19.63, 23.83, 36.95, 128.79, 133.27, 134.16, 135.21, 136.77, 139.63, 169.42, 169.68 ppm; anal. C 38.84, H 3.78, N 7.95% calcd for C_11_H_13_ClN_2_O_4_S_2_, C 39.23, H 3.89, N 8.32%.

*N-(2-Benzylthio-4-chloro-5-methylphenylsulfonyl)acetamide* (**21**). Starting from **6** (0.400 g) and acetic acid (2.5 mL) the title compound **21** was obtained (0.200 g, 54%) after 2 h at reflux, followed by additional cooling in an ice-box for 12 h: mp 155‒158 °C; IR (KBr) 3116 (N–H), 2921 (CH_3_, CH_2_), 1686 (C=O), 1454 (N–H), 1356, 1165 (SO_2_) cm^−1^; ^1^H-NMR (200 MHz, DMSO-*d*_6_) δ 1.92 (s, 3H, CH_3_CO), 2.35 (s, 3H, CH_3_), 4.45 (s, 2H, SCH_2_), 7.26‒7.48 (m, 5H, arom.), 7.67 (s, 1H, H-3), 7.90 (s, 1H, H-6), 12.36 (s, 1H, NH); ^13^C-NMR (125 MHz, DMSO-*d*_6_) δ 19.64, 23.76, 36.77, 128.14, 128.73, 129.18, 129.81, 133.08, 134.16, 135.13, 136.62, 139.59, 169.26 ppm; anal. C 51.89, H 4.35, N 3.77% calcd for C_16_H_16_ClNO_3_S_2_, C 51.95, H 4.36, N 3.79%.

*N-(4-Chloro-5-methyl-2-(3-trifluoromethylbenzylthio)phenylsulfonyl)acetamide* (**22**). Starting from **7** (0.459 g) and acetic acid (2.5 mL) the title compound **22** was obtained (0.367 g, 84%) after 2 h at reflux, followed by additional cooling an ice-box for 12 h: mp 126‒130 °C; IR (KBr) 3221 (N–H), 3065 (C–H arom.), 2986, 2855 (CH_3,_ CH_2_), 1721 (C=O), 1448 (N–H), 1330, 1164 ( SO_2_) cm^−1^; ^1^H-NMR (200 MHz, DMSO-*d*_6_) δ 1.92 (s, 3H, CH_3_CO), 2.35 (s, 3H, CH_3_), 4.58 (s, 2H, SCH_2_),7.56‒7.82 (m, 5H, H-3, arom.), 7.91 (s, 1H, H-6), 12.37 (s, 1H, NH) ppm; ^13^C-NMR (50 MHz, DMSO-*d*_6_) δ 19.22, 23.24, 35.66, 124.43, 124.51, 126.06, 126.13, 128.76, 129.10, 129.73, 129.87, 133.12, 133.36, 133.80, 135.11, 135.32, 138.11, 139.21, 168.82 ppm; anal. C 46.49, H 3.42, N 3.13% calcd for C_17_H_15_ClF_3_NO_3_S_2_, C 46.63, H 3.45, N 3.20%.

*N-(4-Chloro-5-methyl-2-(4-trifluoromethylbenzylthio)phenylsulfonyl)acetamide* (**23**). Starting from **8** (0.459 g) and acetic acid (2.5 mL), the title compound **23** was obtained (0.219 g, 50%) after 1.5 h at reflux, followed by additional cooling in an ice-box for 12 h: mp 180‒181 °C; IR (KBr) 3443 (N–H), 3062 (C–H arom.), 2878, 2710 (CH_3_, CH_2_), 1687 (C=O), 1482 (N–H), 1321, 1160 (SO_2_) cm^−1^; ^1^H-NMR (200 MHz, DMSO-*d*_6_) δ 1.91 (s, 3H, CH_3_CO), 2.36 (s, 3H, CH_3_), 4.56 (s, 2H, SCH_2_), 7.63‒7.75 (m, 5H, H-3, arom.), 7.91 (s, 1H, H-6), 12.34 (s, 1H, NH) ppm; anal. C 46.66, H 3.45, N 3.21% calcd for C_17_H_15_ClF_3_NO_3_S_2_, C 46.63, H 3.45, N 3.20%.

*N-[4-Chloro-2-(4-chlorobenzylthio)-5-methylphenylsulfonyl]acetamide* (**24**). Starting from **9** (0.338 g) and acetic acid (2.5 mL), the title compound **24** was obtained (0.219 g, 50%) after 2 h at reflux, followed by additional cooling in an ice-box for 12 h: mp 182‒184 °C; IR (KBr) 3445 (N–H), 3052 (C–H arom.), 2869, 2711 (CH_3_, CH_2_), 1683 (C=O), 1490 (N–H), 1350, 1162 (SO_2_) cm^−1^; ^1^H-NMR (200 MHz, DMSO-*d*_6_) δ 1.92 (s, 3H, CH_3_CO), 2.35 (s, 3H, CH_3_), 4.46 (s, 2H, CH_2_S), 7.42 (d, 2H, arom.), 7.47 (d, 2H, arom.), 7.68 (s, 1H, H-3), 7.81 (s, 1H, H-6), 12.35 (s, 1H, NH); ^13^C-NMR (125 MHz, DMSO-*d*_6_) δ 19.65, 23.75, 35.97, 128.95, 129.16, 131.57, 132.74, 133.34, 134.18, 135.32, 135.93, 136.12, 139.62, 169.28 ppm; anal. C 47.26, H 3.69, N 3.33% calcd for C_16_H_15_ClNO_3_S_2_, C 47.53, H 3.74, N 3.46%.

*N-[4-Chloro-2-(4-methoxybenzylthio)-5-methylphenylsulfonyl]acetamide* (**25**). Starting from **10** (0.421 g) and acetic acid (2.5 mL) the crude product was obtained after refluxing for 1 h, then evaporating the mixture under reduced pressure. Crystallization from ethanol gave the title compound **25** (0.204 g, 51%): mp 82‒84 °C; IR (KBr) 3427 (N–H), 2924, 2853 (CH_3_, CH_2_), 1705 (C=O), 1450 (N–H), 1303, 1032 (SO_2_) cm^−1^; ^1^H-NMR (200 MHz, DMSO-*d*_6_) δ 1.79 (s, 3H, CH_3_CO), 2.32 (s, 3H, CH_3_), 3.74 (s, 3H, CH_3_O), 4.25 (s, 2H, CH_2_S), 6.90 (d, 2H, arom.), 7.35 (d, 2H, arom.), 7.45 (s, 1H, H-3), 7.81 (s, 1H, H-6), 12.20 (br s, 1H, NH) ppm; anal. C 51.20, H 4.57, N 3.58% calcd for C_17_H_18_ClNO_4_S_2_, C 51.06, H 4.54, N 3.50%.

*N-[4-Chloro-2-(4-pyridinylmethylthio)-5-methylphenylsulfonyl]acetamide* (**26**). Starting from **11** (0.392 g) and acetic acid (2.5 mL) the crude product was obtained after 1.5 h at reflux, then evaporating the mixture under reduced pressure. Crystallization from ethanol/chloroform (*v*/*v* = 2:1) gave the title compound **26** (0.167 g, 45%): mp 179‒183 °C; IR (KBr) 3413 (N–H), 2923, 2852 (CH_3_, CH_2_), 1711 (C=O), 1492 (N–H), 1325, 1153 (SO_2_) cm^−1^; ^1^H-NMR (200 MHz, DMSO-*d*_6_) δ 1.92 (s, 3H, CH_3_CO), 2.34 (s, 3H, CH_3_), 4.49 (s, 2H, CH_2_S), 7.44 (d, 2H, pyridine), 7.67 (s, 1H, H-3), 7.90 (s, 1H, H-6), 8.52 (d, 2H, pyridine), 12.40 (s, 1H, NH) ppm; anal. C 48.39, H 4.00, N 7.45% calcd for C_15_H_15_ClN_2_O_3_S_2_, C 48.58, H 4.08, N 7.55%.

*N-[4-Chloro-5-methyl-2-(naphth-1-ylmethyl)phenylsulfonyl]acetamide* (**27**). Starting from **12** (0.442 g) and acetic acid (2.5 mL) a white solid was obtained after refluxing for 1.5 h, followed by additional cooling in an ice-box for 12 h. The product was crystallized from acetonitrile to afford the title compound **27** (0.227 g, 54%): mp 176‒178 °C; IR (KBr) 3438 (N–H), 3061 (C–H arom), 2921, 2852 (CH_3_, CH_2_), 1727 (C=O), 1443 (N–H), 1352, 1155 (SO_2_) cm^−1^; ^1^H-NMR (200 MHz, DMSO-*d*_6_) δ 1.92 (s, 3H, CH_3_CO), 2.39 (s, 3H, CH_3_), 4.91 (s, 2H, CH_2_S), 7.45‒7.66 (m, 4H, arom.), 7.77 (s, 1H, H-3) 7.89‒8.00 (m, 3H, H-6 and arom.), 8.25 (d, 1H, arom.), 12.20 (s, 1H, NH) ppm; anal. C 57.23, H 4.32, N 3.33% calcd for C_20_H_18_ClNO_3_S_2_, C 57.20, H 4.32, N 3.34%.

*N-[4-Chloro-5-methyl-2-(naphth-2-ylmethyl)phenylsulfonyl]acetamide* (**28**). Starting from **13** (0.442 g) and acetic acid (2.5 mL) the title compound **28** was obtained (0.210 g, 50%) after 2 h at reflux, followed by additional cooling in an ice-box for 12 h: mp 138‒141 °C; IR (KBr) 3423 (N–H); 3066 (C–H arom.); 2919, 2870 (CH_3_, CH_2_), 1683 (C=O), 1353, 1161 (SO_2_) cm^−1^; ^1^H-NMR (200 MHz, DMSO-*d*_6_) δ 1.93 (s, 3H, CH_3_CO), 2.33 (s, 3H, CH_3_), 4.63 (s, 2H, CH_2_S), 7.40‒7.60 (m, 3H, arom.), 7.75 (s, 1H, H-3), 7.80‒7.96 (m, 5H, H-6 and arom.), 12.36 (s, 1H, NH) ppm; anal. C 57.30, H 4.30, N 3.39% calcd for C_20_H_18_ClNO_3_S_2_, C 57.20, H 4.32, N 3.34%.

*N-[4-Chloro-5-methyl-2-(quinolin-2-yl)phenylsulfonyl]acetamide* (**29**). Starting from **14** (0.421 g) and acetic acid (2.5 mL), the title compound **29** was obtained (0.219 g, 52%) after refluxing with stirring for 2 h, followed by additional cooling in an ice-box for 12 h: mp 153.8‒157 °C; IR (KBr) 3421 (N–H), 3069 (C–H arom.), 2922, 2851 (CH_3_, CH_2_), 1719 (C=O), 1457 (N–H), 1351, 1157 (SO_2_) cm^−1^; ^1^H-NMR (200 MHz, DMSO-*d*_6_) δ 1.91 (s, 3H, CH_3_CO), 2.32 (s, 3H, CH_3_), 3.36 (s, 2H, CH_2_S), 7.60 (t, 1H, quinol.), 7.68 (s, 1H, H-3), 7.79 (t, 1H, quinol.), 7.88 (s, 1H, H-6), 7.90‒8.00 (m, 3H, quinol.), 8.37 (d, 1H, quinol.), 12.40 (s, 1H, NH) ppm; anal. C 54.60, H 3.92, N 6.37% calcd for C_19_H_17_ClN_2_O_3_S_2_, C 54.21, H 4.07, N 6.66%.

*N-[4-Chloro-2-(2,3-dihydrobenzo[b][1,4]dioxin-2-ylmethylthio)-5-methylphenylsulfonyl]acetamide* (**30**). Starting from **15** (0.449 g) and acetic acid (2.5 mL), the title compound **30** was obtained (0.235 g, 55%) after refluxing for 1 h 15 min, followed by additional cooling in an ice-box for 12 h: mp 188‒191.5 °C; IR (KBr) 3268 (N–H), 3067 (C–H arom.), 2985, 2883 (CH_3_, CH_2_), 1722 (C=O), 1495 (N–H), 1329, 1159 (SO_2_) cm^−1^; ^1^H-NMR (200 MHz, DMSO-*d*_6_) δ 1.92 (s, 3H, CH_3_CO), 2.38 (s, 3H, CH_3_), 3.50 (d, 2H, CH_2_O), 4.14 (dd, 1H, CHO), 4.38 (d, 2H, CH_2_S), 6.66–6.89 (m, 4H, arom.), 7.74 (s, 1H, H-3), 7.94 (s, 1H, H-6), 12.41 (s, 1H, NH) ppm; anal. C 50.50, H 4.24, N 3.28% calcd for C_18_H_18_ClNO_5_S_2_, C 50.52, H 4.24, N 3.27%.

*N-[4-Chloro-2-(6-chlorobenzo[d][1,3]dioxol-5-ylmethylthio)-5-methylphenylsulfonyl]acetamide* (**31**). Starting from **16** (0.469 g) and acetic acid (2.5 mL) the crude product was obtained after 1.5 h at reflux, followed by additional cooling in an ice-box for 12 h,. Crystallization from ethanol/chloroform (*v*/*v* = 2.7:1) gave the title compound **31** (0.264 g, 59%): mp 122‒124 °C; IR (KBr) 3424 (N–H), 2922, 2852 (CH_3_, CH_2_), 1719 (C=O), 1478 (N–H), 1251, 1159 (SO_2_) cm^−1^; ^1^H NMR (200 MHz, DMSO-*d*_6_) δ 1.81 (s, 3H, CH_3_CO), 2.34 (s, 3H, CH_3_), 4.30 (s, 2H, CH_2_S), 6.08 (s, 2H, CH_2_O), 7,12 (s, 1H, arom.), 7.13 (s, 1H, arom.), 7.44 (s, 1H, H-3), 7.85 (s, 1H, H-6), 12.40 (s, 1H, NH) ppm; anal. C 45.71, H 3.32, N 3.03% calcd for C_17_H_15_C_l2_NO_5_S_2_, C 45.54, H 3.37, N 3.12%.

*N-[4-Chloro-2-(3,5-dimethylisoxazol-4-ylmethylthio)-5-methylphenylsulfonyl]acetamide* (**32**). Starting from **17** (0.338 g) and acetic acid (2.5 mL), a product was obtained after 1.5 h at reflux, followed by additional cooling in an ice-box for 12 h. Crystallization from 80% ethanol gave the title compound **32** (0.241 g, 62%): mp 154‒160 °C; IR (KBr) 3421 (N–H), 3086 (C–H arom.), 2922, 2853 (CH_3_, CH_2_), 1711 (C=O), 1455 (N–H), 1351, 1161 (SO_2_) cm^−1^; ^1^H-NMR (200 MHz, DMSO-*d*_6_) δ 1.92 (s, 3H, CH_3_CO), 2.26 (s, 3H, CH_3_), 2.37 (s, 3H, CH_3_), 2.39 (s, 3H, CH_3_), 4.28 (s, 2H, CH_2_S), 7.73 (s, 1H, H-3), 7.93 (s, 1H, H-6), 12.32 (s, 1H, NH) ppm; anal. C 45.95, H 4.25, N 6.88% calcd for C_15_H_17_ClN_2_O_4_S_2_, C 46.33, H 4.41, N 7.20%.

*N-(2-Benzylthio-4-chloro-5-methylphenylsulfonyl)propionamide* (**33**). Starting from **6** (0.385 g) and propionic acid (3.0 mL) the product was obtained after 1 h at reflux, then evaporating the mixture under reduced pressure and treating the residue with water (10 mL). Crystallization of the crude product from ethyl acetate/petroleum ether gave the title compound **33** (0.330 g, 86%): mp 137‒139 °C; IR (KBr) 3440 (N–H), 3033 (C–H arom.), 2922, 2874 (CH_3_, CH_2_), 1673 (C=O), 1469 (N–H), 1355, 1162 (SO_2_) cm^−1^; ^1^H-NMR (200 MHz, DMSO-*d*_6_) δ 0.89 (t, 3H, CH_3_CH_2_CO), 2.23 (q, 2H, CH_2_CO), 2,36 (s, 3H, CH_3_), 4.44 (s, 2H, CH_2_S), 7.24‒7.45 (m, 5H, arom.), 7.67 (s, 1H, H-3), 7.92 (s, 1H, H-6), 12.31 (s, 1H, NH); ^13^C-NMR (125 MHz, DMSO-*d*_6_) δ 8.94, 19.65, 29.23, 36.73, 128.13, 128.65, 129.17, 129.78, 133.08, 134.19, 135.17, 136.57, 136.62, 139.57, 172.77 ppm; anal. C 53.39, H 4.83, N 3.90% calcd for C_17_H_18_ClNO_3_S_2_, C 53.18, H 4.73, N 3.65%.

*N-[4-Chloro-5-methyl-2-(3-(trifluoromethylbenzylthio)phenylsulfonyl]propionamide* (**34**). Starting from **7** (0.459 g) and propionic acid (3.0 mL) the product was obtained after refluxing for 2 h, then evaporating the mixture under reduced pressure and treating of residue with water (10 mL). Crystallization from 80% ethanol gave the title compound **34** (0.307 g, 68%): mp 123‒127 °C; IR (KBr) 3242 (N–H), 2926, 2853 (CH_3_, CH_2_), 1726 (C=O), 1431 (N–H), 1326, 1128 (SO_2_) cm^−1^; ^1^H-NMR (200 MHz, DMSO-*d*_6_) δ 0.87 (t, 3H, CH_3_CH_2_CO), 2.27 (q, 2H, CH_2_CO), 2.35 (s, 3H, CH_3_), 4.56 (s, 2H, CH_2_S), 7.60‒7.75 (m, 4H, H-3, arom.), 7.80 (m, 1H, arom.), 7.91 (s, 1H, H-6), 12.32 (s, 1H, NH) ppm; anal. C 47.94, H 3.82, N 3.15% calcd for C_18_H_17_ClF_3_NO_3_S_2_, C 47.84, H 3.79, N 3.10%.

*N-[4-Chloro-5-methyl-2-(4-trifluoromethylbenzylthio)phenylsulfonyl]propionamide* (**35**). Starting from **8** (0.451 g) and propionic acid (3.0 mL) the product was obtained after refluxing for 1 h, then evaporating the mixture under reduced pressure and treating of residue with water (10 mL). Crystallization from ethyl acetate/petroleum ether gave the title compound **35** (0.420 g, 93%): mp 107‒108 °C; IR (KBr) 3218 (N–H), 2988, 2952 (CH_3_, CH_2_), 1734 (C=O), 1325, 1122 (SO_2_) cm^−1^; ^1^H-NMR (200 MHz, DMSO-*d*_6_) δ 0.86 (t, 3H, CH_3_CH_2_CO), 2.19 (q, 2H, CH_2_CO), 2.36 (s, 3H, CH_3_), 4.56 (s, 2H, CH_2_S), 7.66‒7.72 (m, 5H, H-3, arom.), 7.92 (s, 1H, H-6), 12.31 (s, 1H, NH) ppm; anal. C 47.96, H 3.81, N 3.16% calcd for C_18_H_17_ClF_3_NO_3_S_2_, C47.84, H 3.79, N 3.10%.

*N-[4-Chloro-2-(4-chlorobenzylthio)-5-methylphenylsulfonyl]propionamide* (**36**). Starting from **9** (0.338 g) and propionic acid (3.0 mL) the product was obtained after refluxing for 2 h, then evaporating the mixture under reduced pressure and treating of residue with water (10 mL). Crystallization from 96% ethanol gave the title compound **36** (0.284 g, 68%): mp 87‒91 °C; IR (KBr) 3116 (N–H), 2927, 2865 (CH_3_, CH_2_), 1719 (C=O), 1456 (N–H), 1319, 1132 (SO_2_) cm^−1^; ^1^H-NMR (200 MHz, DMSO-*d*_6_) δ 0.88 (t, 3H, CH_3_CH_2_CO), 2,26 (q, 2H, CH_2_CO), 2,36 (s, 3H, CH_3_), 4,44 (s, 2H, CH_2_S), 7,36 (d, 2H, arom.), 7.44 (d, 2H, arom.), 7.67 (s, 1H, H-3), 7.91 (s, 1H, H-6), 12.27 (s, 1H, NH) ppm; anal. C 48.98, H 4.15, N 3.46% calcd for C_17_H_17_Cl_2_NO_3_S_2_, C 48.81, H 4.10, N 3.35%.

*N-[4-Chloro-2-(4-methoxybenzylthio)-5-methylphenylsulfonyl]propionamide* (**37**). Starting from **10** (0.421 g) and propionic acid (3.0 mL) the product was obtained after refluxing for 1 h, then evaporating the mixture under reduced pressure and treating of residue with water (10 mL). Crystallization from benzene gave the title compound **37** (0.352 g, 85%): mp 130‒132 °C; IR (KBr) 3268 (N–H), 3032 (C–H arom.), 2935, 2853 (CH_3_, CH_2_), 1732 (C=O), 1441 (N–H), 1349, 1175 (SO_2_) cm^−1^; ^1^H-NMR (200 MHz, DMSO-*d*_6_) δ 0.89 (t, 3H, CH_3_CH_2_CO), 2.22 (q, 2H, CH_2_CO), 2.36 (s, 3H, CH_3_), 3.73 (s, 3H, OCH_3_), 4.37 (s, 2H, CH_2_S), 6.89 (d, 2H, arom.), 7.34 (d, 2H, arom.), 7.66 (s, 1H, H-3), 7.91 (s, 1H, H-6), 12.27 (s, 1H, NH); ^13^C-NMR (125 MHz, DMSO-*d*_6_) δ 8.92, 19.65, 29.22, 36.29, 55.75, 114.56, 128.20, 128.65, 129.00, 130.99, 132.97, 134.15, 135.13, 136.79, 139.55, 159.27, 172.75 ppm; anal. C 52.11, H 4.85, N 3.32% calcd for C_18_H_20_ClNO_4_S_2_, C 52.23, H 4.87, N 3.38%.

*N-[4-Chloro-5-methyl-2-(pyridin-4-ylmethylthio)phenylsulfonyl]propionamide* (**38**). Starting from **11** (0.392 g) and propionic acid (3.0 mL) the product was obtained after refluxing for 2 h 15 min, then evaporating the mixture under reduced pressure and treating of residue with water (10 mL). Crystallization from ethanol gave the title compound **38** (0.250 g, 65%): mp 151‒154 °C; IR (KBr) 3422 (N–H), 2925, 2852 (CH_3_, CH_2_), 1715 (C=O), 1349, 1150 (SO_2_) cm^−1^; ^1^H-NMR (200 MHz, DMSO-*d*_6_) δ 0.88 (t, 3H, CH_3_CH_2_CO), 2.24 (q, 2H, CH_2_CO), 2.35 (s, 3H, CH_3_), 4.50 (s, 2H, CH_2_S), 7.44 (d, 2H, pyridine), 7.68 (s, 1H, H-3), 7.92 (s, 1H, H-6), 8.51 (d, 2H, pyridine), 12.38 (s, 1H, NH); ^13^C-NMR (125 MHz, DMSO-*d*_6_) δ 8.91, 19.66, 29.25, 35.25, 124.69, 128.87, 133.57, 134.29, 135.46, 135.59, 139.63, 146.34, 150.35, 172.80 ppm; anal. C 50.02, H 4.47, N 7.32% calcd for C_16_H_17_ClN_2_O_3_S_2_, C 49.93, H 4.45, N 7.28%.

*N-[4-Chloro-5-methyl-2-(naphthalen-2-ylmethylthio)phenylsulfonyl]propionamide* (**39**). Starting from **13** (0.441 g) and propionic acid (3.0 mL) the product was obtained after refluxing for 1.5 h, then evaporating the mixture under reduced pressure and treating of residue with water (10 mL). Crystallization from 80% ethanol gave the title compound **39** (0.373 g, 86%): mp 85‒88 °C; IR (KBr) 3207 (N–H), 3056 (C–H arom.), 2920, 2855 (CH_3_, CH_2_), 1731 (C=O), 1446 (N–H), 1321, 1176 (SO_2_) cm^−1^; ^1^H-NMR (200 MHz, DMSO-*d*_6_) δ 0.84 (t, 3H, CH_3_CH_2_CO), 2.24 (q, 2H, CH_2_CO), 2.33 (s, 3H, CH_3_), 4.62 (s, 2H, CH_2_S), 7.49‒7.60 (m, 4H, arom.), 7.75 (s, 1H, H-3), 7.83‒7.94 (m, 4H, H-6, arom.), 12.30 (s, 1H, NH) ppm; anal. C 58.48, H 4.80, N 3.55% calcd for C_21_H_20_ClNO_3_S_2_, C 58.12, H 4.65, N 3.23%.

*N-[4-Chloro-2-(2,3-dihydrobenzo[b][1,4]dioxin-2-ylmethylthio)-5-mehylphenylsulfonyl]propionamide* (**40**). Starting from **15** (0.449 g) and propionic acid (3.0 mL) the product was obtained after refluxing with stirring for 1 h 45 min, then evaporating the mixture under reduced pressure and treating of residue with water (10 mL). Crystallization from ethanol gave the title compound **40** (0.384 g, 87%): mp 114‒116.2 °C; IR (KBr) 3224 (N–H), 2920, 2852 (CH_3_, CH_2_), 1730 (C=O), 1495 (N–H), 1270, 1136 (SO_2_) cm^−1^; ^1^H-NMR (500 MHz, DMSO-*d*_6_) δ 0.83 (t, 3H, CH_3_CH_2_CO), 2.14 (q, 2H, CH_2_CO), 2.34 (s, 3H, CH_3_), 3.36‒3.47 (m, 2H, CH_2_O), 4.06 (dd, 1H, CHO), 4.32 (d, 2H, CH_2_S), 6.70‒6.85 (d, 4H, arom.), 7.63 (s, 1H, H-3), 7.88 (s, 1H, H-6), 12.26 (s, 1H, NH) ppm; ^13^C-NMR (50 MHz, DMSO-*d*_6_) δ 9.11, 19.31, 29.95, 33.28, 66.23, 72.08, 117.17, 117.29, 121.66, 121.79, 128.82, 132.73, 133.23, 135.02, 137.93, 142.57, 142.98, 174.53 ppm; anal. C 51.73, H 4.58, N 3.22% calcd for C_19_H_20_ClNO_5_S_2_, C 51.64, H 4.56, N 3.17%.

*N-[4-Chloro-2-(6-chlorobenzo[d][1,3]dioxol-5-ylmethylthio)-5-methylphenylsulfonyl]propionamide* (**41**). Starting from **16** (0.469 g) and propionic acid (3.0 mL) the product was obtained after refluxing for 1 h, then evaporating the mixture under reduced pressure and treating of residue with water (10 mL). Crystallization from ethyl acetate/petroleum ether gave the title compound **41** (0.376 g, 81%): mp 142‒144 °C; IR (KBr) 3205 (N–H), 2922, 2898 (CH_3_, CH_2_), 1686 (C=O), 1474 (N–H), 1250, 1116 (SO_2_) cm^−1^; ^1^H-NMR (200 MHz, DMSO-*d*_6_) δ 0.88 (t, 3H, CH_3_CH_2_CO), 2.23 (q, 2H, CH_2_CO), 2.38 (s, 3H, CH_3_), 4.39 (s, 2H, CH_2_S), 6.08 (s, 2H, CH_2_), 7.11 (s, 2H, arom.), 7.62 (s, 1H, H-3), 7.93 (s, 1H, H-6), 12.26 (s, 1H, NH); ^13^C-NMR (125 MHz, DMSO-*d*_6_) δ 8.87, 19.68, 29.20, 35.34, 102.90, 110.33, 111.13, 126.15, 126.89, 128.80, 133.38, 134.23, 135.33, 136.46, 139.66, 147.32, 148.48, 172.80 ppm; anal. C 46.70, H 3.70, N 3.01% calcd for C_18_H_17_ClNO_5_S_2_, C 46.76, H 3.71, N 3.03%.

*N-[4-Chloro-2-(3,5-dimethylisoxazol-4-ylmethylthio)-5-methylphenylsulfonyl]propionamide* (**42**). Starting from **17** (0.339 g) and propionic acid (3.0 mL) the product was obtained after refluxing for 1.5 h, then evaporating the mixture under reduced pressure and treating of residue with water (10 mL). Crystallization from ethanol/chloroform (*v*/*v* = 2:1) gave the title compound **42** (0.374 g, 68%): mp 160‒163 °C; IR (KBr) 3433 (N–H), 3064 (C–H arom.), 2935, 2859 (CH_2_, CH_2_), 1726 (C=O), 1455 (N–H), 1353, 1132 (SO_2_) cm^−1^; ^1^H-NMR (200 MHz, DMSO-*d*_6_) δ 0.89 (t, 3H, CH_3_CH_2_CO), 2.17‒2.25 (m, 5H, CH_2_CO and CH_3_), 2.37 (s, 3H, CH_3_), 2.39 (s, 3H, CH_3_), 4.28 (s, 2H, CH_2_S), 7.71 (s, 1H, H-3), 7.94 (s, 1H, H-6), 12.28 (s, 1H, NH); ^13^C-NMR (125 MHz, DMSO-*d*_6_) δ 8.91, 10.31, 11.29, 19.69, 25.35, 29.15, 109.37, 129.65, 133.61, 134.18, 135.78, 136.14, 139.57, 160.22, 167.67, 172.78 ppm; anal. C 47.75, H 4.75, N 6.97% calcd for C_16_H_19_ClN_2_O_4_S_2_ C 47.70, H 4.75, N 6.95%.

*N-(2-Benzylthio-4-chloro-5-methylphenylsulfonyl)isobutyramide* (**43**). Starting from **6** (0.391 g) and isobutyric acid (3.7 mL) the product was obtained after refluxing for 2 h, then dropping water (10 mL) into the cooled reaction mixture. Crystallization from ethanol gave the title compound **43** (0.378 g, 95%): mp 122‒125 °C; IR (KBr) 3423 (N–H), 2926, 2868 (CH_3_, CH_2_), 1694 (C=O), 1433 (N–H), 1351, 1172 (SO_2_) cm^−1^; ^1^H-NMR (200 MHz, DMSO-*d*_6_) δ 0.96 (d, 6H, CH_3_), 2.36 (s, 3H, CH_3_), 2.40‒2.60 (m, 1H, CH); 4.43 (s, 2H, CH_2_S), 7.20‒7.45 (m, 5H, arom.), 7.64 (s, 1H, H-3), 7.92 (s, 1H, H-6), 12.28 (s, 1H, NH) ppm; ^13^C-NMR (50 MHz, DMSO-*d*_6_) δ 24.14, 24.66, 39.81, 41.90, 133.18, 133.45, 134.23, 134.84, 138.00, 139.31, 140.03, 141.41, 141.81, 144.59, 180.90 ppm; anal. C 54.39, H 5.07, N 3.55% calcd for C_18_H_20_ClNO_3_S_2_, C 54.33, H 5.07, N 3.52%.

*N-(2-Benzylthio-4-chloro-5-methylphenylsulfonyl)-3-cyclohexylpropionamide* (**44**). Starting from **6** (0.338 g) and 3-cyclohexenepropionic acid (6.3 mL) the crude product was obtained after refluxing for 1.5 h at 115 °C. Crystallization from acetonitrile gave the title compound **44** (0.378 g, 81%): mp 118‒120 °C; IR (KBr) 3427 (N–H), 3060 (C–H arom.), 2923, 2840 (CH_3_, CH_2_), 1678 (C=O), 1448 (N–H), 1359, 1171 (SO_2_) cm^−1^; ^1^H-NMR (200 MHz, DMSO-*d*_6_) δ 0.8‒0.98 (m, 2H, cHex.), 1.10‒1.20 (m, 4H, cHex.), 1.40 (q, 2H, CH_2_), 1.50‒1,80 (m, 5H, cHex.), 2.22 (t, 2H, CH_2_CO), 2.36 (s, 3H, CH_3_), 4.43 (s, 2H, CH_2_S), 7.20‒7.45 (m, 5H, arom.), 7.64 (s, 1H, H-3), 7.92 (s, 1H, H-6), 12.29 (s, 1H, NH) ppm; anal. C 59.19, H 6.04, N 2.97% calcd for C_23_H_28_ClNO_3_S_2_, C 59.27, H 6.06, N 3.01%.

*N-(2-Benzylthio-4-chloro-5-methylphenylsulfonyl)benzamide* (**45**). A mixture of **6** (0.391 g, 1 mmol) and benzoic acid (0.244 g, 2 mmol) in water (4 mL) was stirred in a pressure tube at 100 °C for 144 h. The product was isolated from the hot reaction mixture by filtration. After crystallization from 90% ethanol the title compound **45** was obtained (0.311 g, 72%): mp 168‒170 °C; IR (KBr): 3274 (N–H), 3064 (C–H arom.), 2918 (CH_3_, CH_2_), 1693 (C=O), 1453 (N–H), 1332, 1163 (SO_2_) cm^−1^; ^1^H-NMR (200 MHz, DMSO-*d*_6_) δ 2.34 (s, 3H, CH_3_), 4.40 (s, 2H, CH_2_S), 7.10‒7.20 (m, 3H, arom.), 7.24‒7.36 (m, 2H, arom.), 7.45‒7.58 (m, 2H, arom.), 7.60‒7.72 (m, 2H, H-3 and arom.), 7.88‒7.98 (d, 2H, arom.), 8.02 (s, 1H, H-6), 12.82 (s, 1H, NH); ^13^C-NMR (125 MHz, DMSO-*d*_6_) δ 19.69, 36.65, 128.02, 128.66, 129.03, 129.23, 129.65, 131.98, 133.12, 133.98, 134.44, 135.16, 136.40, 136.66, 139.68, 165.86 ppm; anal. C 58.19, H 4.15, N 3.15% calcd for C_21_H_18_ClNO_3_S_2_, C 58.39, H 4.20, N 3.24%.

*N-(2-Benzylthio-4-chloro-5-methylphenylsulfonyl)-3-phenylacrylamide* (**46**). A mixture of **6** (0.391 g, 1 mmol) and cinnamic acid (0.296 g, 2 mmol) in water (6 mL) was stirred in a pressure tube at 100 °C for 96 h. The product was isolated from the hot reaction mixture by filtration. After crystallization from 80% ethanol the title compound **46** was obtained (0.348 g, 76%): mp 173‒174 °C; IR (KBr) 3199 (N–H), 2919, 2854 (CH_3_, CH_2_), 1675 (C=O), 1439 (N–H), 1353, 1131 (SO_2_) cm^−1^; ^1^H-NMR (200 MHz, DMSO-*d*_6_) δ 2.37 (s, 3H, CH_3_), 4.43 (s, 2H, CH_2_S), 6.74 (d, 1H, CH=), 7.16‒7.24 (m, 3H, arom.), 7.34‒7.68 (m, 9H, arom.), 7.99 (s, 1H, H-6), 12.54 (s, 1H, NH) ppm; ^13^C-NMR (50 MHz, DMSO-*d*_6_) δ 19.25, 36.37, 118.99, 127.59, 128.42, 128.49, 128.62, 129.31, 129.43, 131.04, 132.77, 133.97, 134.13, 134.79, 136.08, 139.26, 144.23, 163.43 ppm; anal. C 60.30, H 4.40, N 3.05% calcd for C_23_H_20_ClNO_3_S_2_, C 60.32, H 4.40, N 3.06%.

*N-[4-Chloro-5-methyl-2-(naphthalen-1-ylmethylthio)phenylsulfonyl]-3-phenylacrylamide* (**47**). A mixture of **12** (0.450 g, 1 mmol) and cinnamic acid (0.148 g, 1.1 mmol) in toluene (5 mL) was stirred in a pressure tube at 110‒120 °C for 120 h. A solvent was evaporated under reduced pressure to dryness and residue was treated with water (150 mL) and stirred using an ultrasonic bath for 15 min. Product was isolated by extraction with chloroform (3 × 50 mL), then the combined organic layers were washed with 1% NaHCO_3_ (2 × 50 mL), 1 M HCl (50 mL), water (2 × 50 mL) and dried with MgSO_4_. The solvent was evaporated under reduced pressure and residue was purified by crystallization from ethanol giving the titled compound **47** (0.254 g, 50%): mp 105‒106 °C; IR (KBr) 3192 (N–H), 2923, 2853 (CH_3_, CH_2_), 1683 (C=O), 1448 (N–H), 1351, 1178 (SO_2_) cm^−1^; ^1^H-NMR (200 MHz, DMSO*-d*_6_) δ 2.42 (s, 3H, CH_3_), 4.90 (s, 2H, CH_2_S), 6.58 (d, 1H, CH=), 7.35‒7.64 (m, 10H, CH= and arom.), 7.77 (s, 1H, H-3), 7.82‒7.94 (m, 2H, arom.), 8.03 (s, 1H, H-6), 8.18 (d, 1H, arom.), 12.40 (s, 1H, NH); ^13^C-NMR (125 MHz, DMSO-*d*_6_) δ 19.70, 35.22, 119.30, 124.81, 126.09, 126.57, 127.00, 128.79, 128.90, 129.09, 129.15, 129.79, 131.47, 131.72, 132.01, 133.13, 134.08, 134.32, 134.48, 134.83, 137.35, 139.81, 144.59, 163.87 ppm; anal. C 63.70, H 4.33, N 2.69% calcd for C_27_H_22_ClNO_3_S_2_, C 63.83, H 4.36, N 2.76%.

### 3.3. X-ray Structure Determination

Experimental diffraction data were collected on a KM4 CCD kappa-geometry diffractometer (Oxford Diffraction, Yarnton, Oxfordshire, UK), equipped with a Sapphire2 CCD detector. An enhanced X-ray Mo Ka radiation source with a graphite monochromator was used. Determination of the unit cell and diffraction data collection were carried out at room temperature (298 K). All calculations (data reduction, structure solution, and refinement) were carried out using CrysAlisPro package [40]. The structure was solved by direct methods, and all nonhydrogen atoms were refined with anisotropic thermal parameters by full-matrix least squares procedure based on *F*^2^. Final refinements were carried out using the SHELX-97 package [41], run under control of WinGX program [42]. Scattering power of all the crystals tested was low, so in spite of long frame exposure time (240 s), the ratio of observed to unique reflections is only 40%.

Crystallographic data for structure of **22** reported in this paper have been deposited with the Cambridge Crystallographic Data Center as supplementary publication No. CCDC 1400372. Copies of the data can be obtained free of charge on application to CCDC, 12 Union Road, Cambridge CB2 1EZ, UK (Fax: +44-1223-336-033; Email: deposit@ccdc.cam.ac.uk).

### 3.4. Cell Culture and Cell Viability Assay

All chemicals, if not stated otherwise, were obtained from Sigma-Aldrich (St. Louis, MO, USA). The MCF-7 cell line was purchased from Cell Lines Services (Eppelheim, Germany), the HeLa and HCT-116 cell lines were obtained from the Department of Microbiology, Tumor and Cell Biology, Karolinska Institute (Stockholm, Sweden). Cells were cultured in in Dulbecco’s modified Eagle’s medium (DMEM) supplemented with 10% fetal bovine serum, 2 mM glutamine, 100 units/mL penicillin, and 100 μg/mL streptomycin. Cultures were maintained in a humidified atmosphere with 5% CO_2_ at 37 °C in an incubator (HeraCell, Heraeus, Langenselbold, Germany).

Cell viability was determined using the MTT (3-(4,5-dimethylthiazol-2-yl)-2,5-diphenyl-tetrazolium bromide) assay. Cells were seeded in 96-well plates at a density of 5 ×10^3^ cells/well and treated for 72 h with the examined compounds in the concentration range 1–100 μM (1, 10, 25, 50 and 100 μM). Following treatment, MTT (0.5 mg/mL) was added to the medium and cells were further incubated for 2 h at 37 °C. Cells were lysed with DMSO and the absorbance of the formazan solution was measured at 550 nm with a plate reader (1420 multilabel counter, Victor, Jügesheim, Germany). The optical density of the formazan solution was measured at 550 nm with a plate reader (1420 multilabel counter, Victor, Germany). The experiment was performed in triplicate. Values are expressed as the mean ± SD of at least three independent experiments.

### 3.5. Molecular Modeling Methodology/Calculations

The two-dimensional structure of the 29 studied compounds were built using the graphical user interface of Gaussian software (Gaussian 03W, v 6.0, Gaussian Inc., Wallingford, CT, USA), before starting molecular modeling algorithms, in order to obtain the lowest energy geometry of a molecule. The resulting structures were optimized by means of a semi-empirical method (with the use of the AM1 Hamiltonian). The developed three-dimensional structures were submitted to descriptor calculations using Dragon software. Among over four thousand descriptors available, only selected blocks were calculated: Constitutional Descriptors, Ring Descriptors, Charge descriptors, Topological indices, Geometrical descriptors, Functional group counts, Atom centered fragments, Atom-type E-state indices, CASTS2D descriptors, 2D atom pairs, Molecular properties and subsequently used in the QSAR study.

### 3.6. QSAR Study

Multiple linear regression with stepwise algorithm was performed and evaluated in Statistica software (Statsoft, Tulsa, OK, USA). 473 descriptors were used as a candidate for independent variables and percent of growth (GP) was used as a dependent variable. Dataset was randomly divided into training (*n* = 22, 75% of compounds) and test set (*n* = 7, 25% of compounds).

### 3.7. Metabolic Stability

Stock solutions of studied compounds were prepared at concentration of 10 mM in DMSO. Working solutions were prepared by dilution of stock with reaction buffer or acetonitrile, final concentration of organic solvent did not exceed 1%. Incubation mixture contained 10 μM of a studied derivative, 1 mM of NADPH (Sigma-Aldrich) and 0.5 mg/mL of human liver microsomes (HLM, Sigma-Aldrich) in potassium phosphate buffer (0.1 M, pH 7.4). Incubation was carried out in thermostat at 37 °C and started by addition of compound of interest. 50 μL samples were taken after 5, 15, 30, 45 and 60 min. Enzymatic reaction was terminated by the addition of the equal volume of ice-cold acetonitrile containing 10 μM of internal standard (IS). Compound **47** served as IS for compound **19**. Compound **19** served as IS for the remaining compounds. Control incubations were performed without NADPH to assess chemical instability. After collection, samples were immediately centrifuged (10 min, 10,000 rpm) and resulted supernatant was directly analyzed or kept in −80 °C until LC-MS analysis. Natural logarithm of a compound over IS peak area ratio was plotted *vs.* incubation time. Metabolic half-time (t_1/2_) was calculated from the slope of the linear regression, as reported in [43].

LC-MS analysis was performed on an Agilent 1260 system coupled to SingleQuad 6120 mass spectrometer (Agilent Technologies, Santa Clara, CA, USA). Cortex C18+ column (2.1 × 75 mm, 2.7 μm, Waters, Milford, MA, USA) was used in reversed-phase mode with gradient elution starting with 30% of phase A (10 mM ammonium formate in water) and 70% of phase B (10 mM ammonium formate in acetonitrile-water mixture, 95:5 *v*/*v*). The amount of phase B was linearly increased to 100% in 12 min. Total analysis time was 20 min at 40 °C, flow rate was 0.25 mL/min and the injection volume was 10 μL. The mass spectrometer was equipped with electrospray ion source and operated in positive ionization. Mass analyzer was set individually to each compound to detect [M + H]^+^ pseudomolecular ions. MSD parameters of the ESI source were as follows: nebulizer pressure 50 psig (N_2_), drying gas 13 mL/min (N_2_), drying gas temperature 300 °C, capillary voltage 3.5 kV, fragmentor voltage 100 V.

## 4. Conclusions

We have developed a new and facile method for the synthesis of *N*-acylated sulfonamides using *N*-(benzenesulfonyl)cyanamide potassium salts and carboxylic acids. The molecular structures of novel compounds were confirmed by NMR, IR, and also by single crystal X-ray structure analysis for representative compound **22**. The newly synthesized *N*-acylsulfonamides were tested for their *in vitro* cytotoxic activity against the MCF-7, HCT-116 and HeLa cell lines. The most active compounds belonged to a series of 1-naphthyl derivatives. Compound **27** showed outstanding anticancer activity at low and high concentrations against all tested cell lines, especially with regard to the HeLa cell line (GP = 7% at 100 µM, GP = 22% at 10 µM). Compound **47** demonstrated dependent on dilution good cytotoxic activity toward three cell lines (9% ≤ GP ≤ 46% at 100 µM). Performed QSAR analysis demonstrate the importance of some molecular descriptors in the cytotoxic activities against the investigated cell lines. The MCF-7 and HCT-116 QSAR models showed that antitumor activity of *N*-acylsulfonamides depends on distinct topological descriptors and number of fragments C_(Ar)_–CH–C_(Ar)_. On the other hand, QSAR analysis indicated the significance of topological descriptors as well as positive charge at sulfonamide S atom in the prediction of cytotoxic activity against HeLa cell line. Finally, the QSAR studies revealed three predictive and statistically significant models for the investigated compounds, with squared correlation coefficients for training set R^2^ = 0.76‒0.89 and squared correlation coefficients for test set Q^2^ = 0.59‒0.83. *In vitro* tests for metabolic stability in the presence of human liver microsomes and NADPH run on selected compounds showed that chemical nature of both R^1^ and R^2^ substituents of *N*-acylsulfonamides affected their stability. In the studied series of *N*-acylbenzenesulfonamides MLR-based considerations on structure-activity relationships served as a supporting approach beneficial for explanation of biological response. The usage of easily interpretable molecular descriptors is a base to plan further, rational synthesis of potentially more potent derivatives.

## Supplementary Materials

Supplementary materials can be accessed at: http://www.mdpi.com/1420-3049/20/10/19101/s1.
