# Supplementary material for: Synthesis, Molecular Structure, Metabolic Stability and QSAR Studies of a Novel Series of Anticancer N-Acylbenzenesulfonamides
Source: Molecules. 2015 Oct 21;20(10):19101–29. doi: 10.3390/molecules201019101 (PMC6332020; doi:10.3390/molecules201019101)
Supplement: Supplementary file 1 [file molecules-20-19101-s001.pdf]

Supplementary Materials

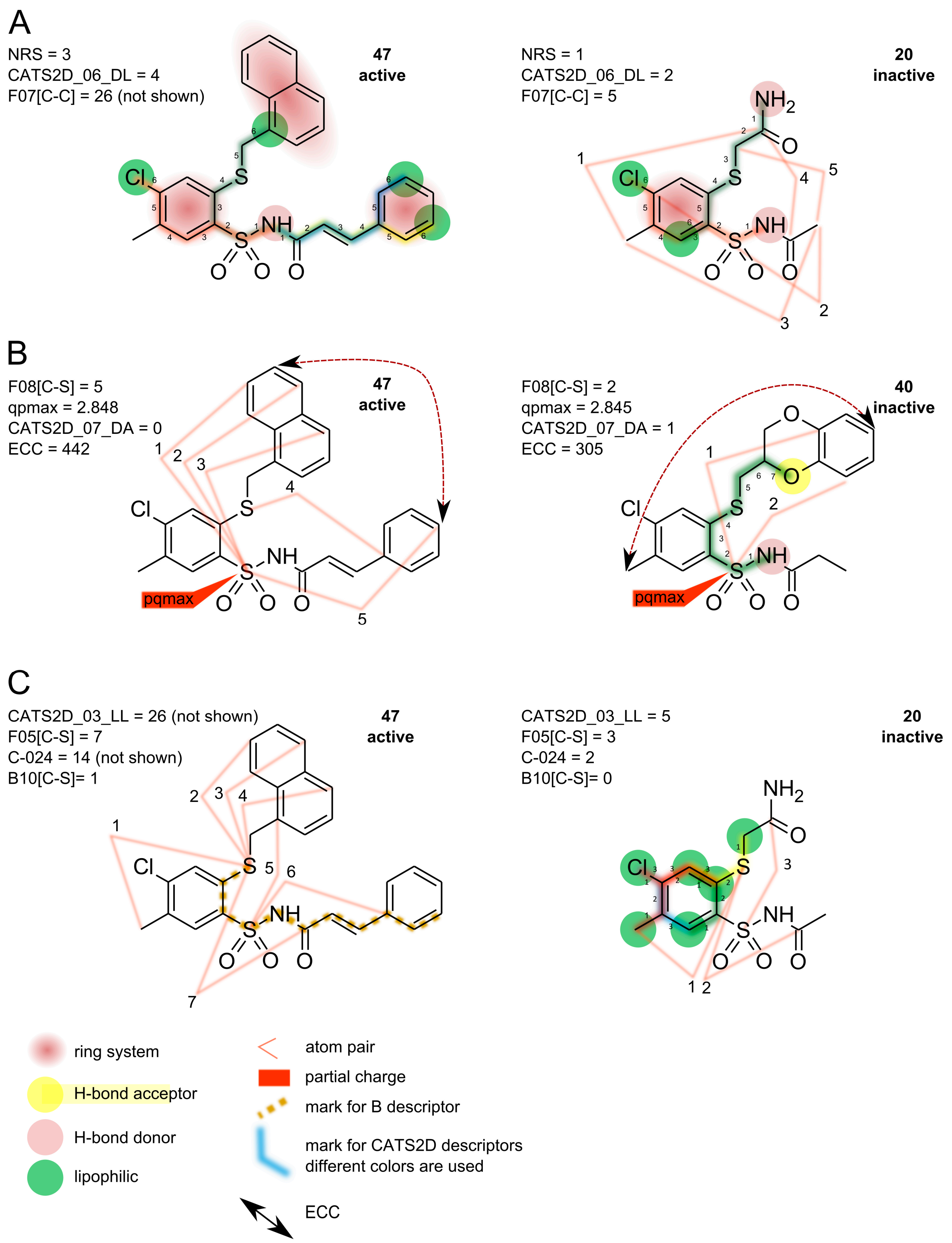

Figure S1. Visualization of molecular descriptors values incorporated in QSAR models for MCF-7 (**A**); HeLa (**B**); HCT-116 (**C**) cell lines.

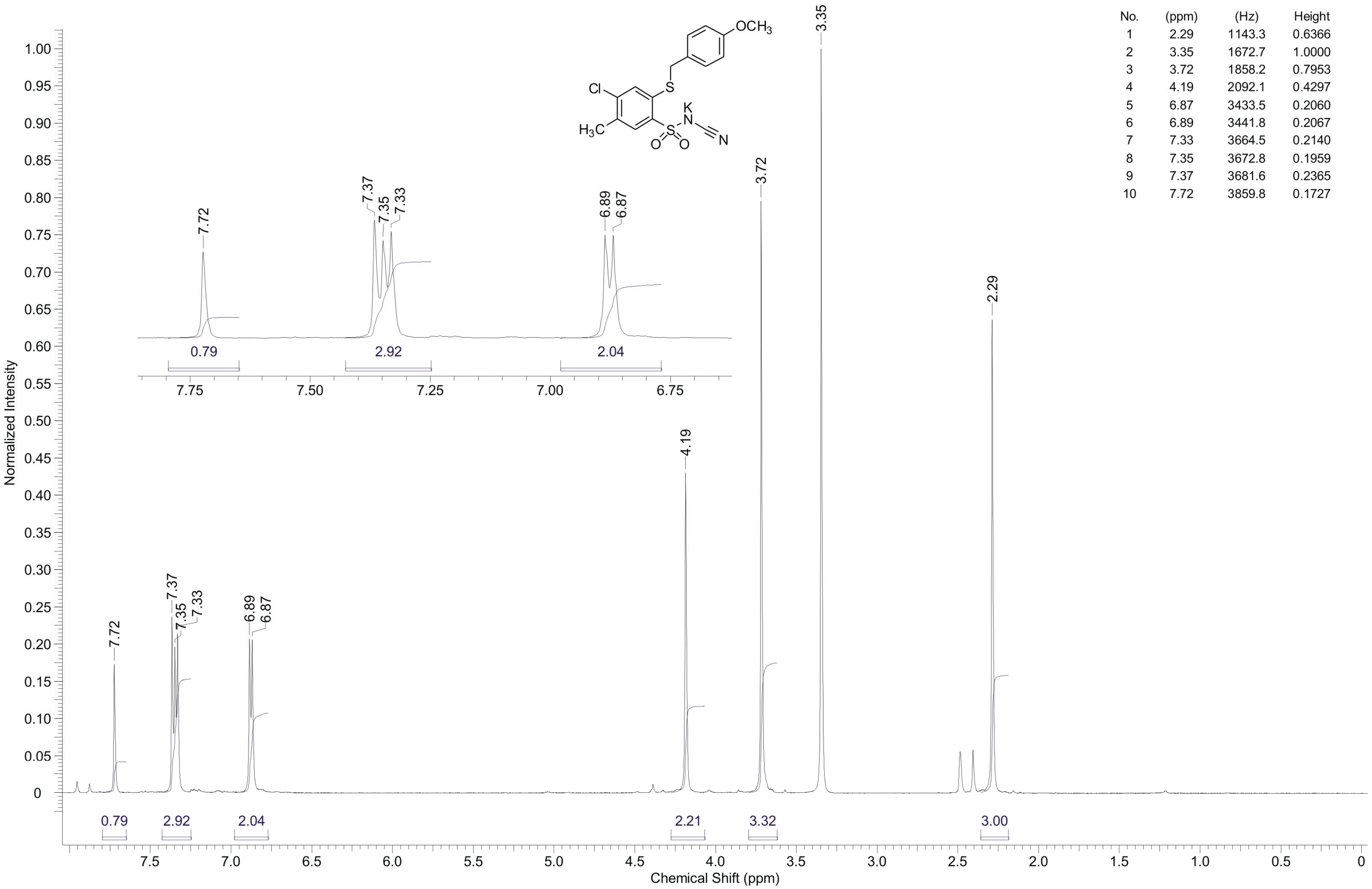

No.	(ppm)	(Hz)	Height
1	2.29	1143.3	0.6366
2	3.35	1672.7	1.0000
3	3.72	1858.2	0.7953
4	4.19	2092.1	0.4297
5	6.87	3433.5	0.2060
6	6.89	3441.8	0.2067
7	7.33	3664.5	0.2140
8	7.35	3672.8	0.1959
9	7.37	3681.6	0.2365
10	7.72	3859.8	0.1727

Figure S2. ^{1}H-NMR of compound **10** (500 MHz, DMSO-d_6).

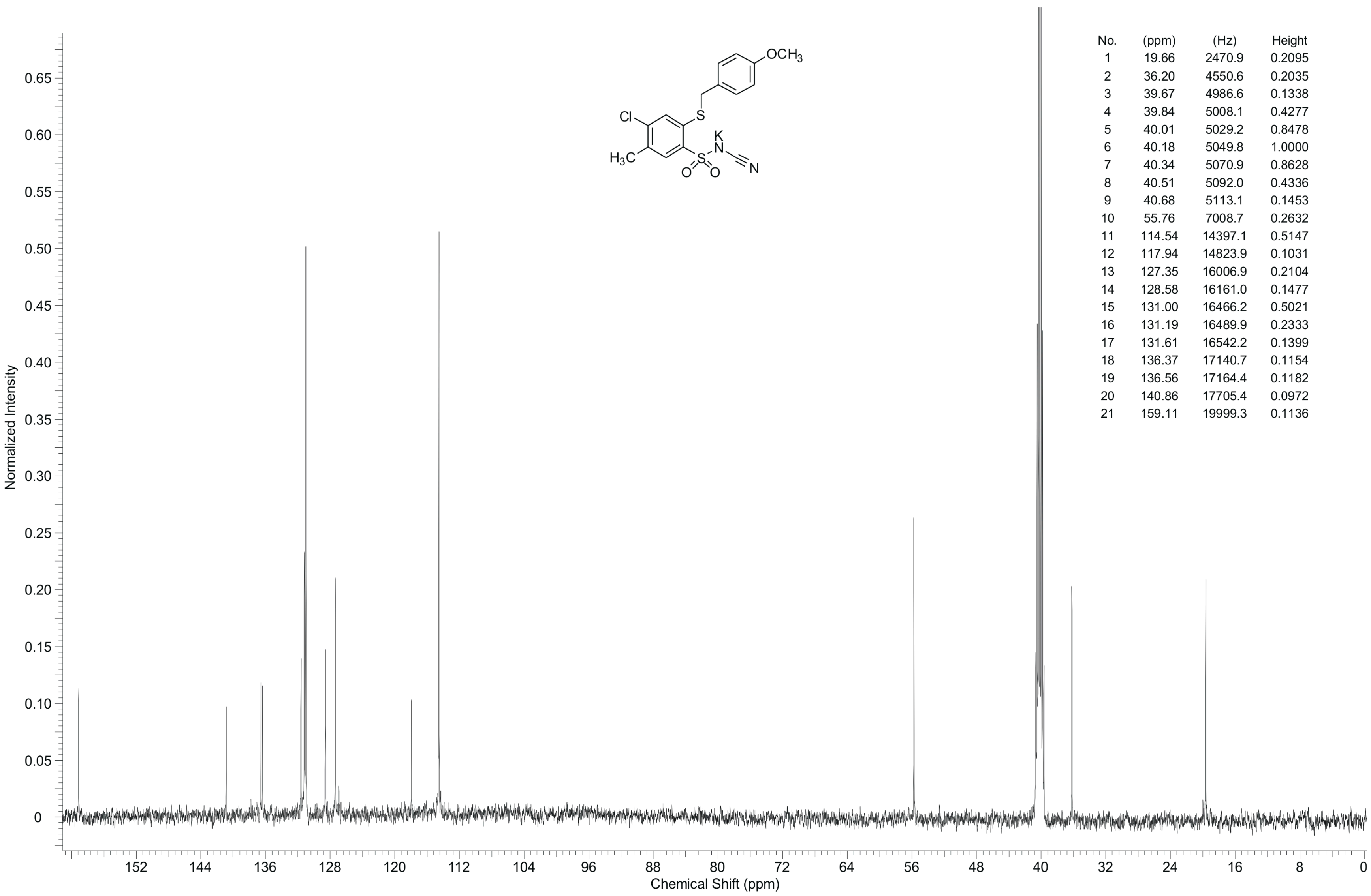

No.	(ppm)	(Hz)	Height
1	19.66	2470.9	0.2095
2	36.20	4550.6	0.2035
3	39.67	4986.6	0.1338
4	39.84	5008.1	0.4277
5	40.01	5029.2	0.8478
6	40.18	5049.8	1.0000
7	40.34	5070.9	0.8628
8	40.51	5092.0	0.4336
9	40.68	5113.1	0.1453
10	55.76	7008.7	0.2632
11	114.54	14397.1	0.5147
12	117.94	14823.9	0.1031
13	127.35	16006.9	0.2104
14	128.58	16161.0	0.1477
15	131.00	16466.2	0.5021
16	131.19	16489.9	0.2333
17	131.61	16542.2	0.1399
18	136.37	17140.7	0.1154
19	136.56	17164.4	0.1182
20	140.86	17705.4	0.0972
21	159.11	19999.3	0.1136

Figure S3. ^{13}C-NMR of compound **10** (125 MHz, DMSO-d_6).

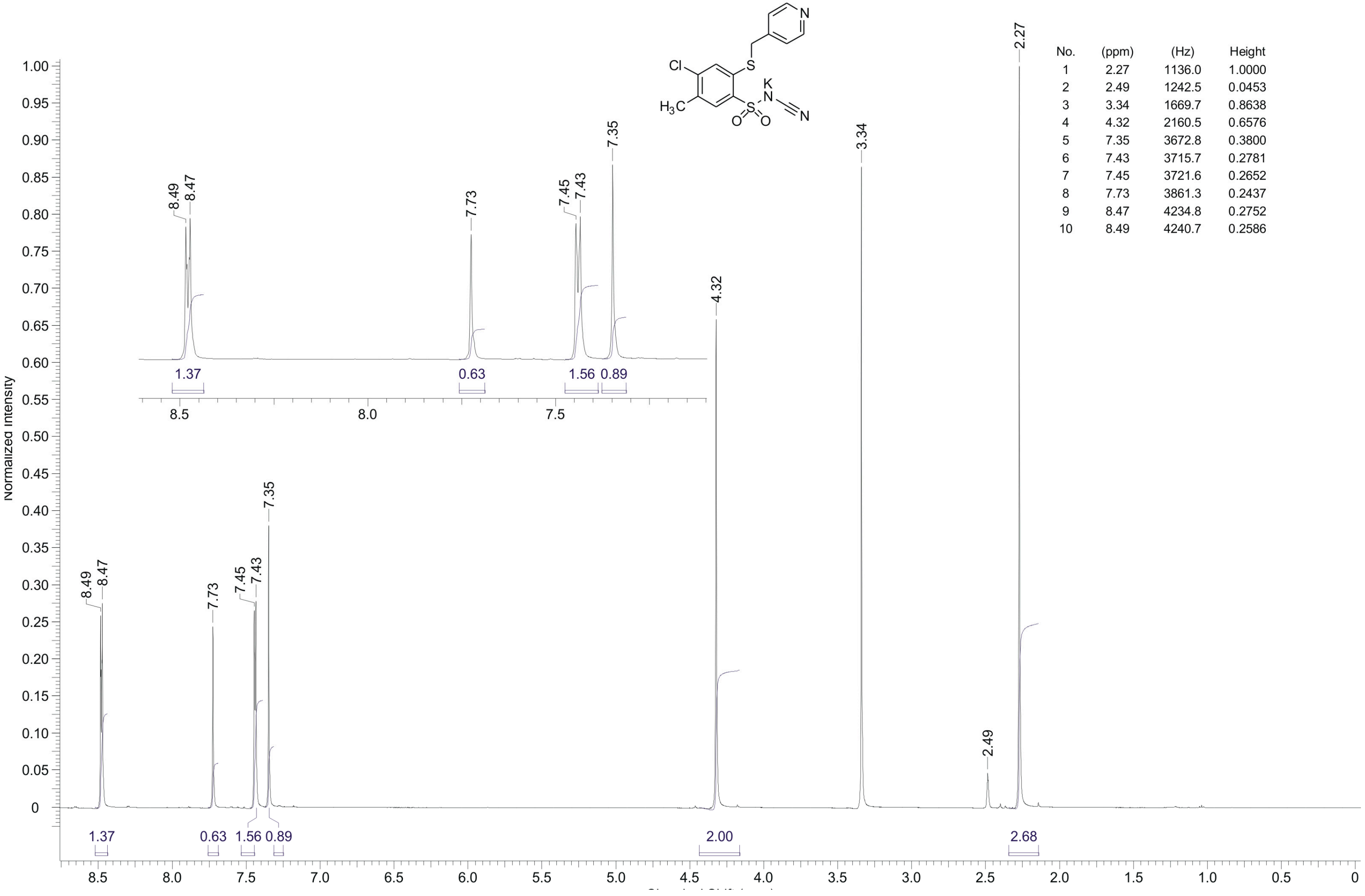

Figure S4. ^{1}H-NMR of compound **11** (500 MHz, DMSO-d_6).

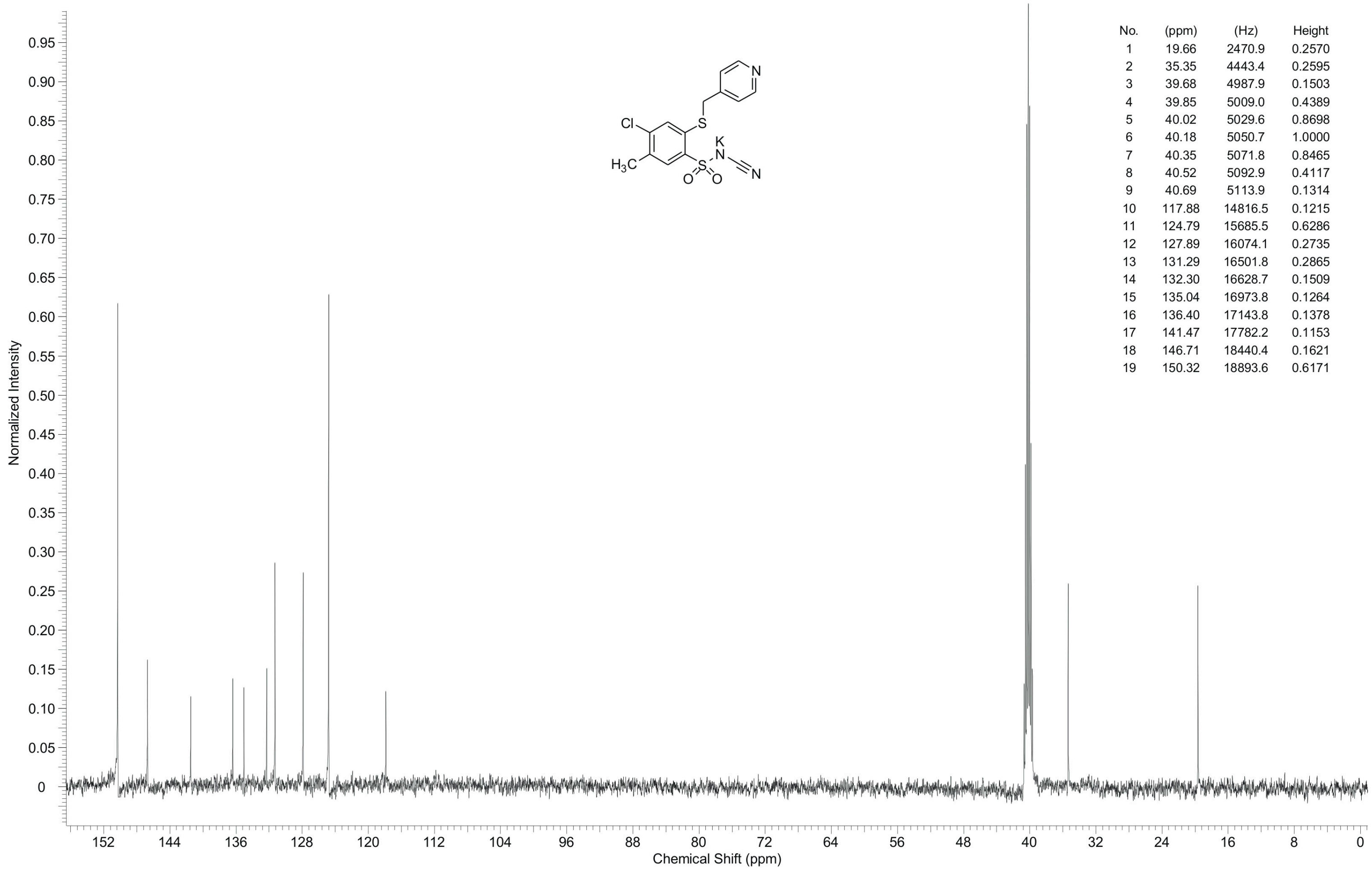

Figure S5. ^{13}C-NMR of compound **11** (125 MHz DMSO-d_6).

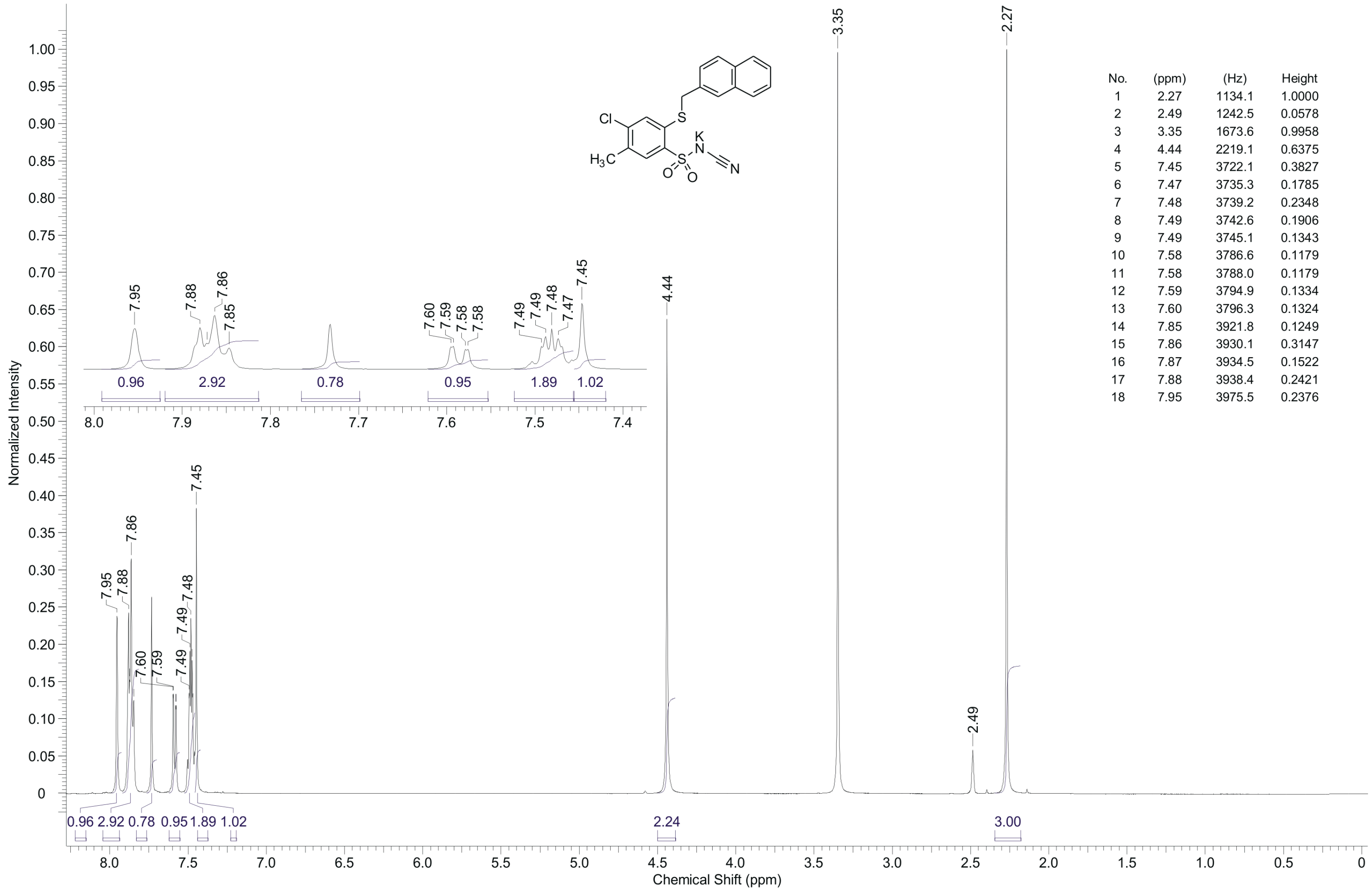

No.	(ppm)	(Hz)	Height
1	2.27	1134.1	1.0000
2	2.49	1242.5	0.0578
3	3.35	1673.6	0.9958
4	4.44	2219.1	0.6375
5	7.45	3722.1	0.3827
6	7.47	3735.3	0.1785
7	7.48	3739.2	0.2348
8	7.49	3742.6	0.1906
9	7.49	3745.1	0.1343
10	7.58	3786.6	0.1179
11	7.58	3788.0	0.1179
12	7.59	3794.9	0.1334
13	7.60	3796.3	0.1324
14	7.85	3921.8	0.1249
15	7.86	3930.1	0.3147
16	7.87	3934.5	0.1522
17	7.88	3938.4	0.2421
18	7.95	3975.5	0.2376

Figure S6. ^{1}H-NMR of compound **13** (500 MHz, DMSO-d_6).

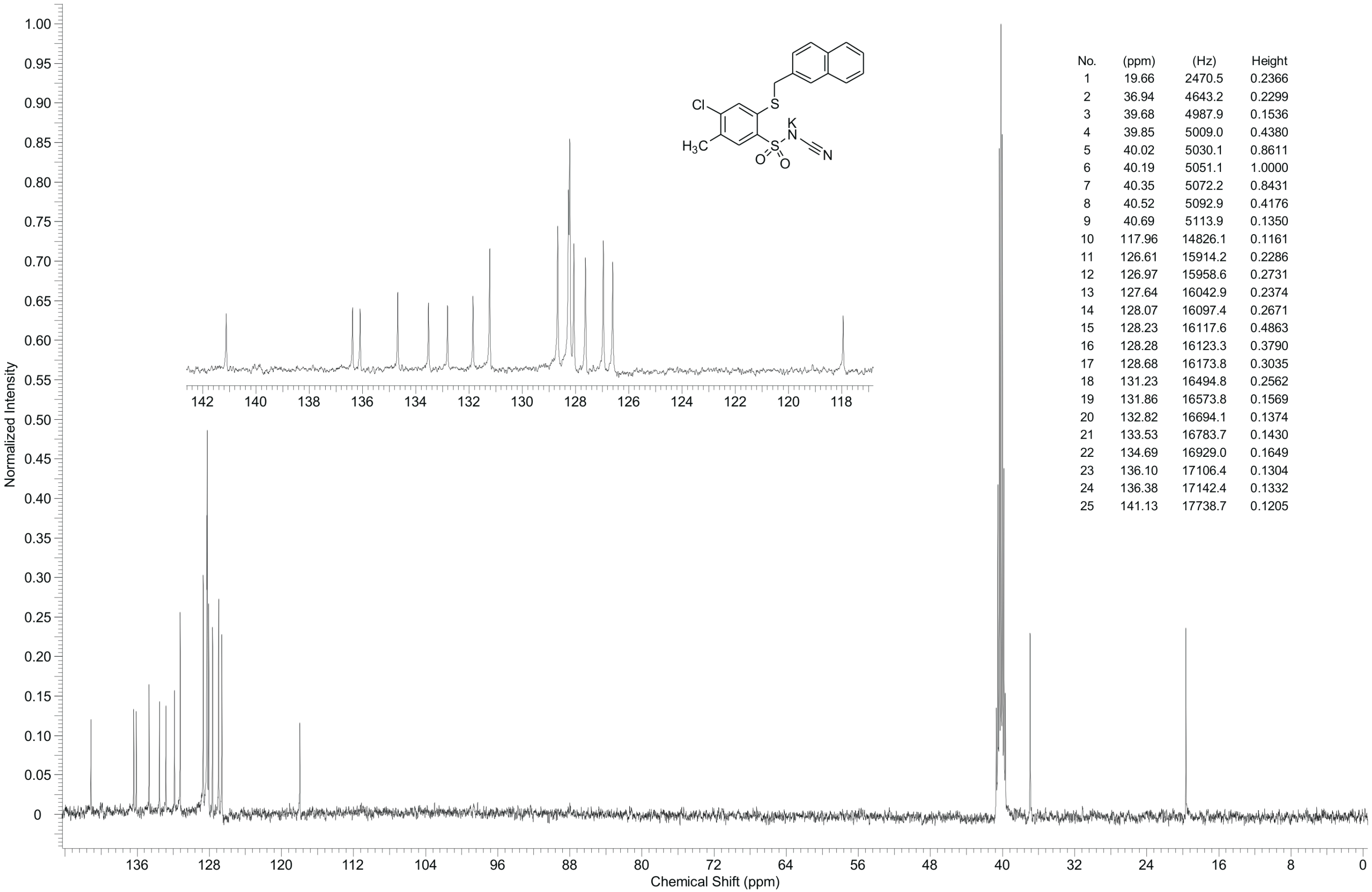

No.	(ppm)	(Hz)	Height
1	19.66	2470.5	0.2366
2	36.94	4643.2	0.2299
3	39.68	4987.9	0.1536
4	39.85	5009.0	0.4380
5	40.02	5030.1	0.8611
6	40.19	5051.1	1.0000
7	40.35	5072.2	0.8431
8	40.52	5092.9	0.4176
9	40.69	5113.9	0.1350
10	117.96	14826.1	0.1161
11	126.61	15914.2	0.2286
12	126.97	15958.6	0.2731
13	127.64	16042.9	0.2374
14	128.07	16097.4	0.2671
15	128.23	16117.6	0.4863
16	128.28	16123.3	0.3790
17	128.68	16173.8	0.3035
18	131.23	16494.8	0.2562
19	131.86	16573.8	0.1569
20	132.82	16694.1	0.1374
21	133.53	16783.7	0.1430
22	134.69	16929.0	0.1649
23	136.10	17106.4	0.1304
24	136.38	17142.4	0.1332
25	141.13	17738.7	0.1205

Figure S7. ^{13}C-NMR of compound **13** (125 MHz, DMSO-d_6).

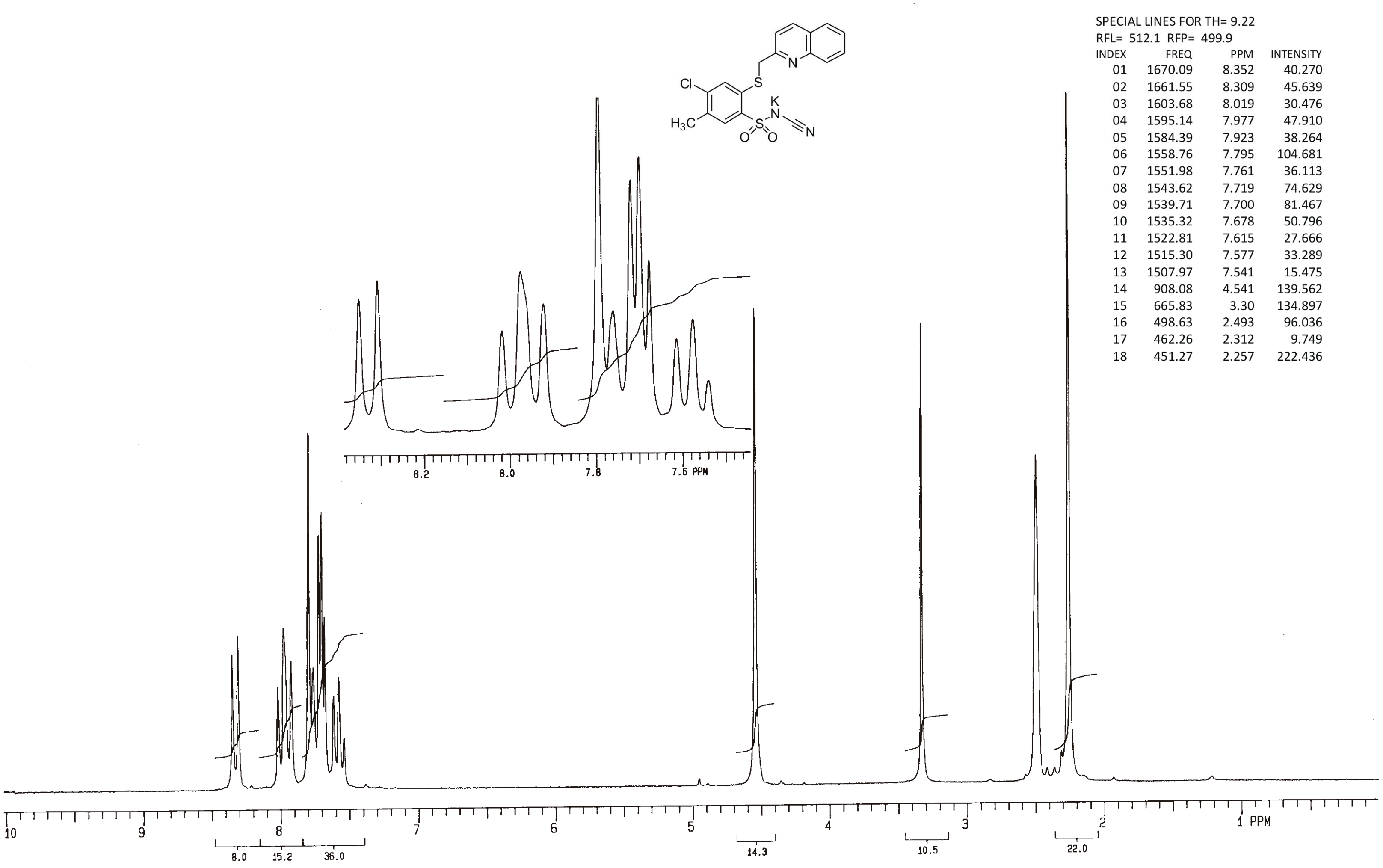

INDEX	FREQ	PPM	INTENSITY
01	1670.09	8.352	40.270
02	1661.55	8.309	45.639
03	1603.68	8.019	30.476
04	1595.14	7.977	47.910
05	1584.39	7.923	38.264
06	1558.76	7.795	104.681
07	1551.98	7.761	36.113
08	1543.62	7.719	74.629
09	1539.71	7.700	81.467
10	1535.32	7.678	50.796
11	1522.81	7.615	27.666
12	1515.30	7.577	33.289
13	1507.97	7.541	15.475
14	908.08	4.541	139.562
15	665.83	3.30	134.897
16	498.63	2.493	96.036
17	462.26	2.312	9.749
18	451.27	2.257	222.436

Figure S8. ^{1}H-NMR of compound **14** (200 MHz, DMSO-d_6).

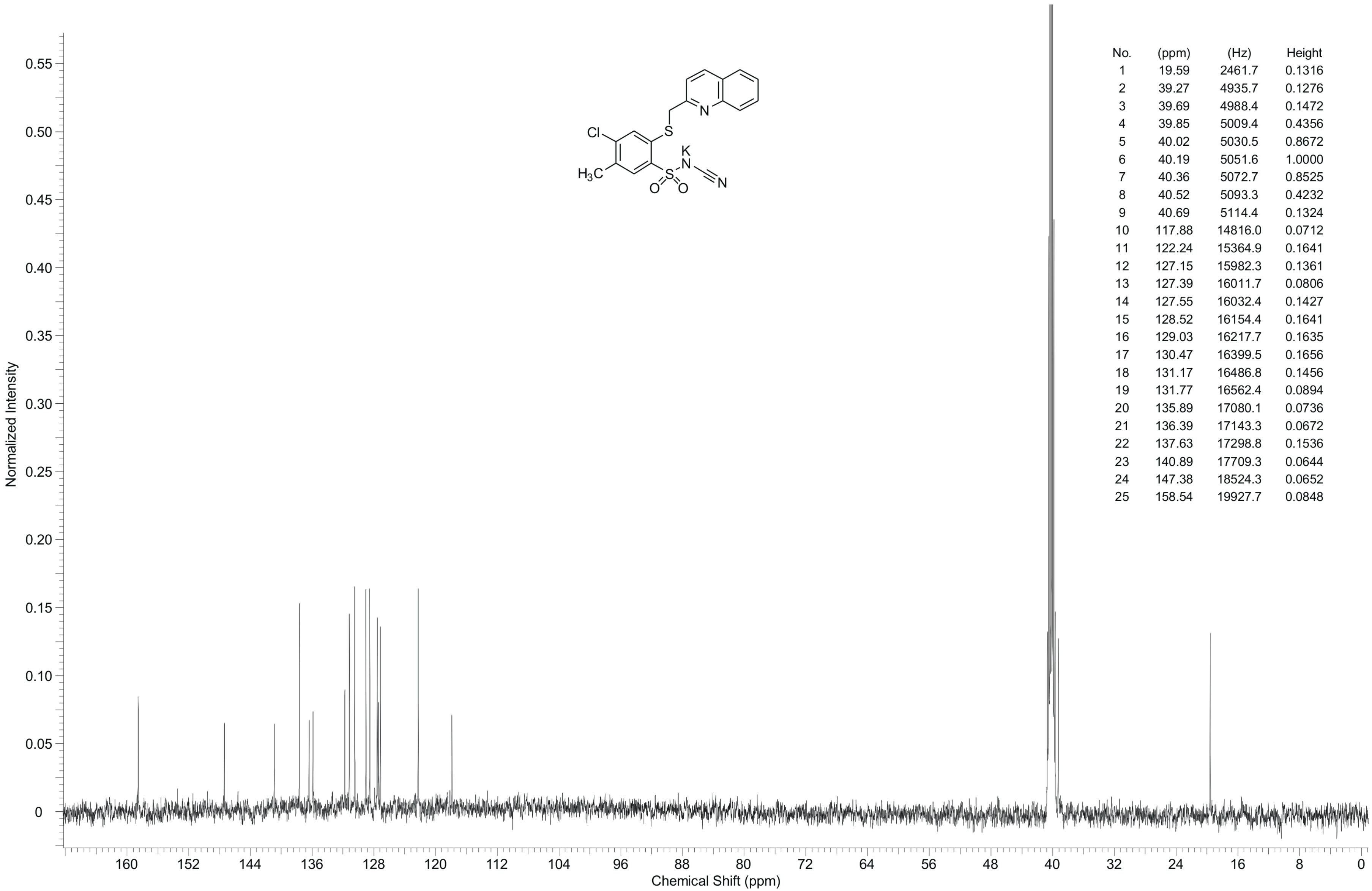

No.	(ppm)	(Hz)	Height
1	19.59	2461.7	0.1316
2	39.27	4935.7	0.1276
3	39.69	4988.4	0.1472
4	39.85	5009.4	0.4356
5	40.02	5030.5	0.8672
6	40.19	5051.6	1.0000
7	40.36	5072.7	0.8525
8	40.52	5093.3	0.4232
9	40.69	5114.4	0.1324
10	117.88	14816.0	0.0712
11	122.24	15364.9	0.1641
12	127.15	15982.3	0.1361
13	127.39	16011.7	0.0806
14	127.55	16032.4	0.1427
15	128.52	16154.4	0.1641
16	129.03	16217.7	0.1635
17	130.47	16399.5	0.1656
18	131.17	16486.8	0.1456
19	131.77	16562.4	0.0894
20	135.89	17080.1	0.0736
21	136.39	17143.3	0.0672
22	137.63	17298.8	0.1536
23	140.89	17709.3	0.0644
24	147.38	18524.3	0.0652
25	158.54	19927.7	0.0848

Figure S9. ^{13}C-NMR of compound **14** (125 MHz, DMSO-d_6).

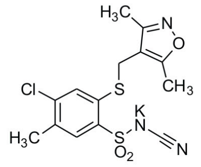

SPECIAL LINES FOR TH= 8.68
RFL= 672.9 RFP= 499.9

INDEX	FREQ	PPM	INTENSITY
01	1550.45	7.753	32.382
02	1488.26	7.442	37.974
03	816.84	4.085	56.081
04	670.86	3.355	118.881
05	501.26	2.507	11.008
06	499.55	2.498	8.808
07	468.42	2.342	119.903
08	465.49	2.328	98.374
09	448.92	2.245	118.690

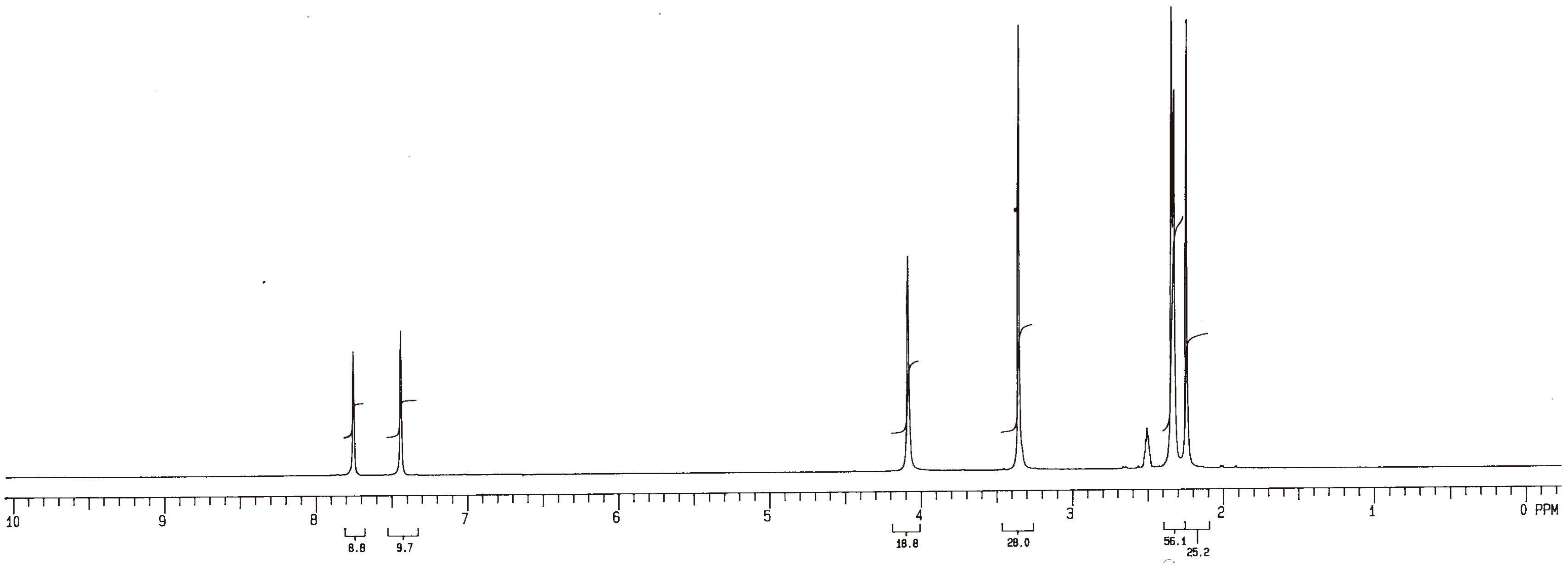

Figure S10. ^{1}H-NMR of compound **17** (200 MHz, DMSO-*d*6).

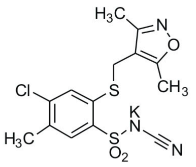

SPECIAL LINES FOR TH= 7.38
RFL= 2095.7 RFP= 0

INDEX	FREQ	PPM	INTENSITY
01	8387.9	166.792	18.808
02	8039.1	159.857	16.375
03	7138.0	141.939	14.780
04	6828.6	135.786	20.378
05	6764.5	134.512	18.800
06	6653.1	132.296	25.203
07	6576.4	130.772	37.004
08	6493.8	129.128	33.368
09	5907.1	117.462	10.414
10	5502.0	109.406	24.048
11	2063.5	41.033	18.155
12	2042.5	40.615	55.677
13	2021.5	40.196	110.598
14	2014.1	40.051	9.210
15	2000.4	39.778	130.052
16	1993.1	39.632	8.555
17	1979.5	39.361	109.362
18	1958.5	38.945	54.254
19	1937.4	38.524	18.625
20	1245.0	24.757	26.845
21	970.0	19.288	39.223
22	545.9	10.854	32.677
23	502.4	9.991	36.168

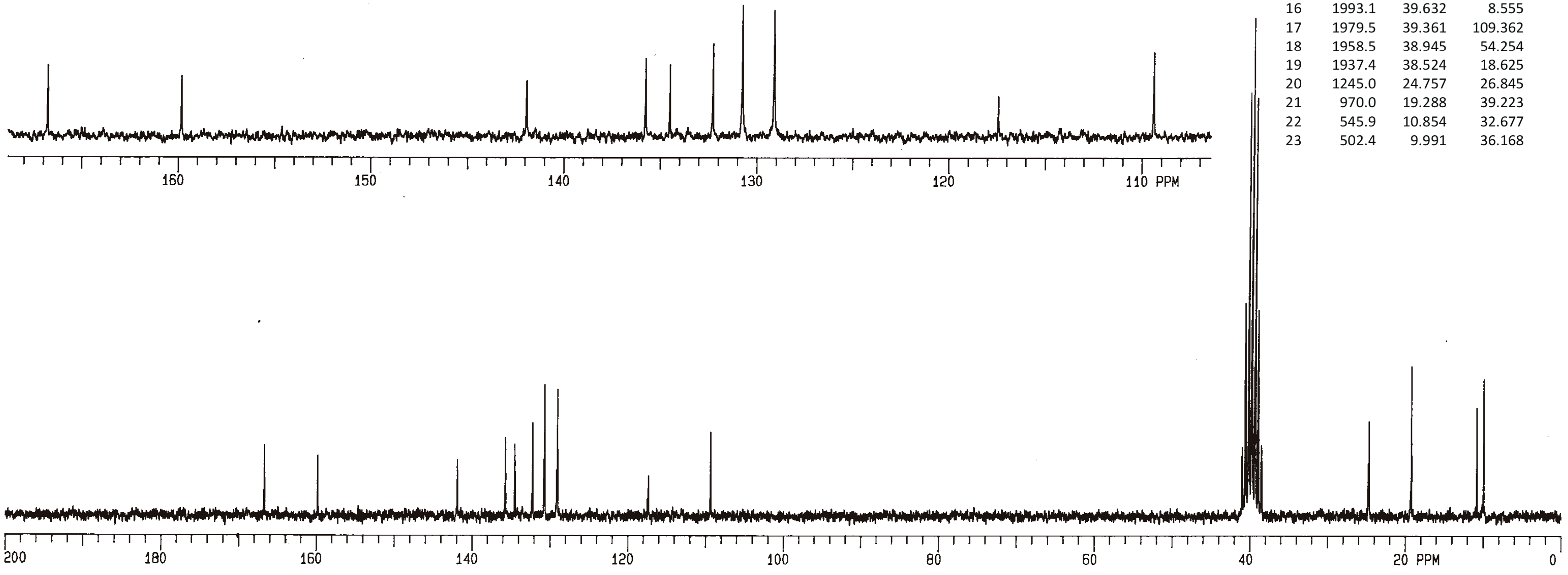

Figure S11. ^{13}C-NMR of compound **17** (50 MHz, DMSO-d_6).

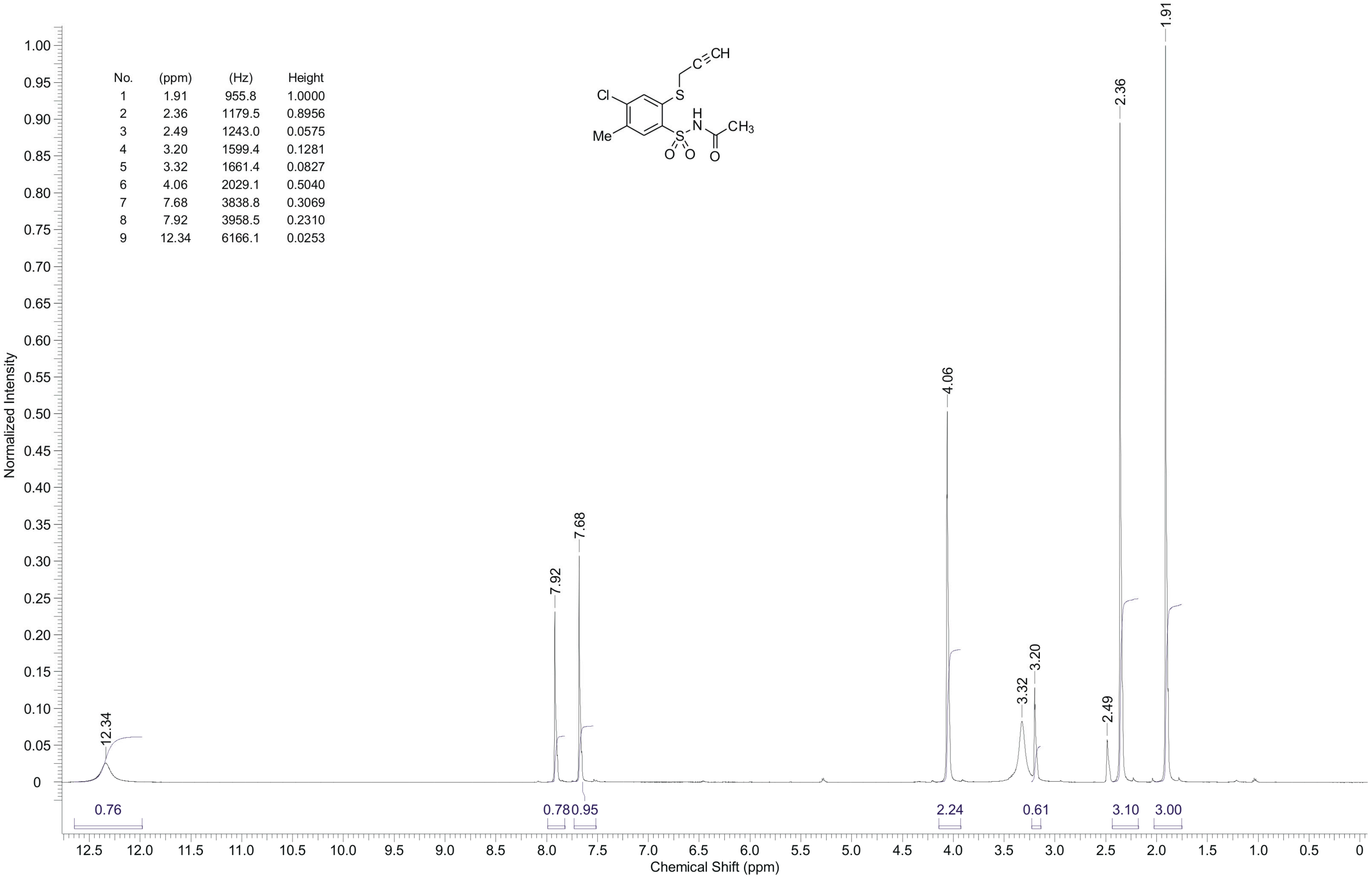

Figure S12. ^{1}H-NMR of compound **19** (500 MHz, DMSO-d_6).

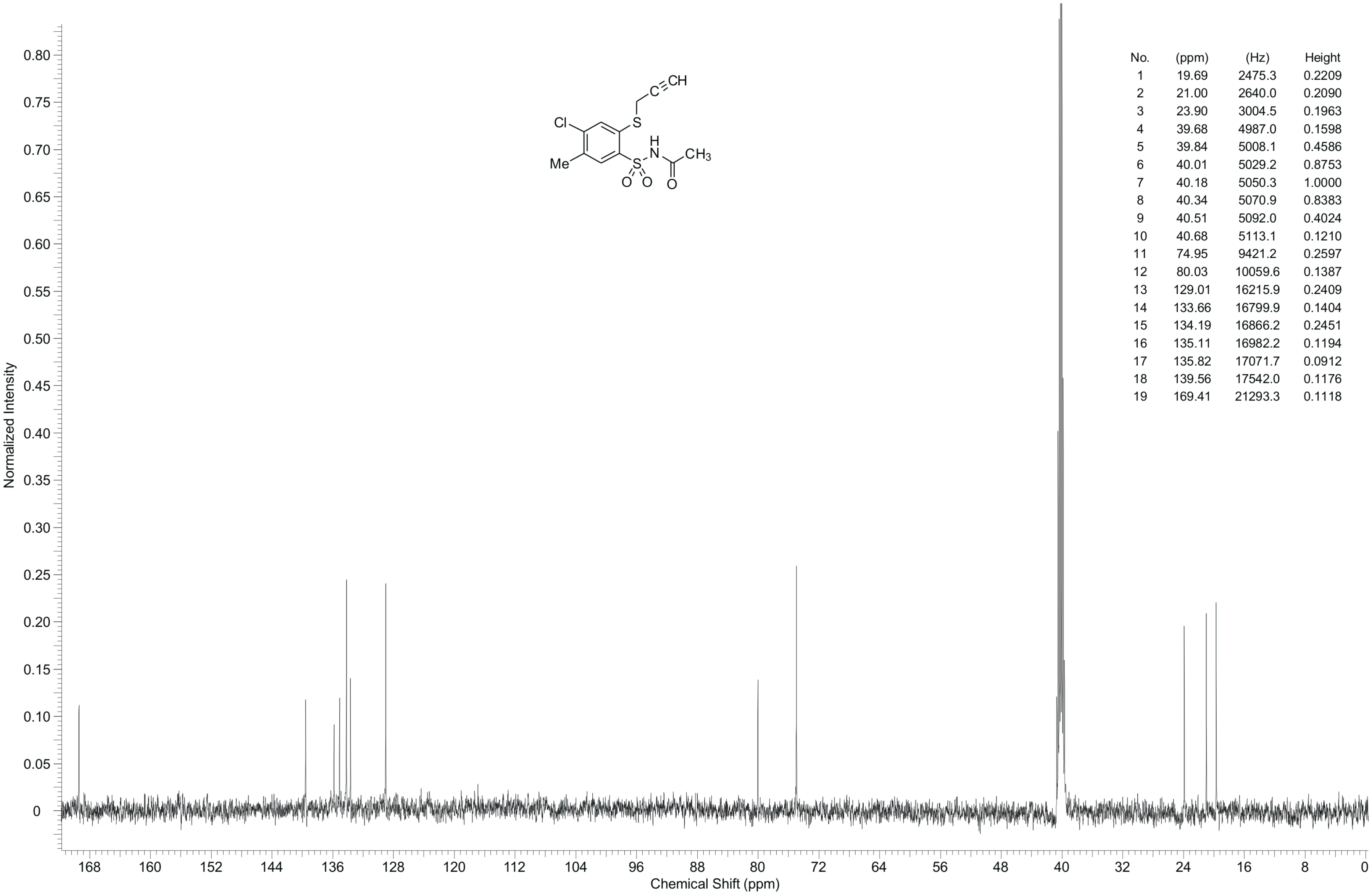

No.	(ppm)	(Hz)	Height
1	19.69	2475.3	0.2209
2	21.00	2640.0	0.2090
3	23.90	3004.5	0.1963
4	39.68	4987.0	0.1598
5	39.84	5008.1	0.4586
6	40.01	5029.2	0.8753
7	40.18	5050.3	1.0000
8	40.34	5070.9	0.8383
9	40.51	5092.0	0.4024
10	40.68	5113.1	0.1210
11	74.95	9421.2	0.2597
12	80.03	10059.6	0.1387
13	129.01	16215.9	0.2409
14	133.66	16799.9	0.1404
15	134.19	16866.2	0.2451
16	135.11	16982.2	0.1194
17	135.82	17071.7	0.0912
18	139.56	17542.0	0.1176
19	169.41	21293.3	0.1118

Figure S13. ^{13}C-NMR of compound **19** (125 MHz, DMSO-*d*$_6$).

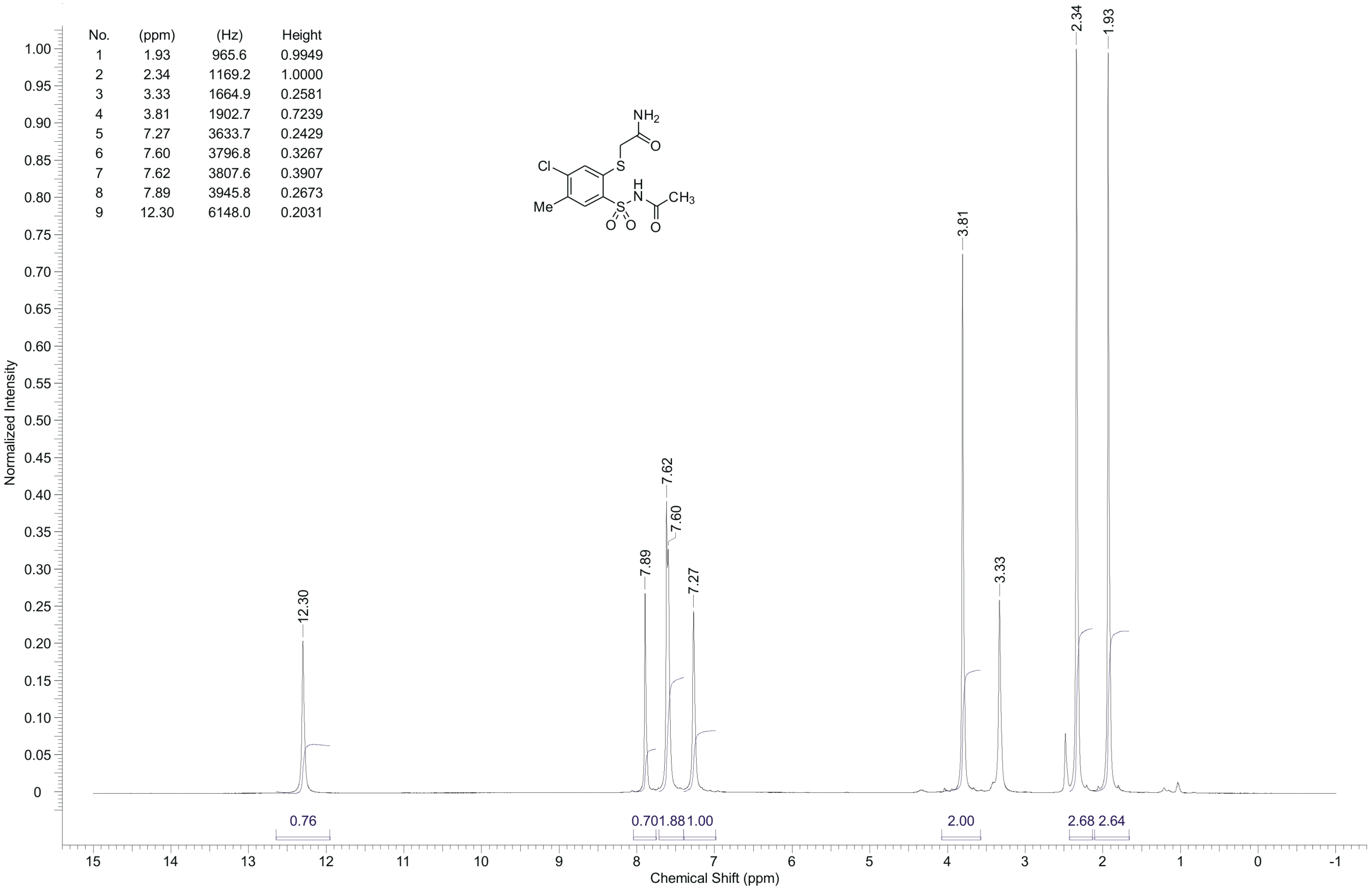

Figure S14. ^{1}H-NMR of compound **20** (500 MHz, DMSO-d_6).

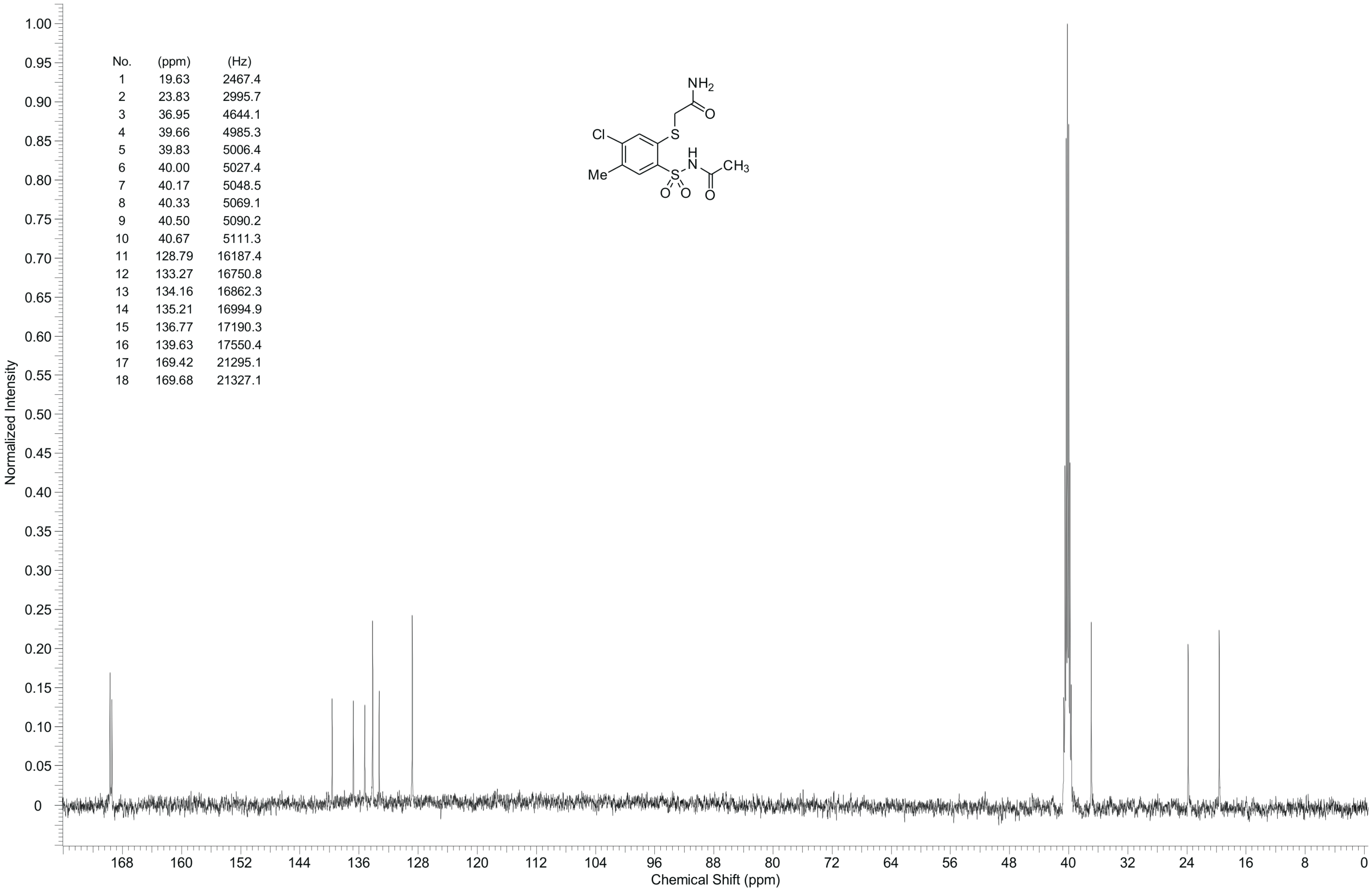

No.	(ppm)	(Hz)
1	19.63	2467.4
2	23.83	2995.7
3	36.95	4644.1
4	39.66	4985.3
5	39.83	5006.4
6	40.00	5027.4
7	40.17	5048.5
8	40.33	5069.1
9	40.50	5090.2
10	40.67	5111.3
11	128.79	16187.4
12	133.27	16750.8
13	134.16	16862.3
14	135.21	16994.9
15	136.77	17190.3
16	139.63	17550.4
17	169.42	21295.1
18	169.68	21327.1

Figure S15. ^{13}C-NMR of compound **20** (125 MHz, DMSO-d_6).

SPECIAL LINES FOR TH= 5.37
RFL= 672.9 RFP= 499.9

INDEX	FREQ	PPM	INTENSITY
01	2472.45	12.364	5.490
02	2470.82	12.356	5.626
03	2469.35	12.348	5.874
04	2468.32	12.343	5.600
05	1580.29	7.902	41.338
06	1533.67	7.669	44.529
07	1492.91	7.465	19.775
08	1485.38	7.428	36.345
09	1474.95	7.376	14.527
10	1468.74	7.345	35.502
11	1461.14	7.307	32.693
12	1459.87	7.300	27.348
13	1452.91	7.265	12.463
14	888.96	4.445	67.031
15	673.28	3.367	203.335
16	664.01	3.320	6.679
17	504.86	2.525	21.792
18	503.29	2.517	29.110
19	501.67	2.509	23.586
20	470.13	2.351	111.958
21	385.71	1.929	137.331

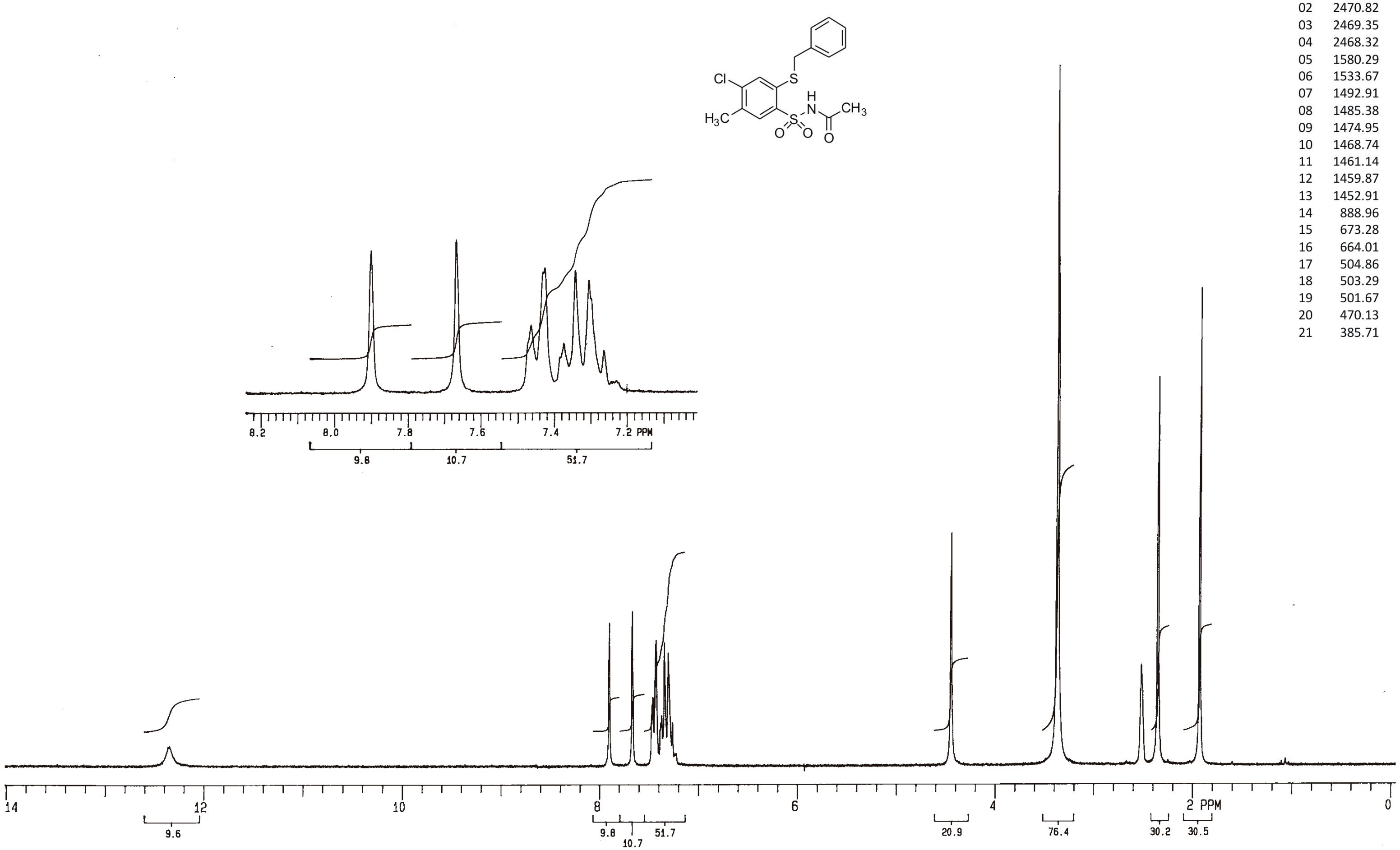

Figure S16. ^{1}H-NMR of compound **21** (200 MHz, DMSO-d_6).

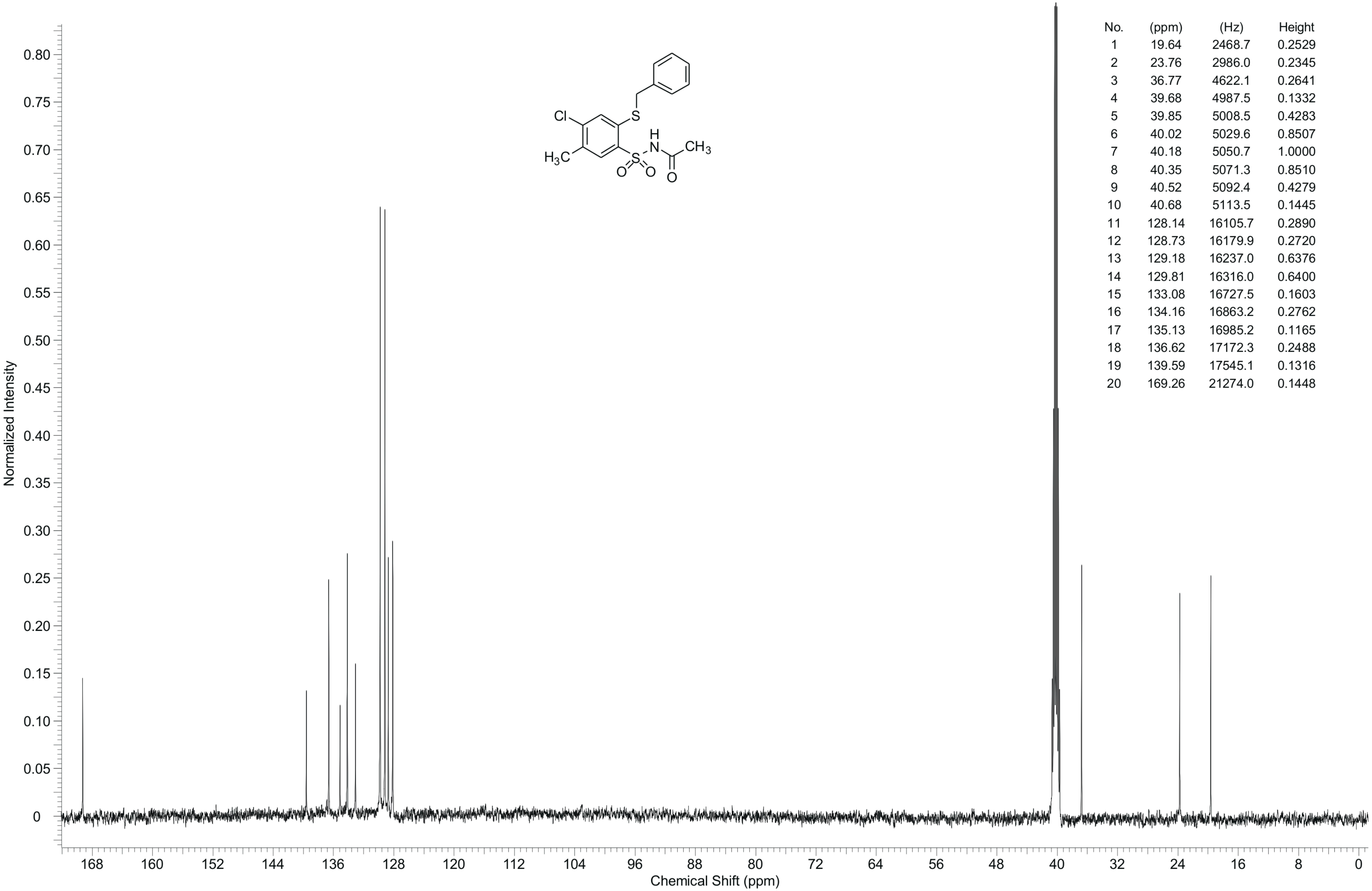

No.	(ppm)	(Hz)	Height
1	19.64	2468.7	0.2529
2	23.76	2986.0	0.2345
3	36.77	4622.1	0.2641
4	39.68	4987.5	0.1332
5	39.85	5008.5	0.4283
6	40.02	5029.6	0.8507
7	40.18	5050.7	1.0000
8	40.35	5071.3	0.8510
9	40.52	5092.4	0.4279
10	40.68	5113.5	0.1445
11	128.14	16105.7	0.2890
12	128.73	16179.9	0.2720
13	129.18	16237.0	0.6376
14	129.81	16316.0	0.6400
15	133.08	16727.5	0.1603
16	134.16	16863.2	0.2762
17	135.13	16985.2	0.1165
18	136.62	17172.3	0.2488
19	139.59	17545.1	0.1316
20	169.26	21274.0	0.1448

Figure S17. ^{13}C-NMR of compound **21** (125 MHz, DMSO-d_6).

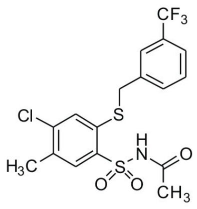

SPECIAL LINES FOR TH= 5.80

RFL= 672.9 RFP= 499.9

INDEX	FREQ	PPM	INTENSITY
01	2474.02	12.372	33.429
02	1582.09	7.911	43.269
03	1563.64	7.819	42.607
04	1553.20	7.767	22.515
05	1540.68	7.704	50.319
06	1523.98	7.621	47.450
07	1515.17	7.577	22.658
08	914.93	4.575	71.642
09	675.65	3.379	17.677
10	503.34	2.517	30.295
11	469.72	2.349	117.663
12	384.04	1.920	122.906

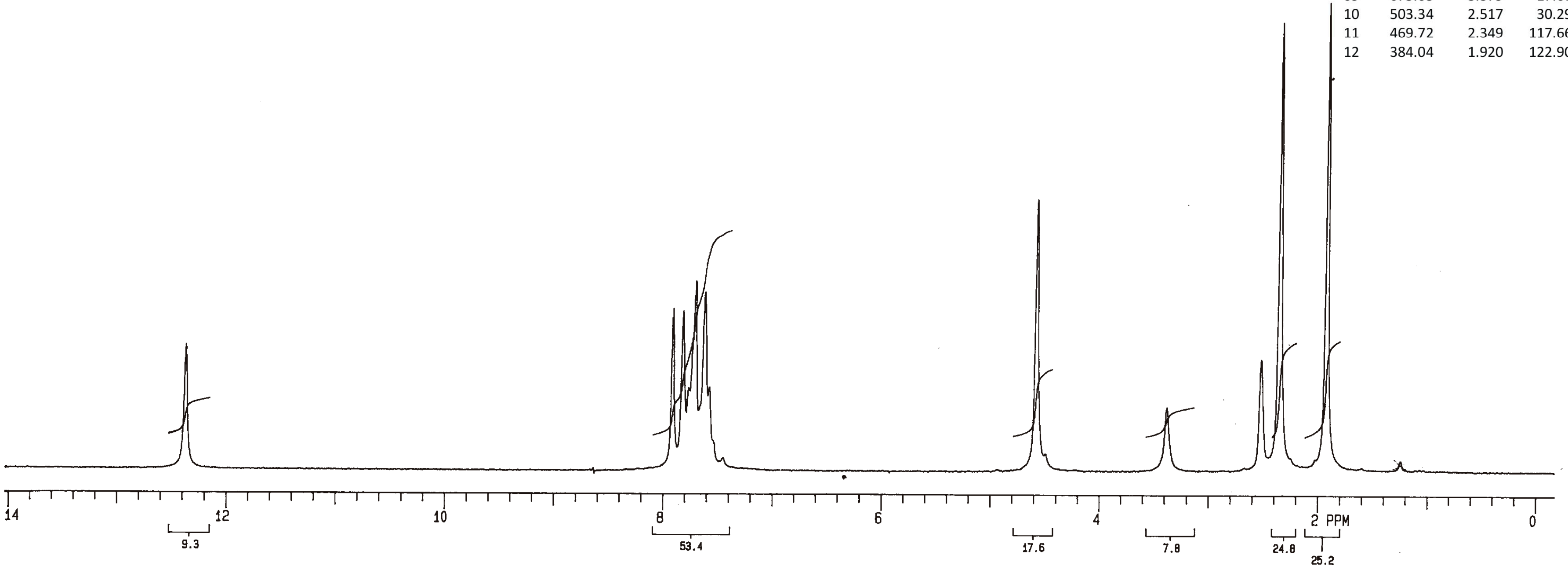

Figure S18. ^{1}H-NMR of compound **22** (200 MHz, DMSO-d_6).

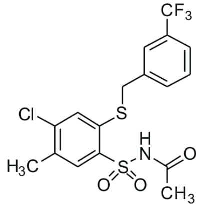

SPECIAL LINES FOR TH= 4.75
RFL= 2095.7 RFP= 0

INDEX	FREQ	PPM	INTENSITY
01	8489.7	168.816	26.137
02	7000.8	139.209	21.305
03	6945.5	138.111	27.604
04	6805.1	135.319	19.908
05	6794.4	135.107	15.559
06	6728.7	133.799	31.983
07	6706.7	133.362	25.254
08	6694.7	133.123	25.194
09	6531.0	129.868	36.778
10	6524.0	129.729	6.854
11	6492.1	129.098	7.001
12	6475.5	128.764	28.089
13	6342.9	126.128	15.838
14	63339.2	126.055	15.178
15	6335.4	125.978	5.084
16	6261.4	124.508	13.684
17	6257.7	124.433	13.269
18	6253.9	124.358	5.698
19	2064.3	41.048	18.181
20	2043.0	40.625	54.823
21	2035.7	40.479	8.012
22	2022.2	40.211	110.486
23	2014.1	40.051	12.275
24	2001.1	39.792	130.362
25	1980.2	39.375	111.364
26	1959.2	38.959	56.256
27	1938.2	38.540	18.866
28	1793.6	35.665	12.412
29	1168.8	23.241	30.125
30	966.5	19.219	33.671

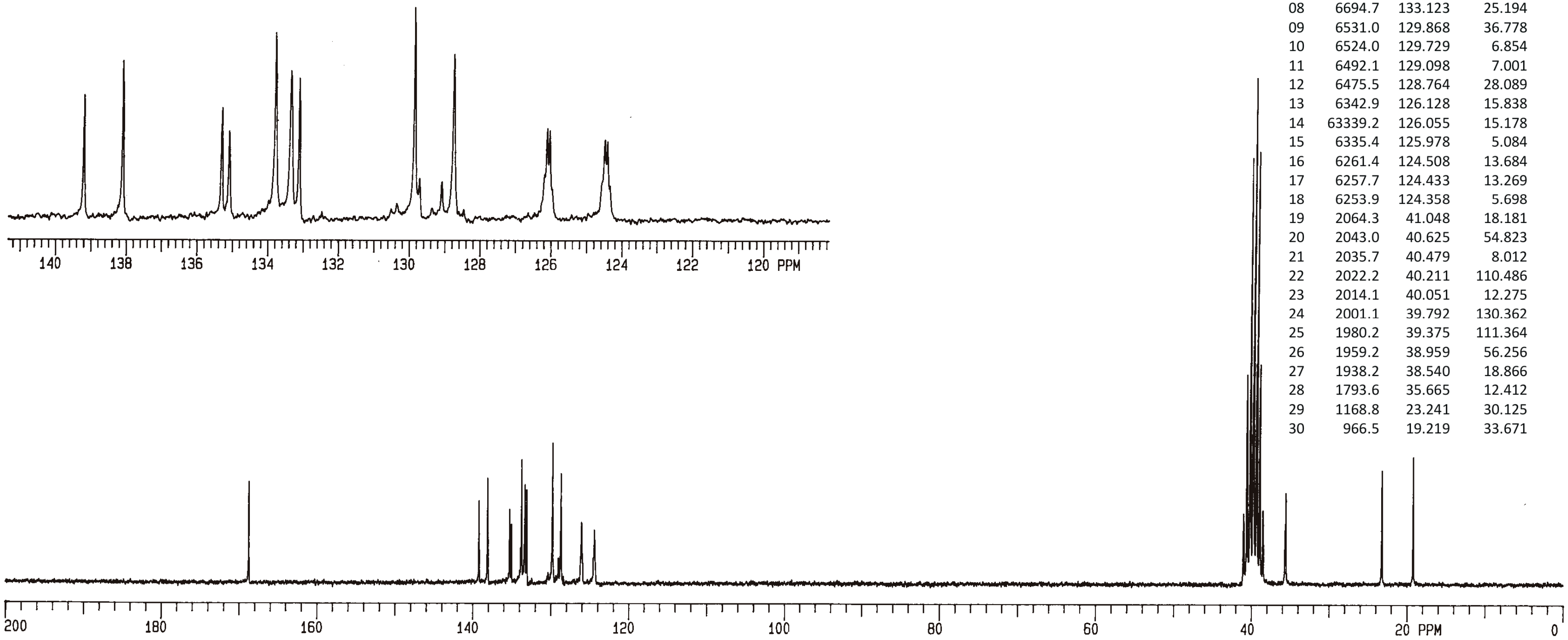

Figure S19. ^{13}C-NMR of compound **22** (50 MHz, DMSO-d_6).

SPECIAL LINES FOR TH= 9.35
RFL= 672.9 RFP= 499.9

INDEX	FREQ	PPM	INTENSITY
01	2468.32	12.346	26.951
02	1581.45	7.808	51.920
03	1535.20	7.676	54.794
04	1496.50	7.489	29.862
05	1488.22	7.442	108.825
06	1484.33	7.423	95.347
07	1477.83	7.390	17.035
08	1475.93	7.381	19.267
09	891.51	4.458	85.462
10	671.94	3.360	67.694
11	504.40	2.522	30.415
12	502.88	2.515	31.683
13	470.77	2.354	139.716
14	385.25	1.923	159.619

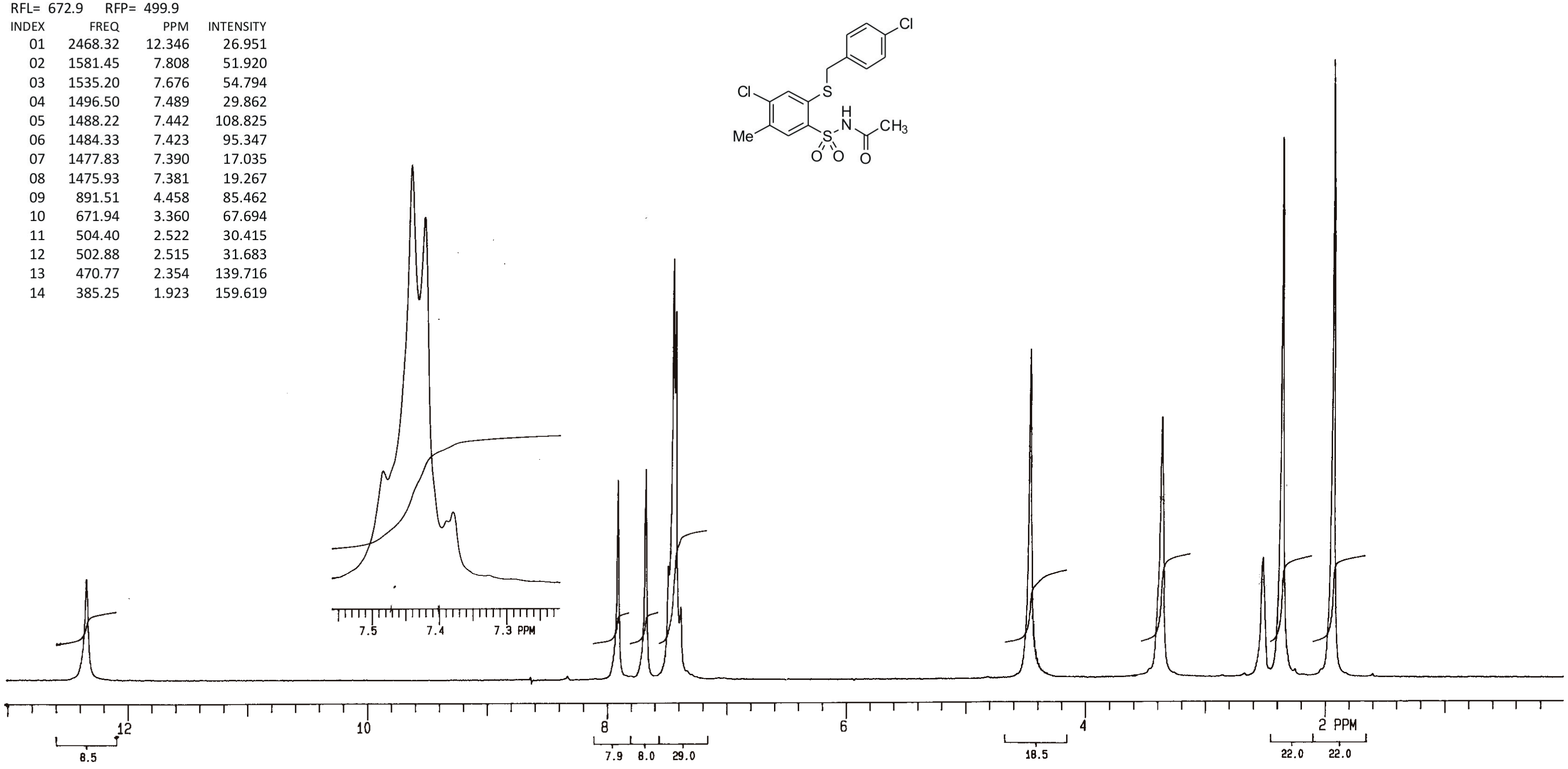

Figure S20. ^{1}H-NMR of compound **24** (200 MHz, DMSO-d_6).

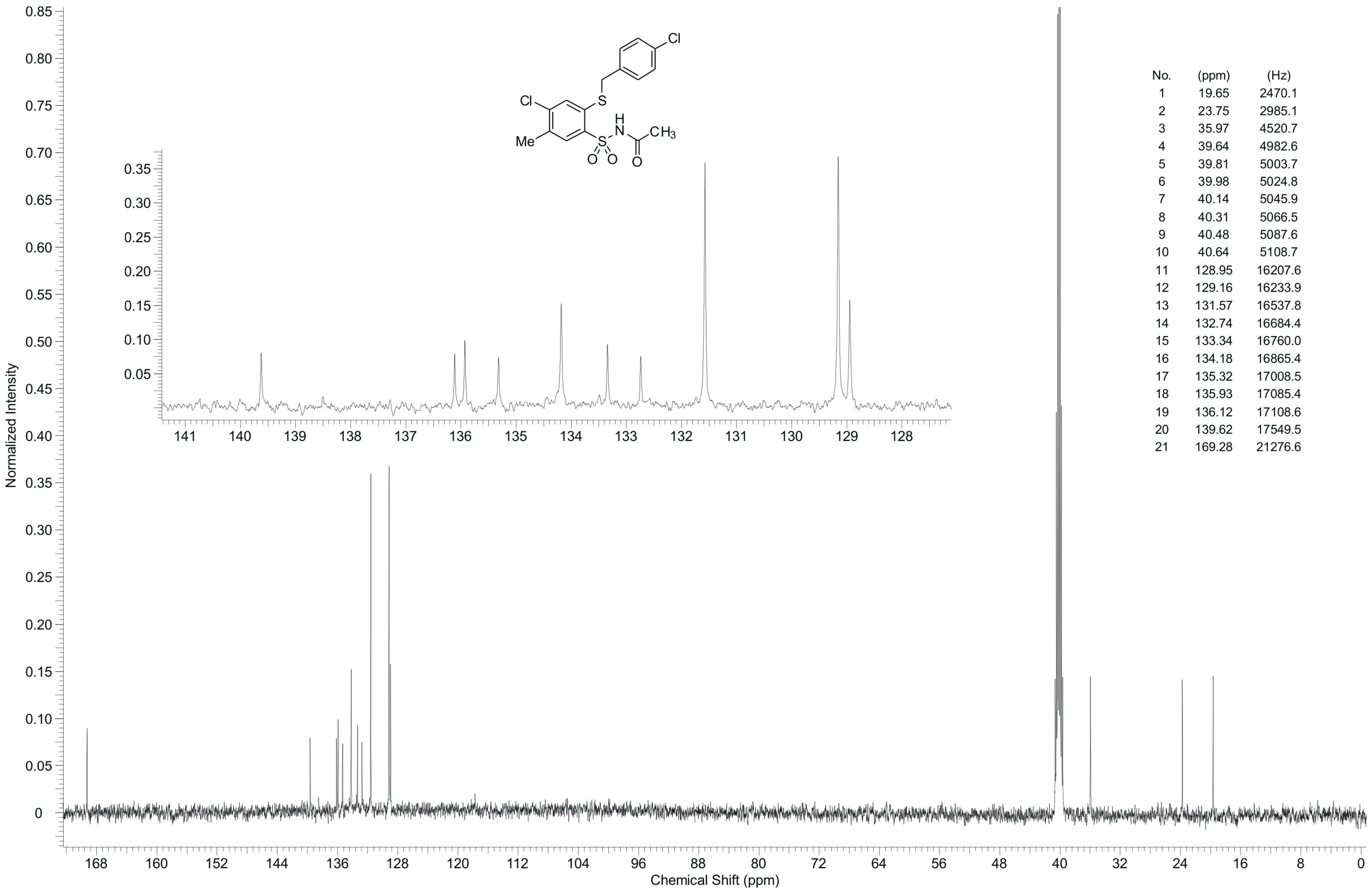

Figure S21. ^{13}C-NMR of compound **24** (125 MHz, DMSO-d_6).

SPECIAL LINES FOR TH= 4.04
RFL= 672.9 RFP= 499.9

INDEX	FREQ	PPM	INTENSITY
01	2461.78	12.310	25.620
02	1582.84	7.915	36.722
03	1533.90	7.670	39.035
04	1490.59	7.454	21.193
05	1483.99	7.421	37.685
06	1473.37	7.368	16.889
07	1467.40	7.338	36.040
08	1459.92	7.301	38.603
09	1452.97	7.266	13.257
10	1447.92	7.240	5.441
11	888.55	4.443	61.971
12	672.36	3.362	38.196
13	503.34	2.517	36.909
14	471.52	2.358	100.658
15	457.84	2.289	12.276
16	450.42	2.252	30.265
17	443.00	2.215	30.772
18	435.57	2.178	11.754
19	400.79	2.004	6.583
20	249.63	1.248	4.221
21	237.52	1.188	4.107
22	185.57	0.928	32.100
23	178.21	0.891	60.336
24	170.78	0.854	28.544

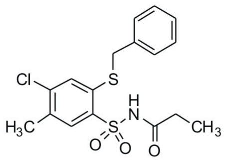

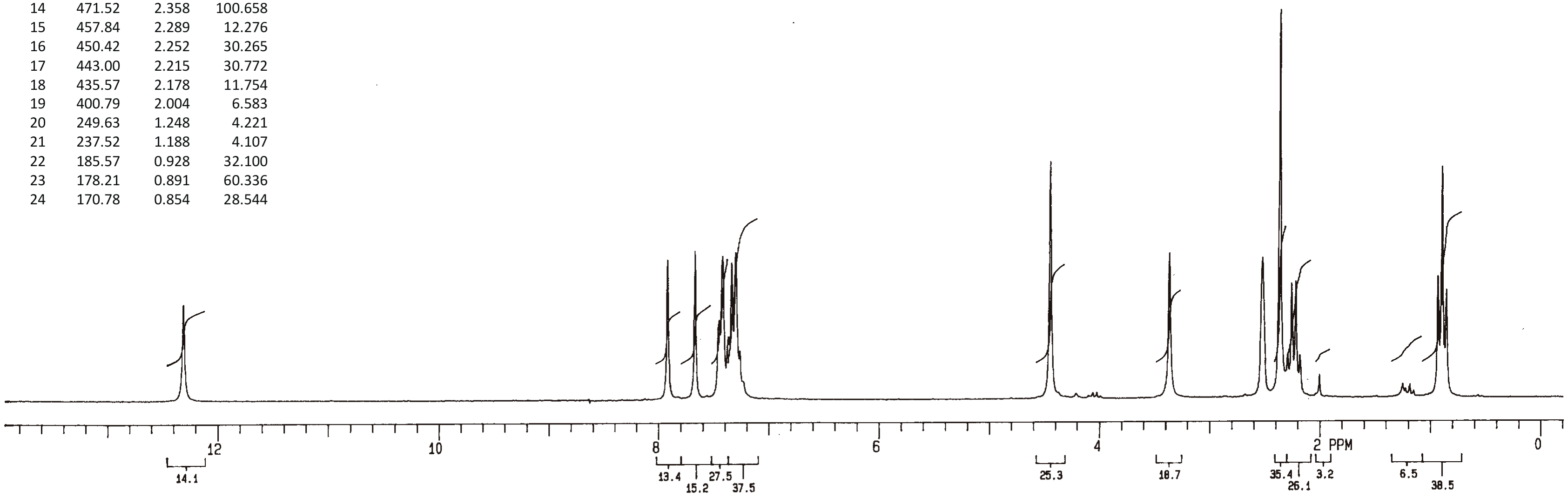

Figure S22. ^{1}H-NMR of compound **33** (200 MHz, DMSO-d_6).

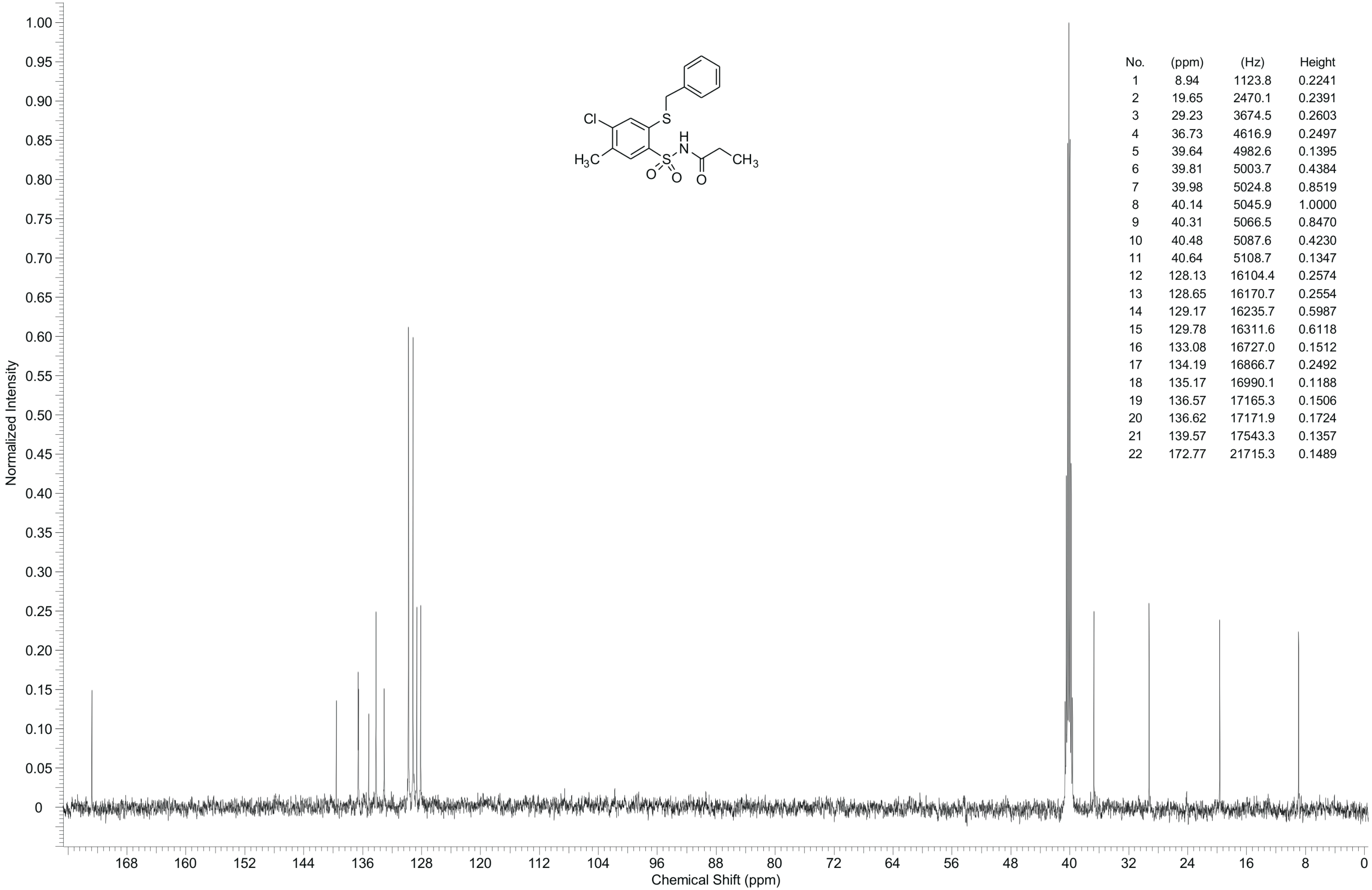

Figure S23. ^{13}C-NMR of compound **33** (125 MHz, DMSO-d_6).

SPECIAL LINES FOR TH= 4.04
RFL= 672.9 RFP= 499.9

INDEX	FREQ	PPM	INTENSITY
01	2453.94	12.271	37.393
02	1581.16	7.907	43.672
03	1531.30	7.657	44.954
04	1473.79	7.359	50.301
05	1464.74	7.325	57.055
06	1380.89	6.905	51.189
07	1373.65	6.869	46.027
08	873.71	4.369	76.249
09	746.28	3.732	139.120
10	670.68	3.351	88.837
11	501.49	2.508	77.017
12	471.11	2.356	124.565
13	455.47	2.278	23.265
14	448.51	2.243	41.904
15	441.32	2.207	42.421
16	434.47	2.173	21.431
17	183.31	0.917	44.163
18	176.94	0.885	73.982
19	169.99	0.850	42.362

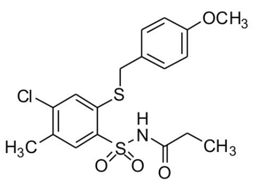

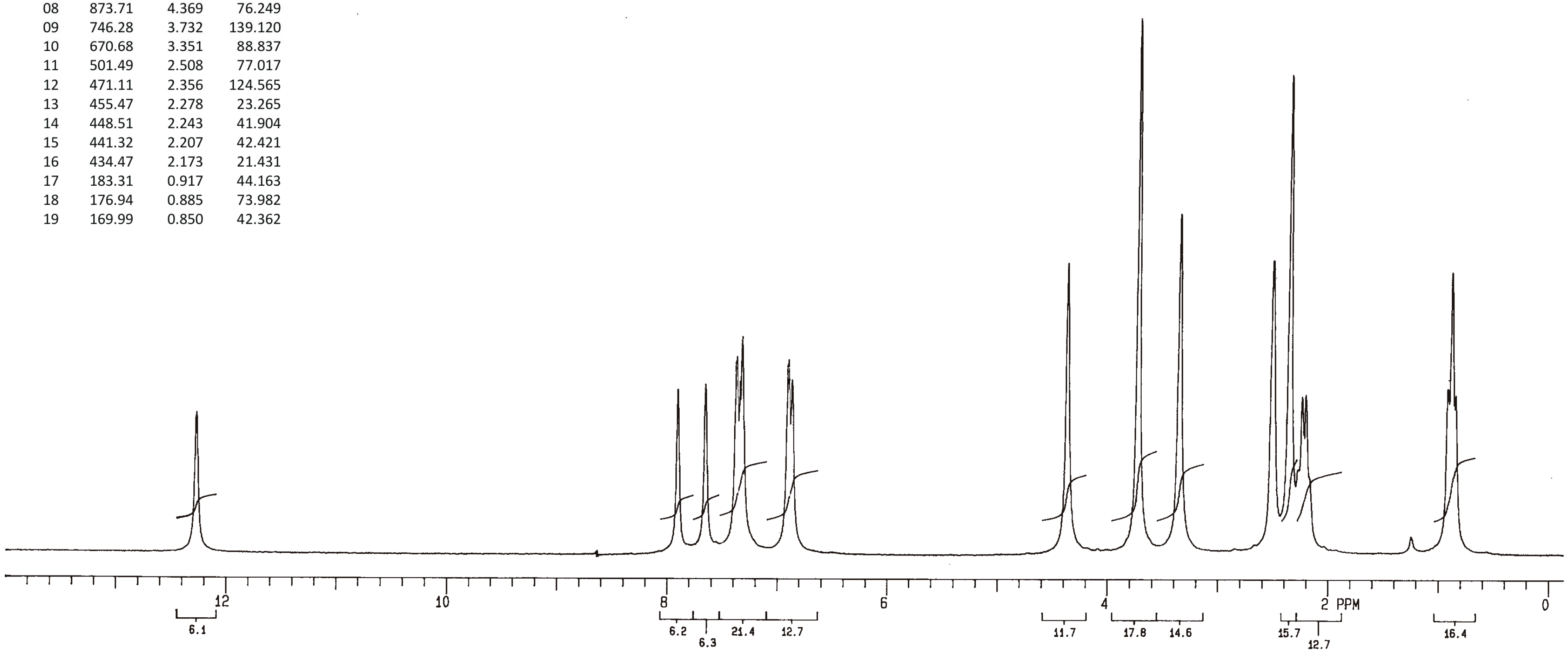

Figure S24. ^{1}H-NMR of compound **37** (200 MHz, DMSO-d_6).

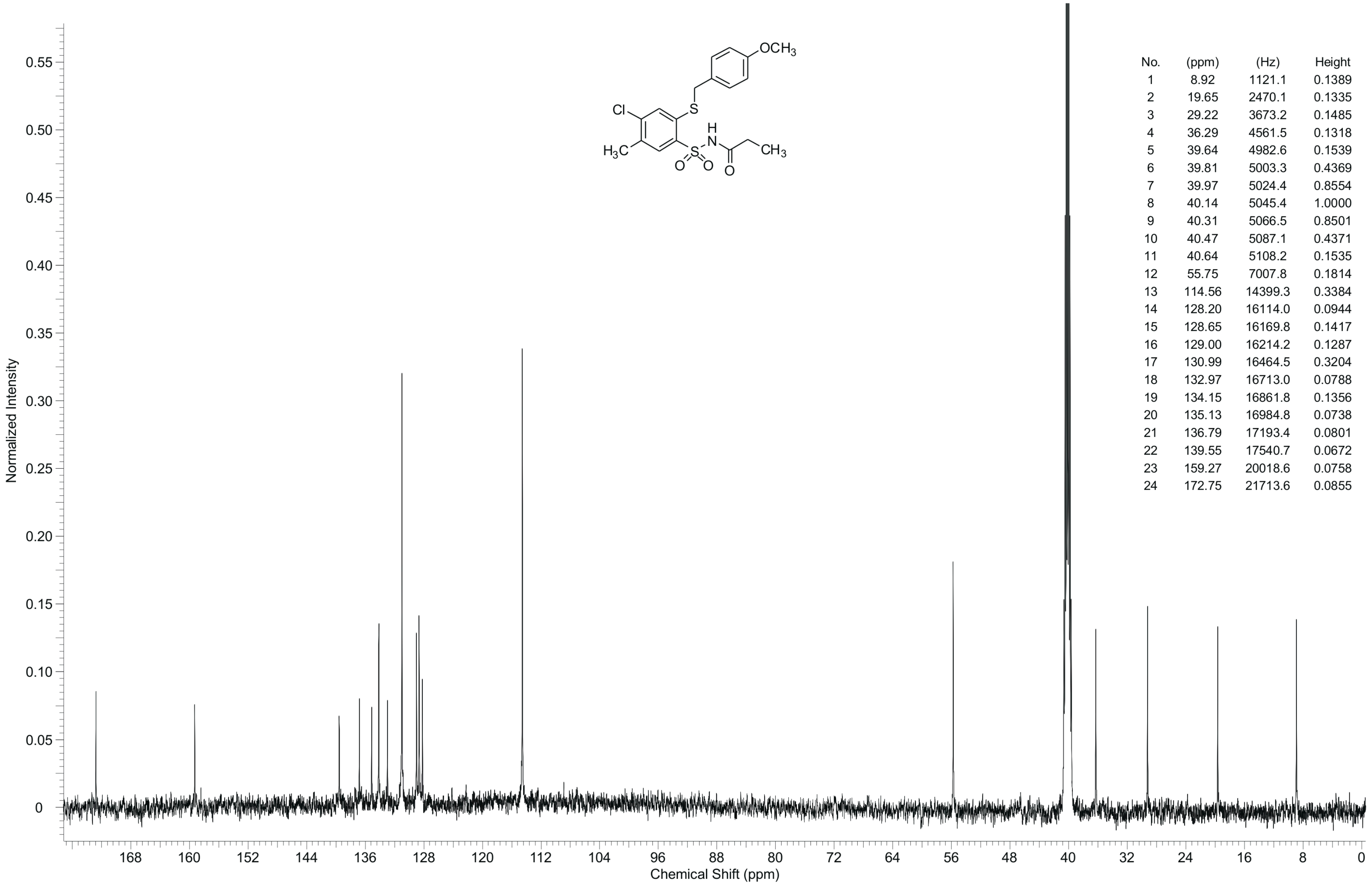

No.	(ppm)	(Hz)	Height
1	8.92	1121.1	0.1389
2	19.65	2470.1	0.1335
3	29.22	3673.2	0.1485
4	36.29	4561.5	0.1318
5	39.64	4982.6	0.1539
6	39.81	5003.3	0.4369
7	39.97	5024.4	0.8554
8	40.14	5045.4	1.0000
9	40.31	5066.5	0.8501
10	40.47	5087.1	0.4371
11	40.64	5108.2	0.1535
12	55.75	7007.8	0.1814
13	114.56	14399.3	0.3384
14	128.20	16114.0	0.0944
15	128.65	16169.8	0.1417
16	129.00	16214.2	0.1287
17	130.99	16464.5	0.3204
18	132.97	16713.0	0.0788
19	134.15	16861.8	0.1356
20	135.13	16984.8	0.0738
21	136.79	17193.4	0.0801
22	139.55	17540.7	0.0672
23	159.27	20018.6	0.0758
24	172.75	21713.6	0.0855

Figure S25. ^{13}C-NMR of compound **37** (125 MHz, DMSO-d_6).

SPECIAL LINES FOR TH= 4.04

RFL= 672.9 RFP= 499.9

INDEX	FREQ	PPM	INTENSITY
01	1704.82	8.525	73.393
02	1699.20	8.497	77.594
03	1583.07	7.916	76.024
04	1535.76	7.680	82.789
05	1487.29	7.437	78.534
06	1481.72	7.410	73.647
07	899.91	4.500	124.281
08	886.46	4.433	6.586
09	671.71	3.359	94.822
10	503.01	2.515	65.844
11	470.41	2.352	209.107
12	458.02	2.290	24.309
13	450.42	2.252	62.649
14	443.00	2.215	65.001
15	435.63	2.178	24.519
16	249.17	1.246	12.453
17	214.80	1.074	7.340
18	184.18	0.921	65.677
19	176.76	0.884	128.603
20	169.34	0.847	59.501

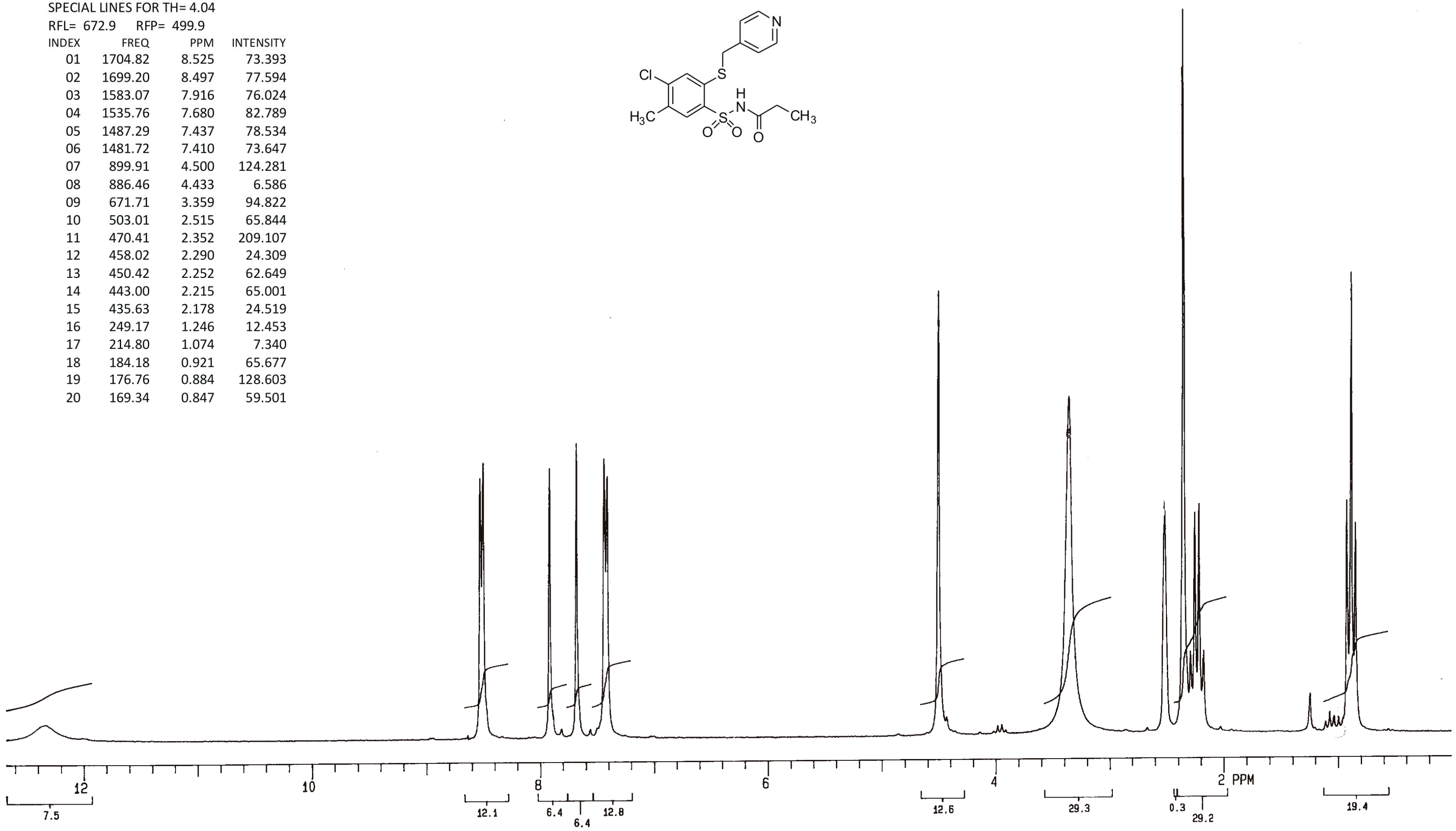

Figure S26. ^{1}H-NMR of compound **38** (200 MHz, DMSO-d_6).

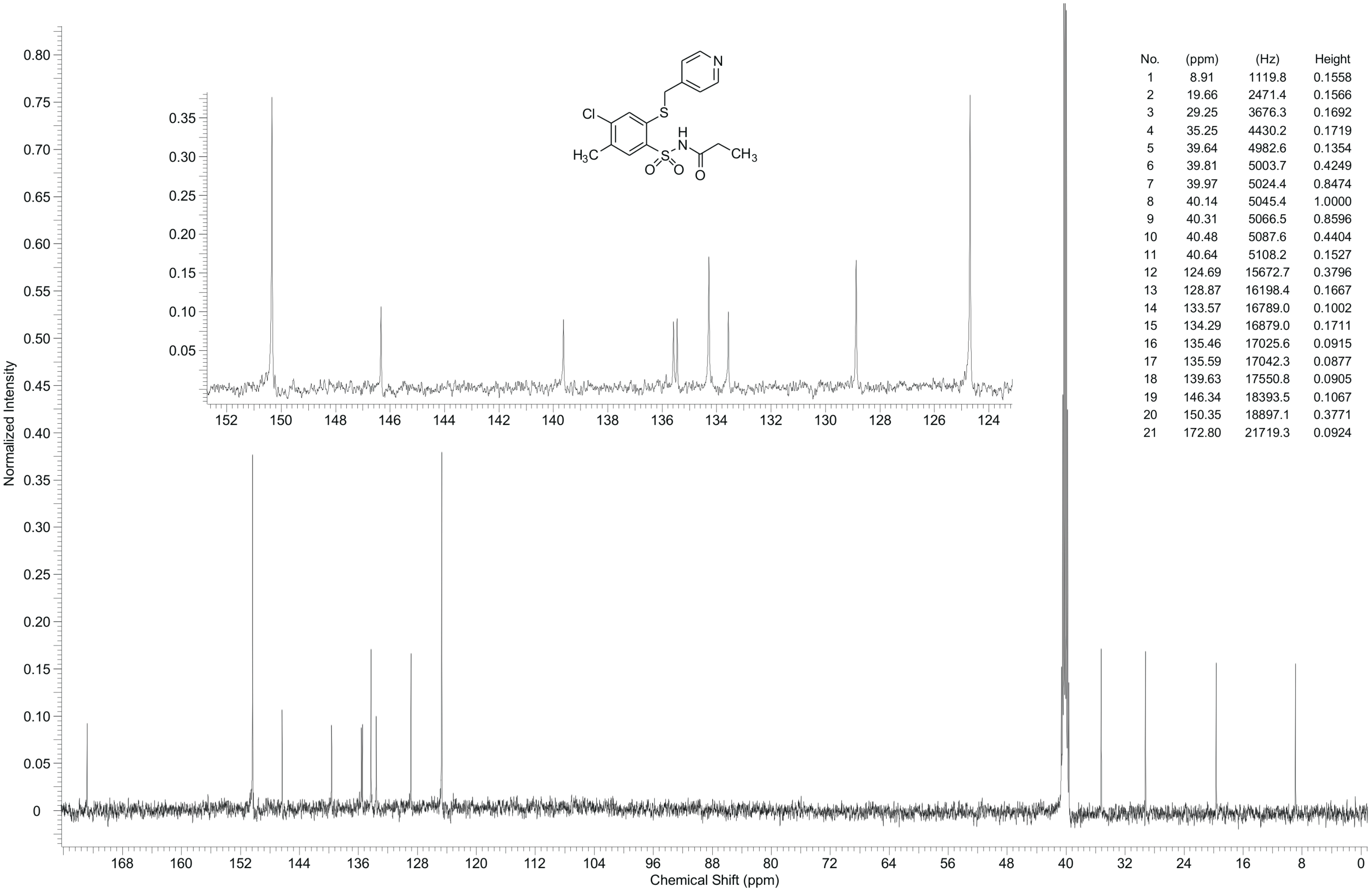

No.	(ppm)	(Hz)	Height
1	8.91	1119.8	0.1558
2	19.66	2471.4	0.1566
3	29.25	3676.3	0.1692
4	35.25	4430.2	0.1719
5	39.64	4982.6	0.1354
6	39.81	5003.7	0.4249
7	39.97	5024.4	0.8474
8	40.14	5045.4	1.0000
9	40.31	5066.5	0.8596
10	40.48	5087.6	0.4404
11	40.64	5108.2	0.1527
12	124.69	15672.7	0.3796
13	128.87	16198.4	0.1667
14	133.57	16789.0	0.1002
15	134.29	16879.0	0.1711
16	135.46	17025.6	0.0915
17	135.59	17042.3	0.0877
18	139.63	17550.8	0.0905
19	146.34	18393.5	0.1067
20	150.35	18897.1	0.3771
21	172.80	21719.3	0.0924

Figure S27. ^{13}C-NMR of compound **38** (125 MHz, DMSO-d_6).

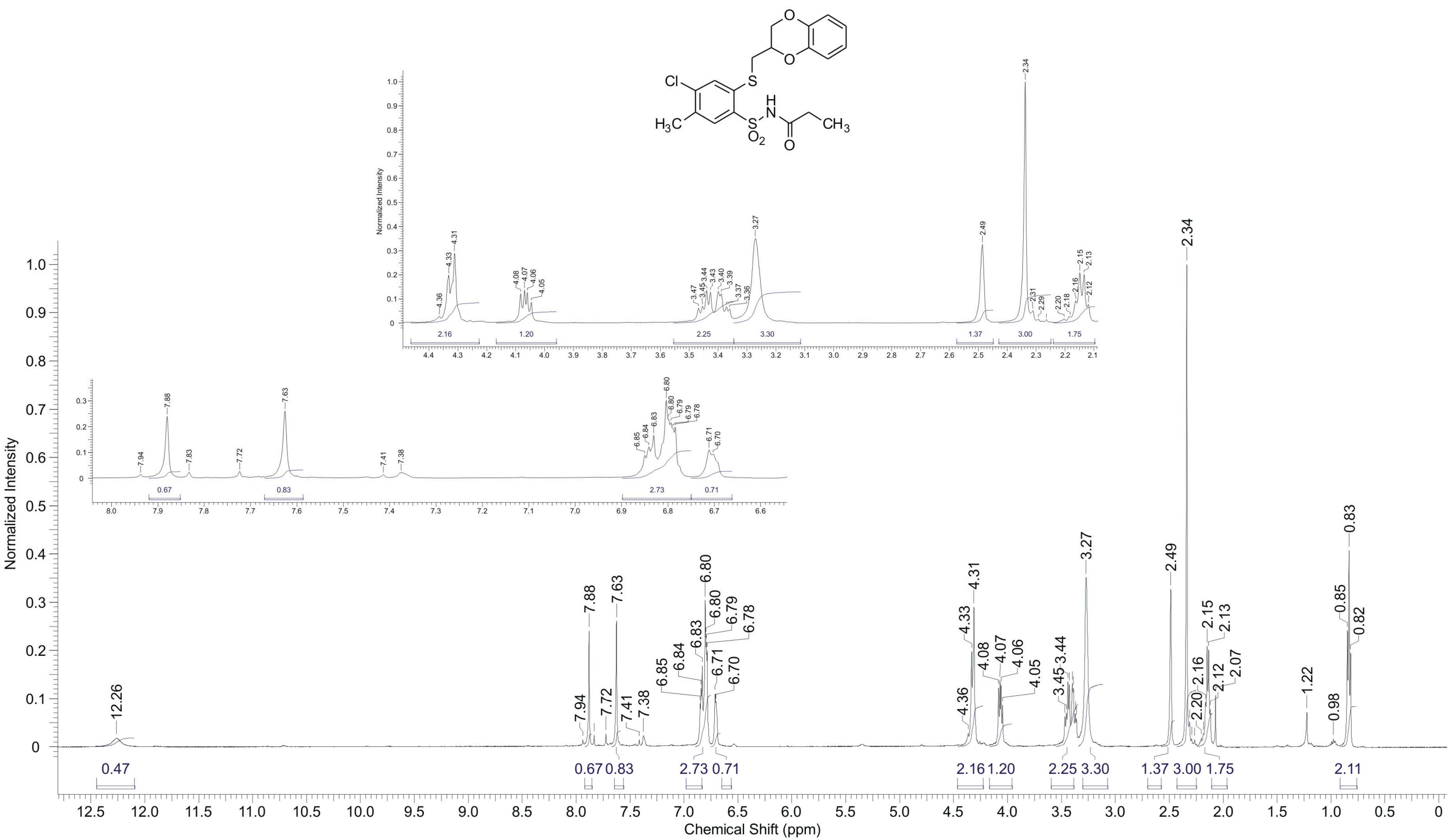

Figure S28. ^{1}H-NMR of compound **40** (500 MHz, DMSO-d_6).

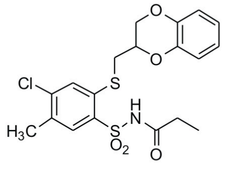

SPECIAL LINES FOR TH=8.10
RFL= 2095.7 RFP= 0

INDEX	FREQ	PPM	INTENSITY
01	8777.1	174.532	11.363
02	7190.2	142.976	21.949
03	7169.6	142.568	17.783
04	6936.6	137.933	10.141
05	6790.2	135.022	18.446
06	6699.9	133.226	27.747
07	6675.1	132.733	20.518
08	6478.0	128.815	23.502
09	6124.7	121.789	36.409
10	6118.4	121.663	36.730
11	5898.6	117.294	38.744
12	5892.5	117.173	40.607
13	3624.8	72.078	32.263
14	3330.5	66.226	23.024
15	2059.5	40.953	22.225
16	2038.1	40.528	71.697
17	2017.0	40.107	135.496
18	1996.0	39.691	161.047
19	1987.6	39.523	16.195
20	1975.2	39.276	136.646
21	1964.7	39.068	9.909
22	1954.3	38.862	69.469
23	1932.7	38.431	23.722
24	1673.6	33.279	21.165
25	1506.3	29.953	23.840
26	970.9	19.306	32.158
27	458.1	9.110	25.840

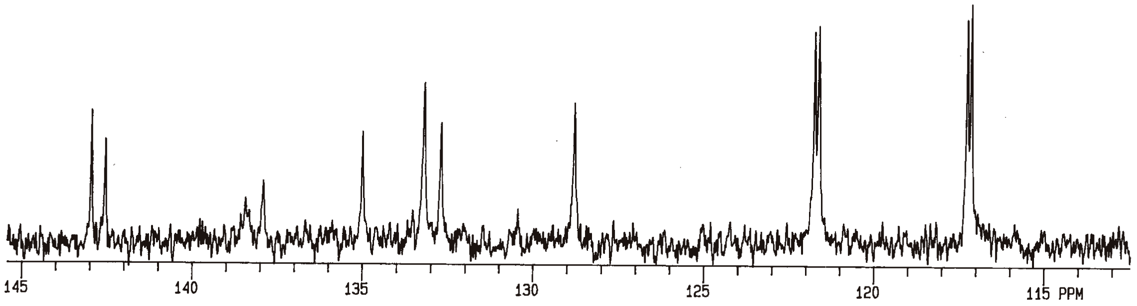

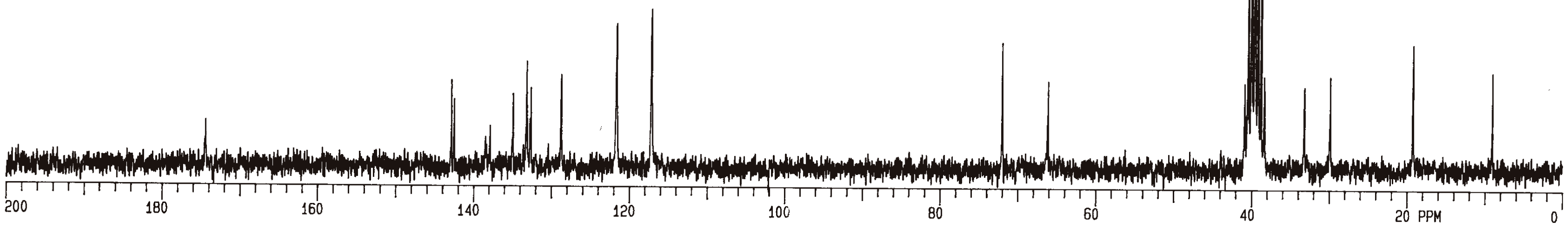

Figure S29. ^{13}C-NMR of compound **40** (50 MHz, DMSO-d_6).

SPECIAL LINES FOR TH= 7.91
RFL= 672.9 RFP= 499.9

INDEX	FREQ	PPM	INTENSITY
01	2451.57	12.259	38.431
02	1586.26	7.932	54.528
03	1568.41	7.843	11.329
04	1523.00	7.616	51.697
05	1516.79	7.585	10.285
06	1501.85	7.510	10.489
07	1477.03	7.386	12.915
08	1422.64	7.114	81.715
09	1415.58	7.079	20.236
10	1407.74	7.040	21.689
11	1215.77	6.080	136.201
12	1008.86	5.045	8.574
13	904.68	4.524	9.541
14	890.35	4.452	21.283
15	877.83	4.390	84.339
16	863.86	4.320	18.504
17	851.11	4.256	7.963
18	672.41	3.362	88.762
19	502.08	2.511	145.936
20	486.77	2.434	24.703
21	475.51	2.378	153.098
22	456.96	2.285	23.316
23	449.67	2.249	44.060
24	442.12	2.211	44.891
25	434.83	2.174	19.912
26	219.61	1.098	11.160
27	183.72	0.919	45.531
28	176.53	0.883	81.185
29	169.11	0.846	40.131

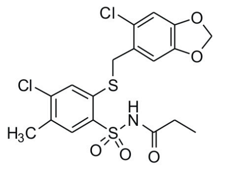

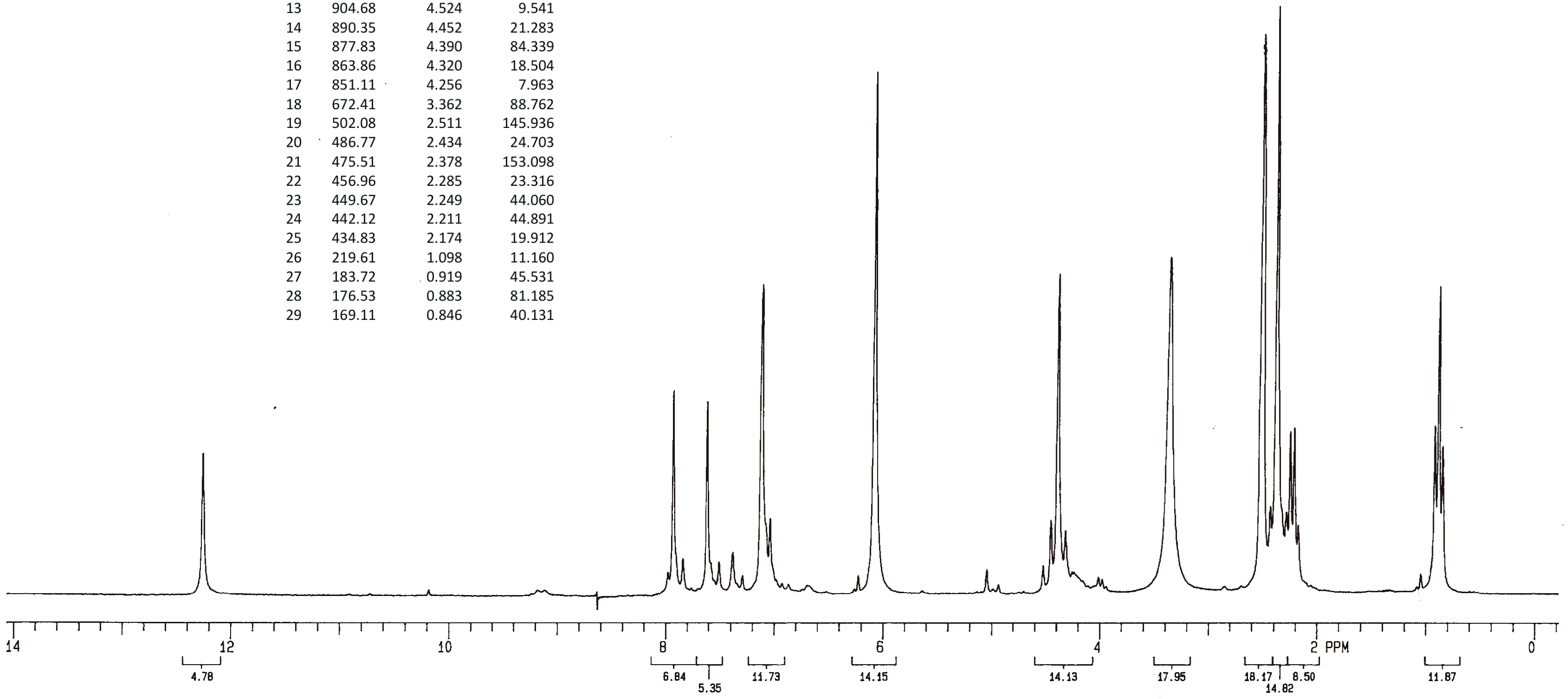

Figure S30. ^{1}H-NMR of compound **41** (200 MHz, DMSO-d_6).

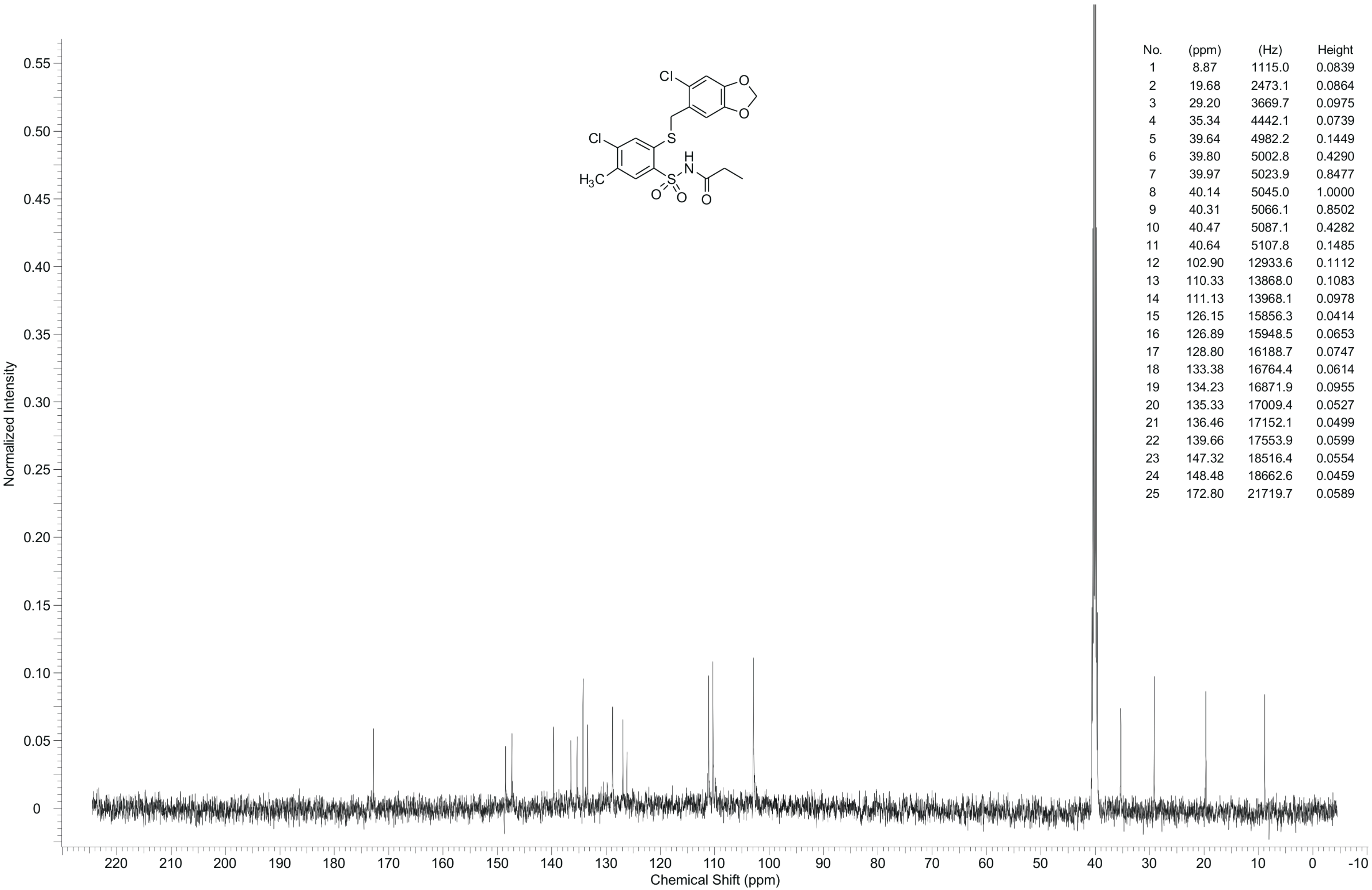

No.	(ppm)	(Hz)	Height
1	8.87	1115.0	0.0839
2	19.68	2473.1	0.0864
3	29.20	3669.7	0.0975
4	35.34	4442.1	0.0739
5	39.64	4982.2	0.1449
6	39.80	5002.8	0.4290
7	39.97	5023.9	0.8477
8	40.14	5045.0	1.0000
9	40.31	5066.1	0.8502
10	40.47	5087.1	0.4282
11	40.64	5107.8	0.1485
12	102.90	12933.6	0.1112
13	110.33	13868.0	0.1083
14	111.13	13968.1	0.0978
15	126.15	15856.3	0.0414
16	126.89	15948.5	0.0653
17	128.80	16188.7	0.0747
18	133.38	16764.4	0.0614
19	134.23	16871.9	0.0955
20	135.33	17009.4	0.0527
21	136.46	17152.1	0.0499
22	139.66	17553.9	0.0599
23	147.32	18516.4	0.0554
24	148.48	18662.6	0.0459
25	172.80	21719.7	0.0589

Figure S31. ^{13}C-NMR of compound **41** (125 MHz, DMSO-d_6).

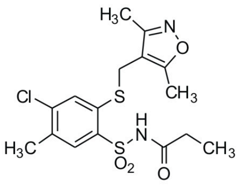

SPECIAL LINES FOR TH= 11.39
RFL= 672.9 RFP= 499.9

INDEX	FREQ	PPM	INTENSITY
01	2456.03	12.282	27.652
02	1588.12	7.942	32.928
03	1542.66	7.714	33.793
04	855.98	4.280	57.337
05	678.10	3.391	20.145
06	503.34	2.517	64.837
07	478.30	2.392	116.867
08	473.32	2.367	122.847
09	449.77	2.249	123.007
10	440.26	2.202	38.814
11	434.36	2.172	20.679
12	185.75	0.929	33.250
13	178.57	0.893	55.199
14	171.48	0.858	29.041

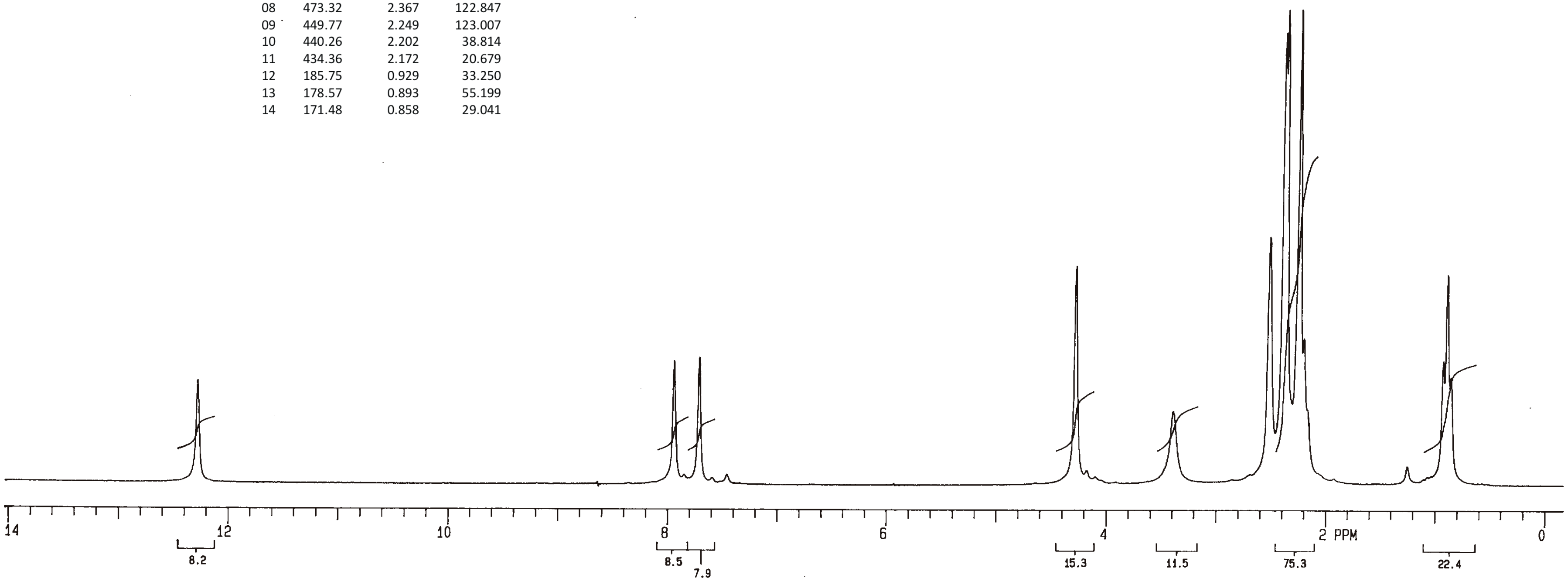

Figure S32. ¹H-NMR of compound **42** (200 MHz, DMSO-*d*6).

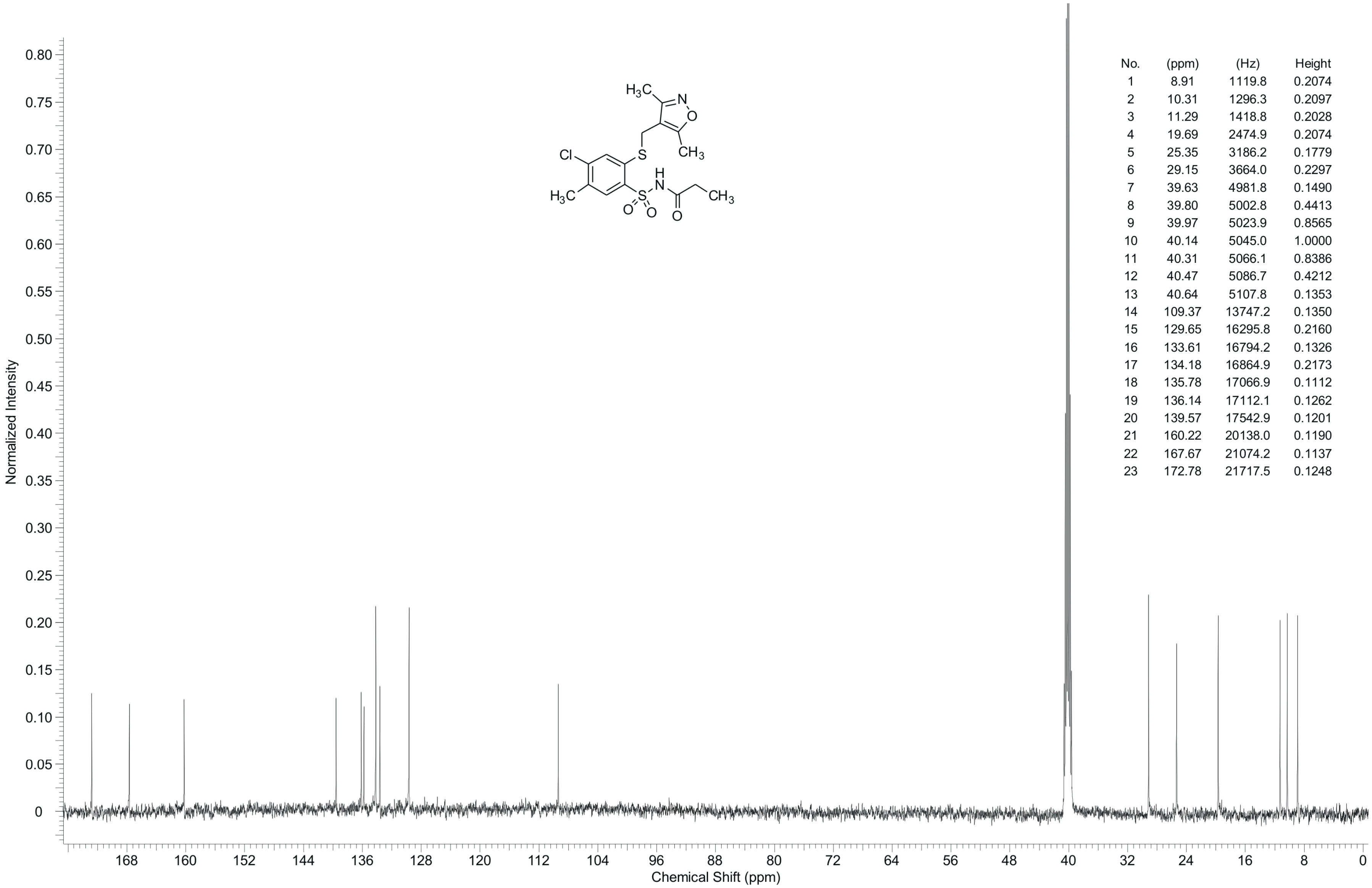

No.	(ppm)	(Hz)	Height
1	8.91	1119.8	0.2074
2	10.31	1296.3	0.2097
3	11.29	1418.8	0.2028
4	19.69	2474.9	0.2074
5	25.35	3186.2	0.1779
6	29.15	3664.0	0.2297
7	39.63	4981.8	0.1490
8	39.80	5002.8	0.4413
9	39.97	5023.9	0.8565
10	40.14	5045.0	1.0000
11	40.31	5066.1	0.8386
12	40.47	5086.7	0.4212
13	40.64	5107.8	0.1353
14	109.37	13747.2	0.1350
15	129.65	16295.8	0.2160
16	133.61	16794.2	0.1326
17	134.18	16864.9	0.2173
18	135.78	17066.9	0.1112
19	136.14	17112.1	0.1262
20	139.57	17542.9	0.1201
21	160.22	20138.0	0.1190
22	167.67	21074.2	0.1137
23	172.78	21717.5	0.1248

Figure S33. ^{13}C-NMR of compound **42** (125 MHz, DMSO-d_6).

SPECIAL LINES FOR TH= 6.88
RFL= 672.9 RFP= 499.9

INDEX	FREQ	PPM	INTENSITY
01	2456.73	12.285	36.686
02	1583.30	7.917	49.896
03	1527.41	7.638	43.053
04	1489.43	7.448	26.588
05	1482.47	7.413	55.621
06	1475.18	7.377	25.002
07	1469.15	7.347	48.391
08	1451.55	7.309	53.734
09	1455.11	7.276	21.058
10	885.71	4.429	85.758
11	694.34	3.472	10.624
12	687.25	3.437	11.965
13	680.29	3.402	8.710
14	511.41	2.557	9.193
15	502.42	2.512	37.055
16	491.05	2.456	7.015
17	471.34	2.357	139.142
18	282.46	1.412	10.164
19	213.58	1.068	12.729
20	206.50	1.033	8.364
21	196.47	0.982	114.910
22	189.75	0.949	116.193

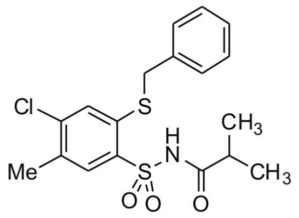

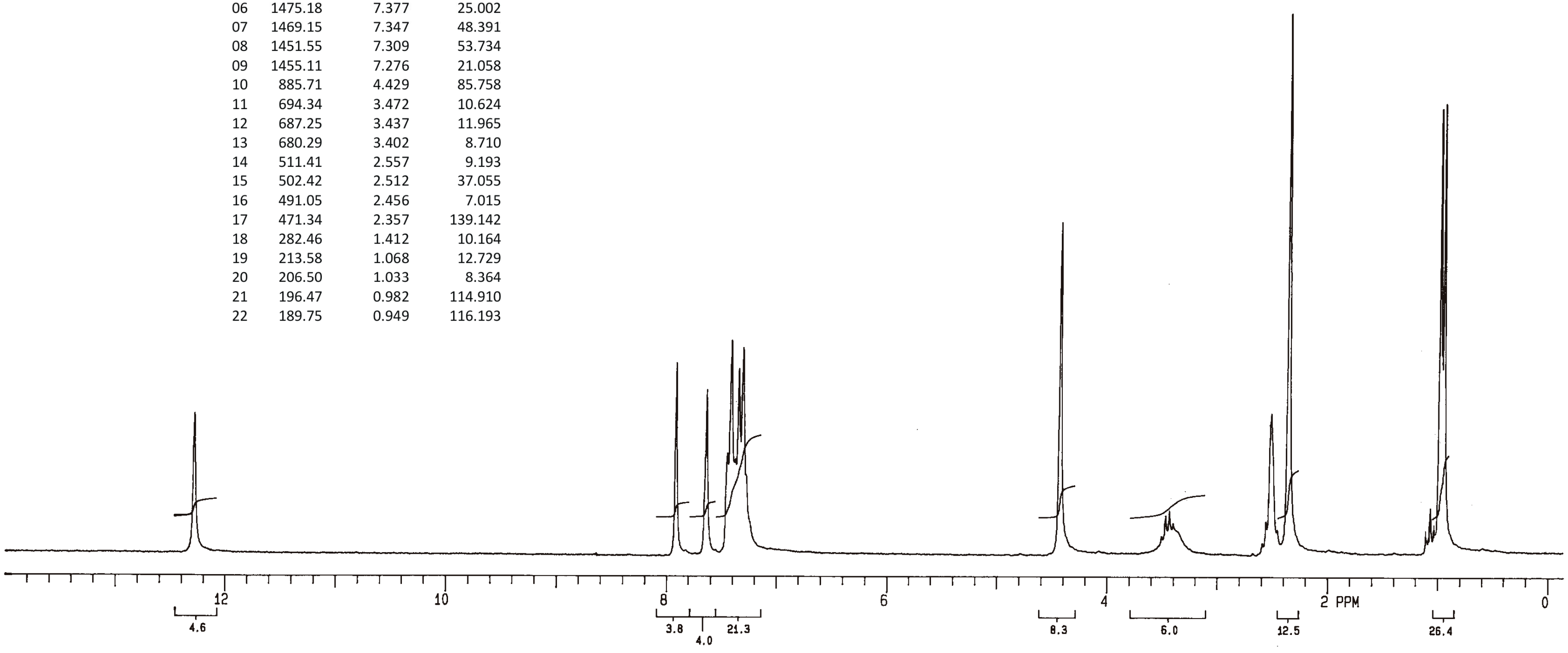

Figure S34. ^{1}H-NMR of compound **43** (200 MHz, DMSO-d_6).

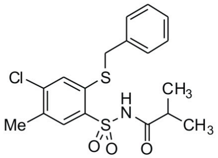

SPECIAL LINES FOR TH= 8.24
RFL= 1823.1 RFP= 0

INDEX	FREQ	PPM	INTENSITY
01	9097.3	180.899	15.851
02	7271.5	144.594	18.862
03	7131.4	141.807	17.839
04	7111.2	141.405	19.846
05	7041.8	140.026	13.106
06	7006.0	139.314	21.915
07	6940.0	138.002	21.828
08	6780.8	134.836	78.306
09	6750.3	134.229	63.198
10	6711.1	133.449	24.493
11	6697.6	133.182	38.846
12	2337.1	46.472	14.721
13	2316.0	46.054	56.129
14	2295.0	45.635	106.910
15	2286.7	45.471	10.471
16	2274.1	45.221	130.887
17	2265.7	45.053	11.108
18	2253.1	44.802	111.267
19	2244.2	44.626	10.771
20	2232.0	44.384	56.775
21	2211.1	43.967	19.402
22	2107.2	41.901	30.200
23	2002.0	39.810	25.140
24	1239.9	24.656	36.546
25	1214.1	24.142	53.195

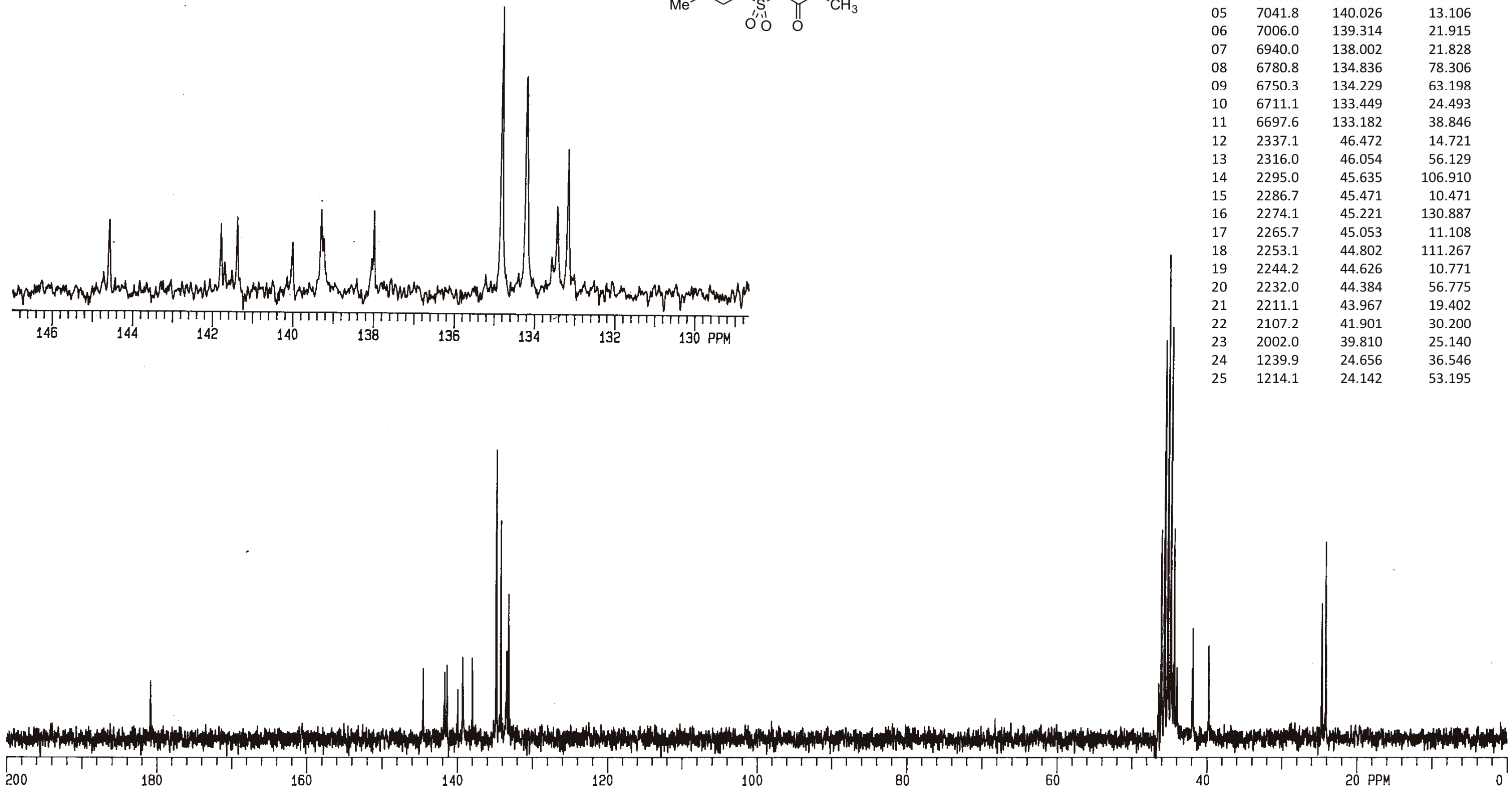

Figure S35. ^{13}C-NMR of compound **43** (50 MHz, DMSO-d_6).

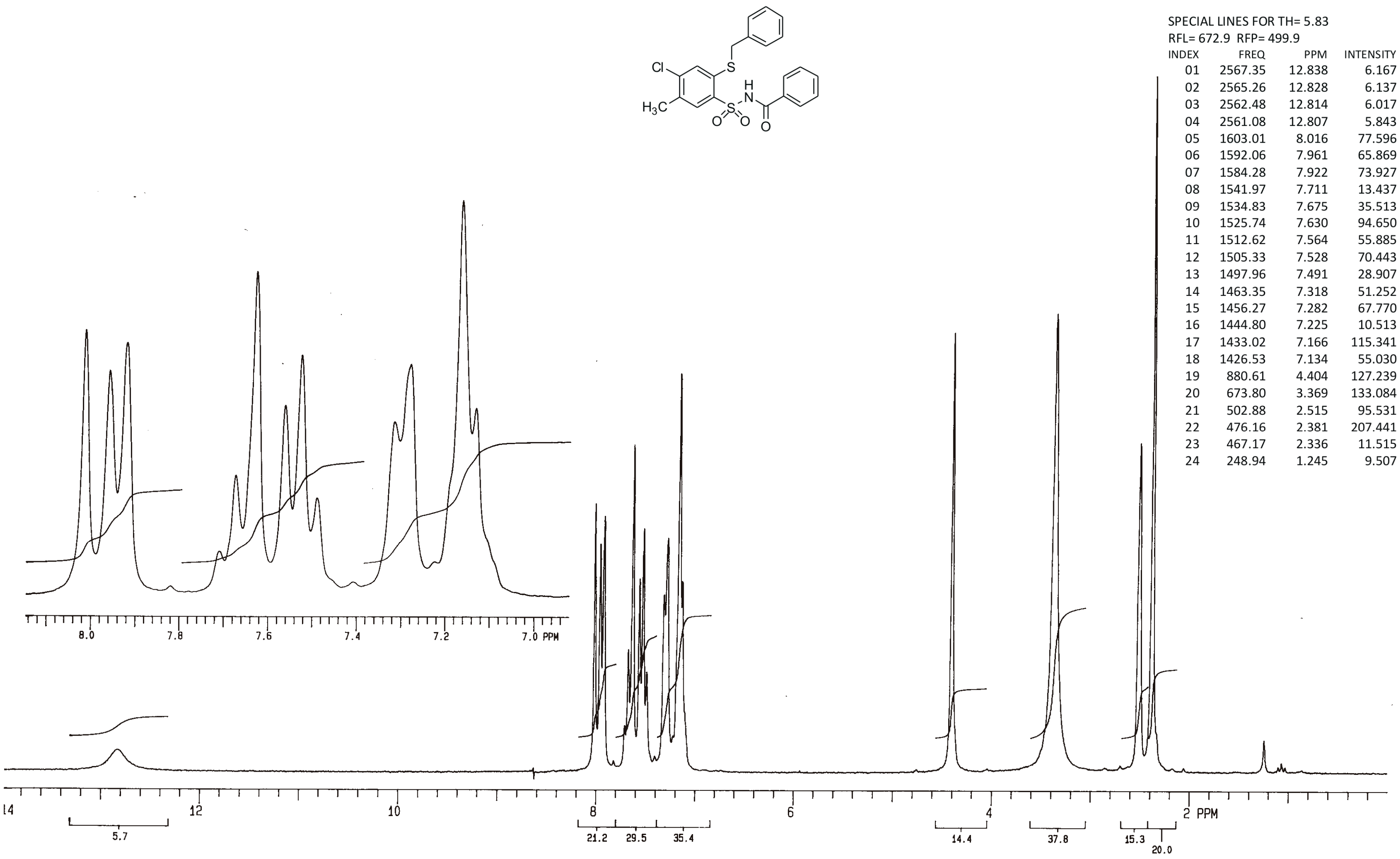

SPECIAL LINES FOR TH= 5.83
RFL= 672.9 RFP= 499.9

INDEX	FREQ	PPM	INTENSITY
01	2567.35	12.838	6.167
02	2565.26	12.828	6.137
03	2562.48	12.814	6.017
04	2561.08	12.807	5.843
05	1603.01	8.016	77.596
06	1592.06	7.961	65.869
07	1584.28	7.922	73.927
08	1541.97	7.711	13.437
09	1534.83	7.675	35.513
10	1525.74	7.630	94.650
11	1512.62	7.564	55.885
12	1505.33	7.528	70.443
13	1497.96	7.491	28.907
14	1463.35	7.318	51.252
15	1456.27	7.282	67.770
16	1444.80	7.225	10.513
17	1433.02	7.166	115.341
18	1426.53	7.134	55.030
19	880.61	4.404	127.239
20	673.80	3.369	133.084
21	502.88	2.515	95.531
22	476.16	2.381	207.441
23	467.17	2.336	11.515
24	248.94	1.245	9.507

Figure S36. ^{1}H-NMR of compound **45** (200 MHz, DMSO-d_6).

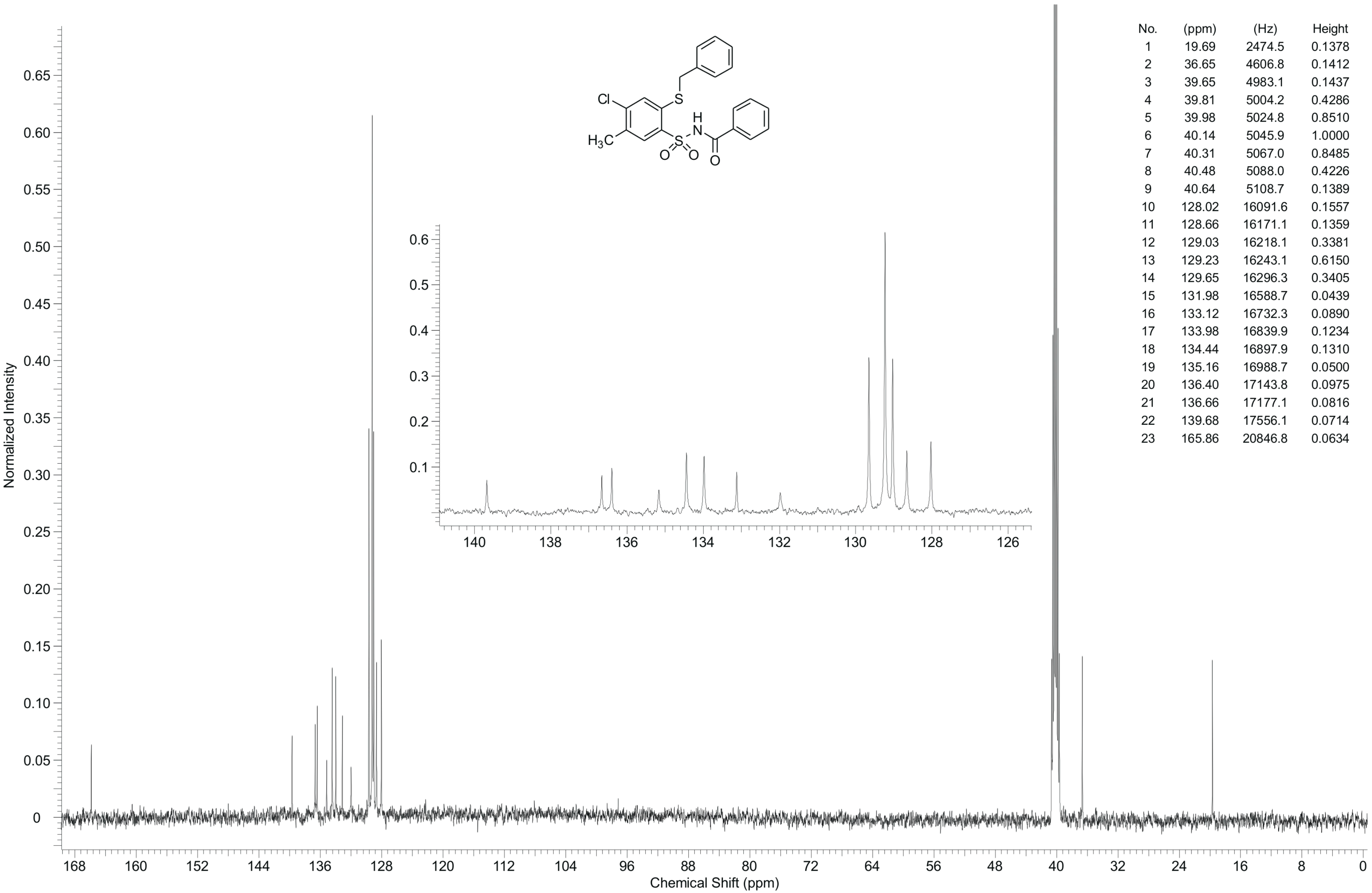

No.	(ppm)	(Hz)	Height
1	19.69	2474.5	0.1378
2	36.65	4606.8	0.1412
3	39.65	4983.1	0.1437
4	39.81	5004.2	0.4286
5	39.98	5024.8	0.8510
6	40.14	5045.9	1.0000
7	40.31	5067.0	0.8485
8	40.48	5088.0	0.4226
9	40.64	5108.7	0.1389
10	128.02	16091.6	0.1557
11	128.66	16171.1	0.1359
12	129.03	16218.1	0.3381
13	129.23	16243.1	0.6150
14	129.65	16296.3	0.3405
15	131.98	16588.7	0.0439
16	133.12	16732.3	0.0890
17	133.98	16839.9	0.1234
18	134.44	16897.9	0.1310
19	135.16	16988.7	0.0500
20	136.40	17143.8	0.0975
21	136.66	17177.1	0.0816
22	139.68	17556.1	0.0714
23	165.86	20846.8	0.0634

Figure S37. ^{13}C-NMR of compound **45** (125 MHz, DMSO-d_6).

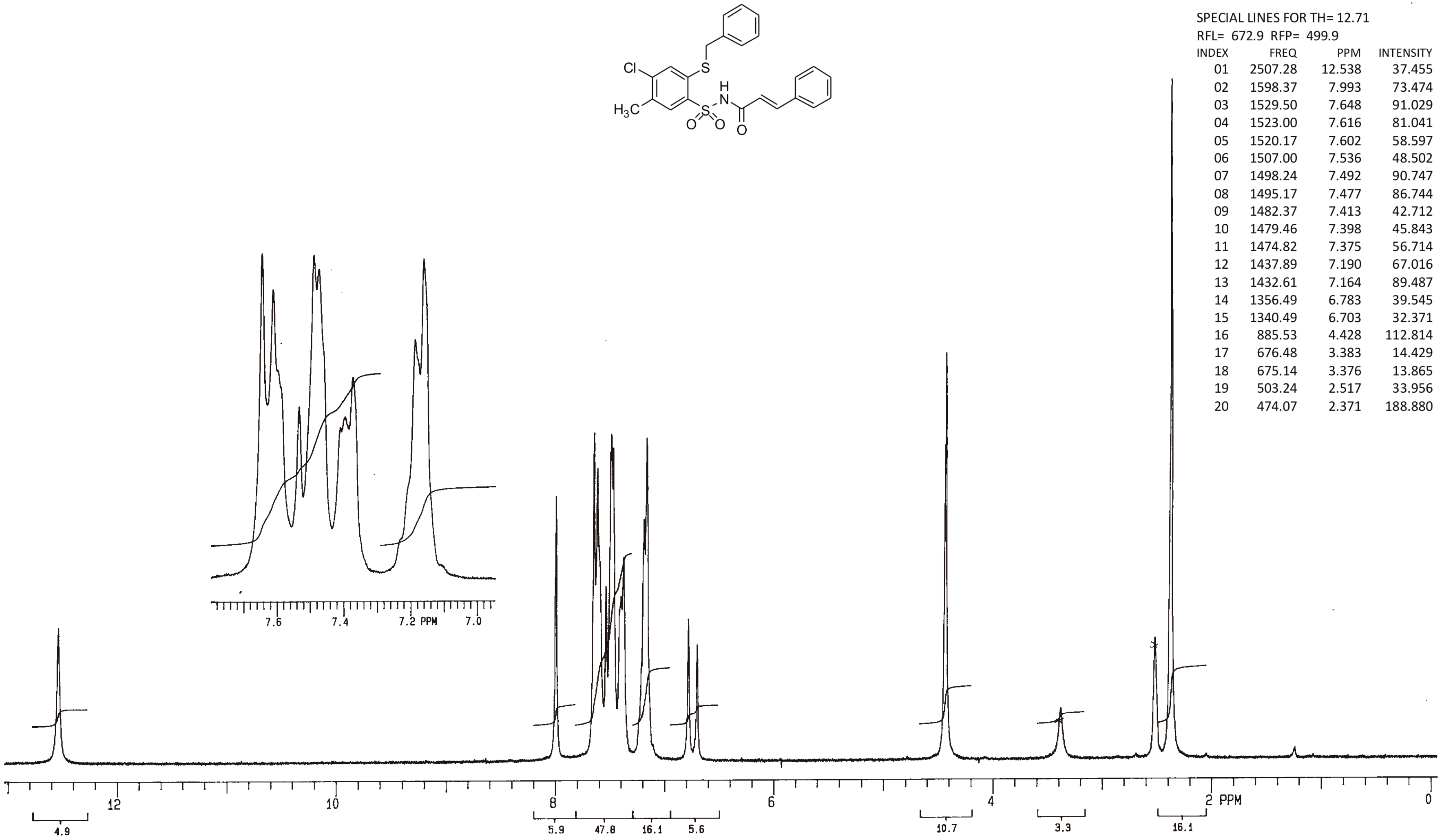

SPECIAL LINES FOR TH= 12.71
RFL= 672.9 RFP= 499.9

INDEX	FREQ	PPM	INTENSITY
01	2507.28	12.538	37.455
02	1598.37	7.993	73.474
03	1529.50	7.648	91.029
04	1523.00	7.616	81.041
05	1520.17	7.602	58.597
06	1507.00	7.536	48.502
07	1498.24	7.492	90.747
08	1495.17	7.477	86.744
09	1482.37	7.413	42.712
10	1479.46	7.398	45.843
11	1474.82	7.375	56.714
12	1437.89	7.190	67.016
13	1432.61	7.164	89.487
14	1356.49	6.783	39.545
15	1340.49	6.703	32.371
16	885.53	4.428	112.814
17	676.48	3.383	14.429
18	675.14	3.376	13.865
19	503.24	2.517	33.956
20	474.07	2.371	188.880

Figure S38. ^{1}H-NMR of compound **46** (200 MHz, DMSO-d_6).

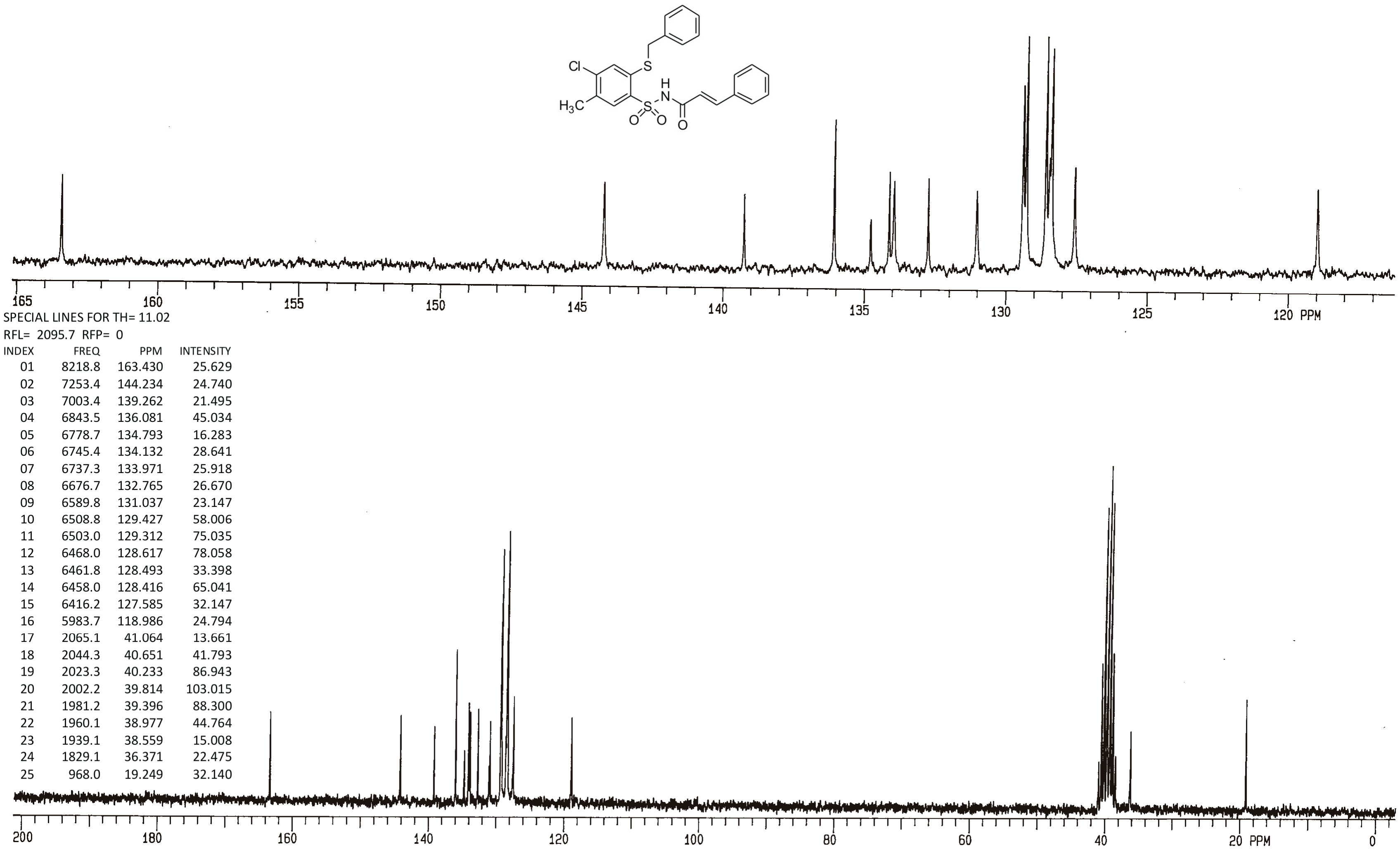

SPECIAL LINES FOR TH= 11.02
RFL= 2095.7 RFP= 0

INDEX	FREQ	PPM	INTENSITY
01	8218.8	163.430	25.629
02	7253.4	144.234	24.740
03	7003.4	139.262	21.495
04	6843.5	136.081	45.034
05	6778.7	134.793	16.283
06	6745.4	134.132	28.641
07	6737.3	133.971	25.918
08	6676.7	132.765	26.670
09	6589.8	131.037	23.147
10	6508.8	129.427	58.006
11	6503.0	129.312	75.035
12	6468.0	128.617	78.058
13	6461.8	128.493	33.398
14	6458.0	128.416	65.041
15	6416.2	127.585	32.147
16	5983.7	118.986	24.794
17	2065.1	41.064	13.661
18	2044.3	40.651	41.793
19	2023.3	40.233	86.943
20	2002.2	39.814	103.015
21	1981.2	39.396	88.300
22	1960.1	38.977	44.764
23	1939.1	38.559	15.008
24	1829.1	36.371	22.475
25	968.0	19.249	32.140

Figure S39. ^{13}C-NMR of compound **46** (50 MHz, DMSO-d_6).

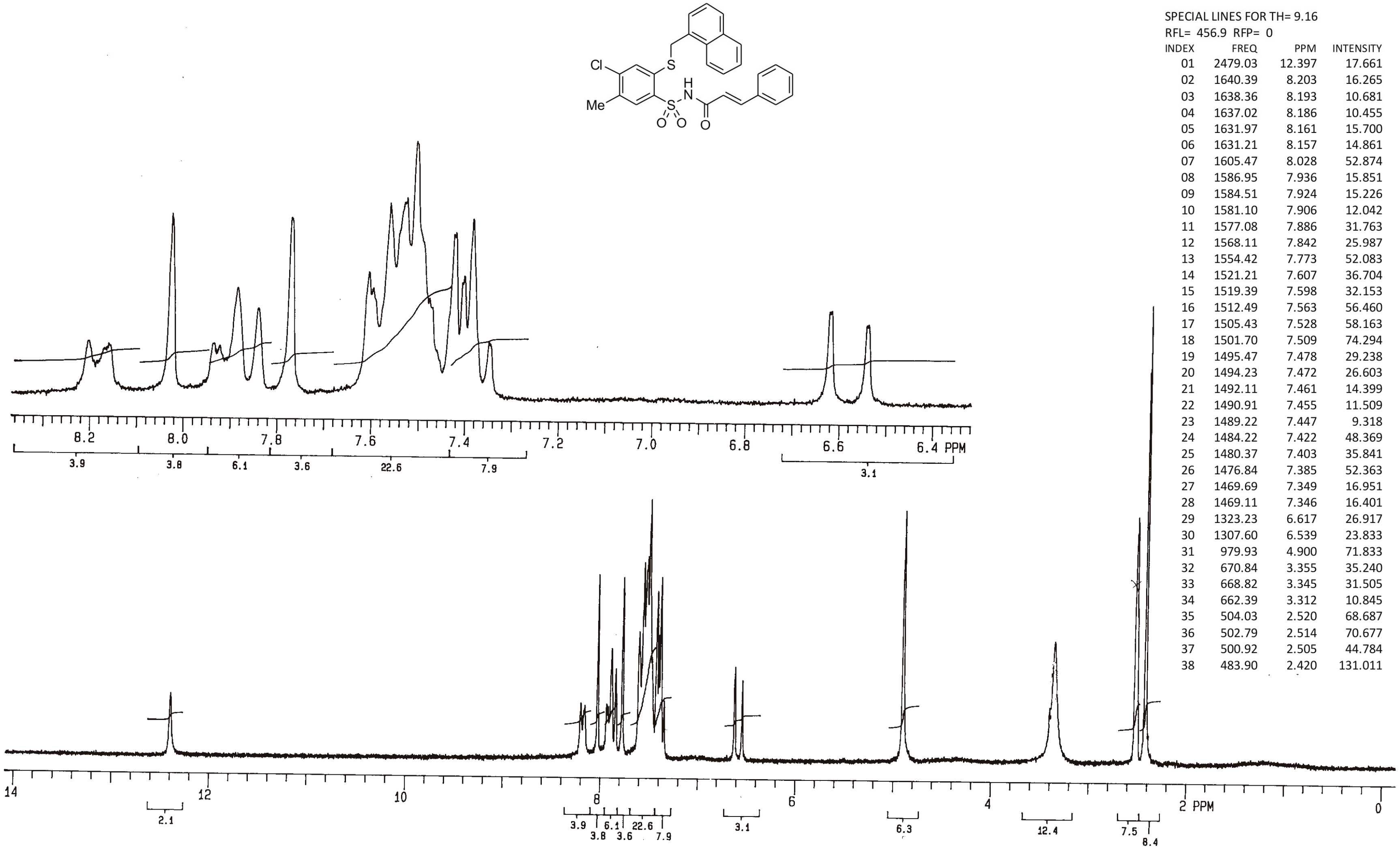

SPECIAL LINES FOR TH= 9.16
RFL= 456.9 RFP= 0

INDEX	FREQ	PPM	INTENSITY
01	2479.03	12.397	17.661
02	1640.39	8.203	16.265
03	1638.36	8.193	10.681
04	1637.02	8.186	10.455
05	1631.97	8.161	15.700
06	1631.21	8.157	14.861
07	1605.47	8.028	52.874
08	1586.95	7.936	15.851
09	1584.51	7.924	15.226
10	1581.10	7.906	12.042
11	1577.08	7.886	31.763
12	1568.11	7.842	25.987
13	1554.42	7.773	52.083
14	1521.21	7.607	36.704
15	1519.39	7.598	32.153
16	1512.49	7.563	56.460
17	1505.43	7.528	58.163
18	1501.70	7.509	74.294
19	1495.47	7.478	29.238
20	1494.23	7.472	26.603
21	1492.11	7.461	14.399
22	1490.91	7.455	11.509
23	1489.22	7.447	9.318
24	1484.22	7.422	48.369
25	1480.37	7.403	35.841
26	1476.84	7.385	52.363
27	1469.69	7.349	16.951
28	1469.11	7.346	16.401
29	1323.23	6.617	26.917
30	1307.60	6.539	23.833
31	979.93	4.900	71.833
32	670.84	3.355	35.240
33	668.82	3.345	31.505
34	662.39	3.312	10.845
35	504.03	2.520	68.687
36	502.79	2.514	70.677
37	500.92	2.505	44.784
38	483.90	2.420	131.011

Figure S40. ^{1}H-NMR of compound **47** (200 MHz, DMSO-d_6).

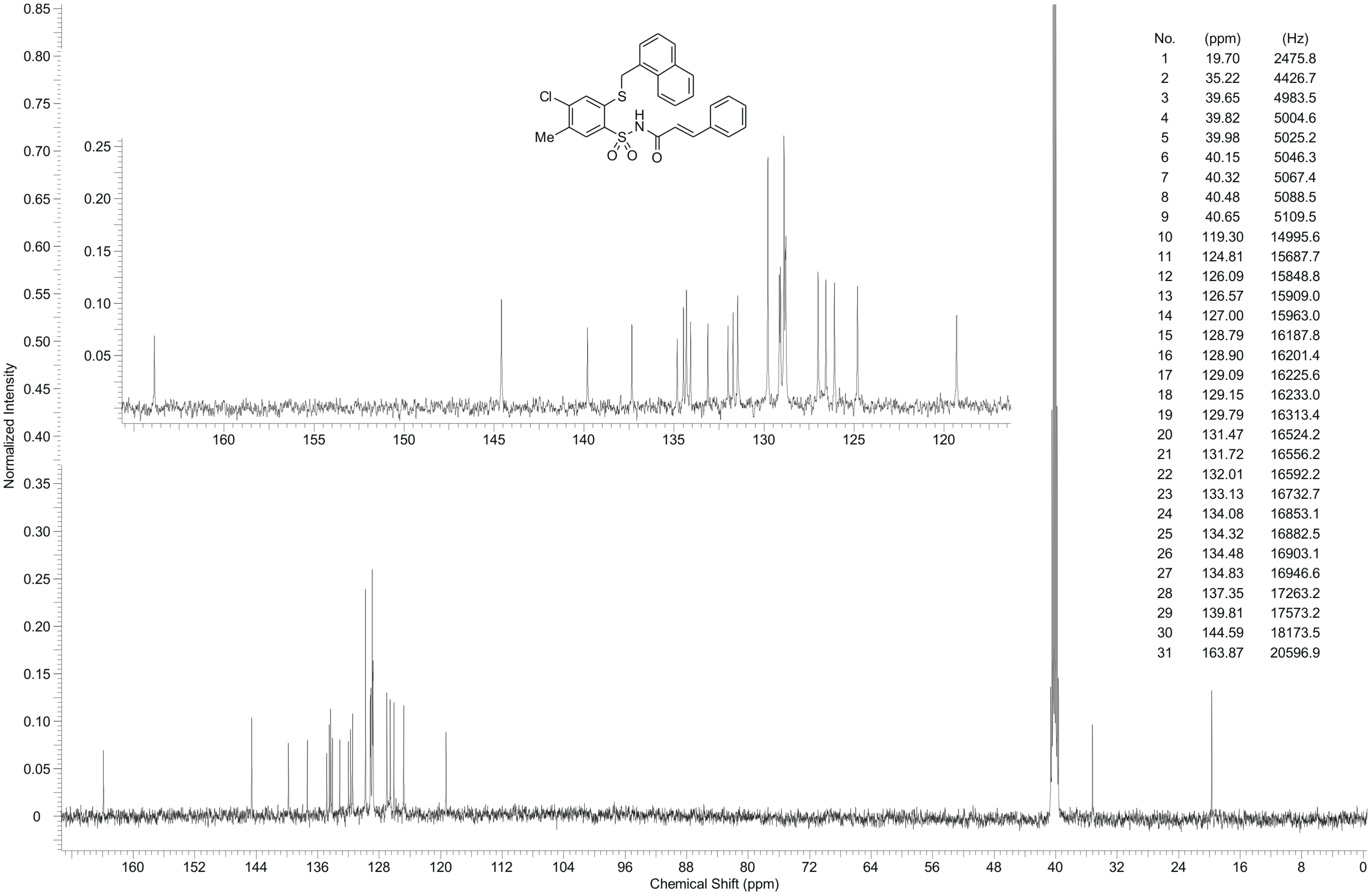

Figure S41. ^{13}C-NMR of compound **47** (125 MHz, DMSO-d_6)
